# Analysis of Barley Leaf Epidermis and Extrahaustorial Proteomes During Powdery Mildew Infection Reveals That the PR5 Thaumatin-Like Protein TLP5 Is Required for Susceptibility Towards *Blumeria graminis* f. sp. *hordei*

**DOI:** 10.3389/fpls.2019.01138

**Published:** 2019-10-30

**Authors:** Sebastien Lambertucci, Kate Mary Orman, Shaoli Das Gupta, James Paul Fisher, Snehi Gazal, Ryan Joshua Williamson, Rainer Cramer, Laurence Véronique Bindschedler

**Affiliations:** ^1^School of Biological Sciences, Royal Holloway University of London, Egham, United Kingdom; ^2^Department of Chemistry, University of Reading, Reading, United Kingdom

**Keywords:** Proteomics, biotrophic fungus, barley powdery mildew, haustorium, susceptibility factors

## Abstract

Powdery mildews are biotrophic pathogens causing fungal diseases in many economically important crops, including cereals, which are affected by *Blumeria graminis*. Powdery mildews only invade the epidermal cell layer of leaf tissues, in which they form haustorial structures. Haustoria are at the center of the biotrophic interaction by taking up nutrients from the host and by delivering effectors in the invaded cells to jeopardize plant immunity. Haustoria are composed of a fungal core delimited by a haustorial plasma membrane and cell wall. Surrounding these is the extrahaustorial complex, of which the extrahaustorial membrane is of plant origin. Although haustoria transcriptomes and proteomes have been investigated for *Blumeria*, the proteomes of barley epidermis upon infection and the barley components of the extrahaustorial complex remains unexplored. When comparing proteomes of infected and non-infected epidermis, several classical pathogenesis-related (PR) proteins were more abundant in infected epidermis. These included peroxidases, chitinases, cysteine-rich venom secreted proteins/PR1 and two thaumatin-like PR5 protein isoforms, of which TLP5 was previously shown to interact with the *Blumeria* effector BEC1054 (CSEP0064). Against expectations, transient *TLP5* gene silencing suggested that TLP5 does not contribute to resistance but modulates susceptibility towards *B. graminis*. In a second proteomics comparison, haustorial structures were enriched from infected epidermal strips to identify plant proteins closely associated with the extrahaustorial complex. In these haustoria-enriched samples, relative abundances were higher for several V-type ATP synthase/ATPase subunits, suggesting the generation of proton gradients in the extrahaustorial space. Other haustoria-associated proteins included secreted or membrane proteins such as a PIP2 aquaporin, an early nodulin-like protein 9, an aspartate protease and other proteases, a lipase, and a lipid transfer protein, all of which are potential modulators of immunity, or the targets of pathogen effectors. Moreover, the ER BIP-like HSP70, may link ER stress responses and the idea of ER-like properties previously attributed to the extrahaustorial membrane. This initial investigation exploring the barley proteomes of *Blumeria*-infected tissues and haustoria, associated with a transient gene silencing approach, is invaluable to gain first insight of key players of resistance and susceptibility.

## Introduction

**Figure 1:**
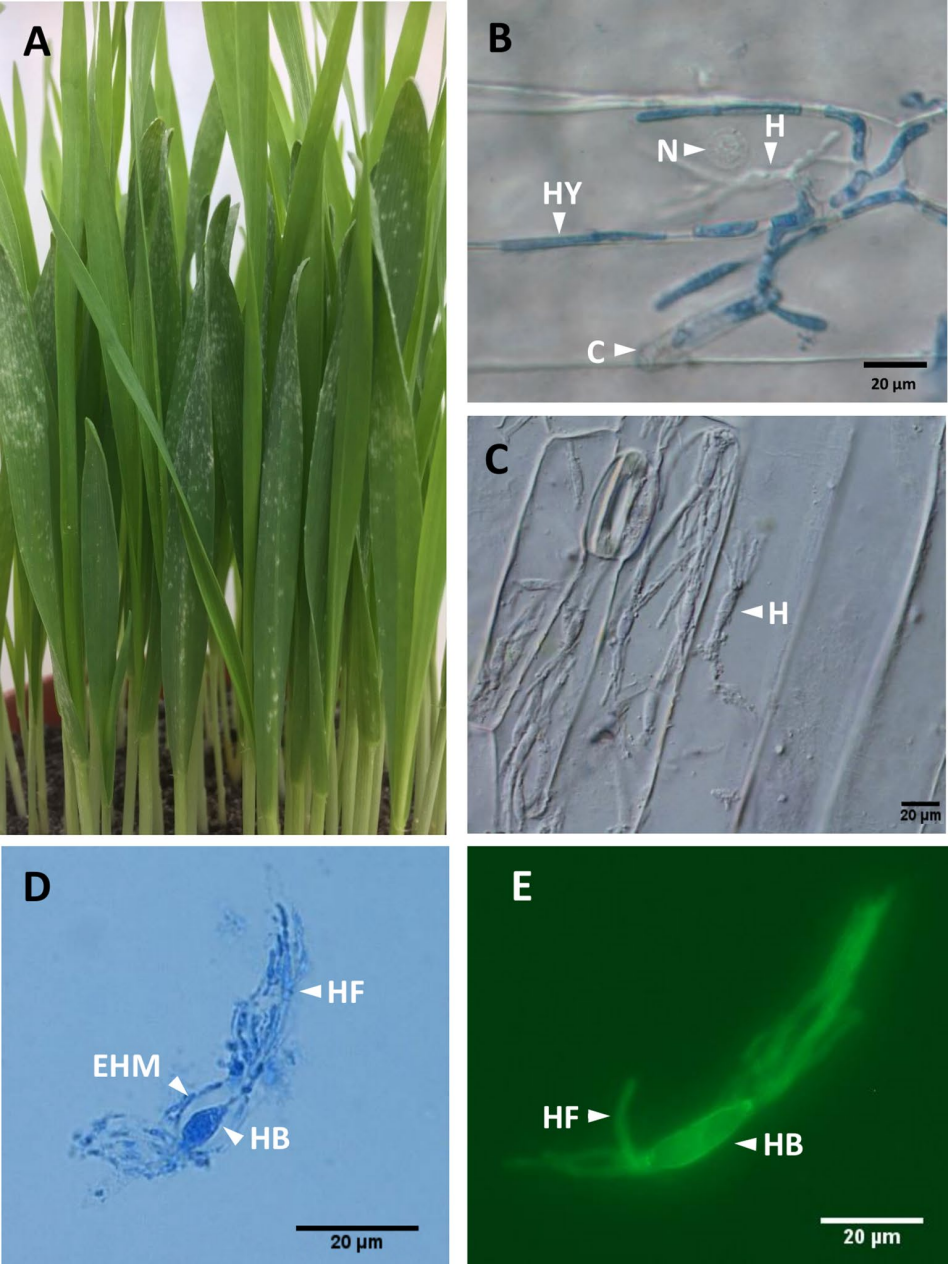
*Blumeria graminis* f. sp. *hordei* (*Bgh*) infection structures. **(A)**, Barley powdery mildew infected plants at 5–6 dpi, showing emergence of white pustules as disease symptoms. At this stage, the leaves are green and turgescent. **(B)**, *Bgh* infection structures at 2 dpi showing conidia **(C)**, hyphae (HY), haustorium (H), and barley nucleus (N). In leaves stained with lactophenol blue, **(C)**, Barley epidermal strips from heavily infected plants, after removal of *Bgh* hyphae. Several haustoria (H) within a single barley epidermal cell can be observed. DIC light microscopy. **(D)**, *Bgh* haustorium isolated from infected barley epidermis, stained with lactophenol blue, showing the haustorial body (HB) and haustorial fingers (HF) surrounded by an extrahaustorial material (EHM). DIC light microscopy. **(E)**, *Bgh* haustorium isolated from infected barley epidermis, labelled with the fluorescent wheat germ agglutinin WGA-AF 488 showing haustorial body (HB) and haustorial fingers (HF), visualized using DAPI suitable conditions. DIC, differential interference contrast.

Powdery mildews cause diseases in numerous plant hosts, including major crops such as cereals, grapevine, strawberries, or cucurbits, to mention a few (Takamatsu, 2013). Therefore, powdery mildews have been considered amongst the top 10 plant fungal pathogens (Dean et al., 2012). Powdery mildews are filamentous ascomycetes belonging to the group Erysiphales. Disease symptoms are easily recognizable by the presence of white powdery pustules on leaf surfaces (**Figure 1A**). As obligate biotrophs, powdery mildews are only able to grow on living plants and typically exhibit a narrow host range (Takamatsu, 2013). Barley and wheat are hosts to *Blumeria graminis* f. sp. *hordei* (*Bgh*) and *B. graminis* f. sp. *tritici* (*Bgt*), respectively. *Blumeria* and *Golovinomyces* *orontii* (*Go*), which is able to infect *Arabidopsis*, are the most studied plant-powdery mildew pathosystems (Bindschedler et al., 2016; Kuhn et al., 2016; Bourras et al., 2018). Moreover, the availability of the barley and *Bgh* genomes allows for large-scale omics analysis and functional genomics studies (Spanu et al., 2010; Mascher et al., 2017; Frantzeskakis et al., 2018).

Powdery mildews exclusively colonize the epidermal layer of leaf tissues, and like other biotrophic pathogens, they form haustoria, the only fungal structures developing within the host tissues (**Figure 1C**). For disease establishment, airborne *Blumeria* conidiospores land on a leaf surface and produce a primary germ tube within 30 min. This is followed by the emergence of a secondary germ tube, which swells into an appressorium within 8 h. In turn, the appressorium produces a penetration peg, which breaks through the subjacent cell wall and forms the first haustorium within 24 h, without disrupting the plasma membrane of the invaded epidermal cell (Both et al., 2005a). When the haustorium has developed successfully, a secondary epiphytic hypha emerges from the appressorium (**Figure 1B**) (Both et al., 2005a).

Powdery mildew haustoria are composed of a fungal core (the haustorial body), delimited by the fungal haustorial plasma membrane and cell wall (**Figures 1D, E**). The haustorial structure is surrounded by the extrahaustorial complex, of which the extrahaustorial membrane (EHM; **Figure 1C**) is of plant origin, newly formed during the haustorium development (Kunoh and Akai, 1969, Micali et al., 2011). The extrahaustorial matrix (EHMx) lies between the fungal cell wall and the EHM, as a sealed compartment.

A major trophic role has been attributed to haustoria as they are responsible for the uptake of nutrients from the host. To do this, ATPases localized to the fungal haustoria generate a proton gradient within the EHMx, to permit H+ symport of hexoses and amino acids (Szabo and Bushnell, 2001).

As a line of defense also occurring during compatible interactions, encasements are formed around haustoria to block further progression of the invading pathogen in *Arabidopsis* (Micali et al., 2011). These encasements are associated with callose deposition and depend on an active callose synthase encoded by the *Powdery Mildew Resistance 4* (*PMR4*) glucan synthase-like 5 (*GSL5*) gene in *Arabidopsis* and *HvGSL6* in barley (Ellinger et al., 2013; Chowdhury et al., 2016). Different biological functions being attributed to the EHM and its isolation from the plant plasma membrane *via* a haustorial neckband (Szabo and Bushnell, 2001) suggest that the EHM differs markedly from the host plasma membrane (Faulkner, 2015). For instance, it was shown that the EHM has properties more similar to the ER (Kwaaitaal et al., 2017). Moreover, several proteins were shown to be differently localized to the EHM while absent from the plasma membrane of the host epidermis. Such is the case for a remorin, exclusively located in specific lipid raft microdomains of the EHM, involved in *Nicotiana benthamiana* susceptibility to the oomycete *Phytophthora infestans* (Bozkurt et al., 2014). Similarly, the plasmodesmatal *Arabidopsis* protein PDLP1 is relocated to haustoria in *Hyaloperonospora arabidopsidis*-infected cells (Caillaud et al., 2014). PDLP1 was shown to promote resistance to downy mildew by inducing callose deposition during haustoria encasement formation (Caillaud et al., 2014). In *Arabidopsis*, the Brassica-specific Resistance to powdery mildew 8 (RPW8.2) protein is implicated in host resistance and is transported to the haustoria *via* VAMP721/722 vesicles (Kim et al., 2014).

In addition to their crucial role in nutrient uptake, haustoria deliver virulence factors (effectors) to the colonized host cells to jeopardize plant immunity or to mask the pathogen intrusion. In the case of *B. graminis*, over 500 candidate effector proteins (CSEPs) have been identified as small secreted proteins, with sequence specificity restricted to the Erysiphales clade (Pedersen et al., 2012). Some of these CSEPs, identified as AVR_a1_ and AVR_a13_, were shown to promote effector-triggered immunity (ETI) in the presence of the corresponding barley R genes *Mla1* and *Mla13* (Lu et al., 2016). While effectors can trigger ETI in a gene-for-gene fashion, many *Bgh* CSEPs have been validated as virulence factors during infection of a susceptible barley cultivar (Zhang et al., 2012; Pliego et al., 2013; Ahmed et al., 2015; Aguilar et al., 2016; Ahmed et al., 2016). Some of these effectors were shown to interact with host proteins, including CSEP0055, which interacts with a pathogenesis-related (PR) protein PR17c, involved in penetration resistance (Zhang et al., 2012), or CSEP0162 and CSEP0105, both of which interact and compromise the activity of small HSP proteins, HSP16.9 and HSP17.5 (Ahmed et al., 2015). In another study, *Bgh* CSEP0064 (also called BEC1054) was shown to interact with several barley proteins, including a malate dehydrogenase, a glutathione-*S*-transferase (GST), an elongation factor (eEFg), and a PR5 thaumatin-like protein (TLP; Pennington et al., 2016). Historically, PR proteins have always been associated with resistance, as they accumulate faster and in more abundance in resistant hosts. However, the precise molecular role in resistance or susceptibility for these interactors has not yet been elucidated. In an attempt to address this, two large-scale protein–protein interaction studies have been undertaken to identify *Arabidopsis* proteins targeted by biotrophic pathogens’ effectors for three taxonomically unrelated organisms: a bacterium, an oomycete, and the *G. orontii* powdery mildew fungus. Surprisingly, effectors tended to directly interact with conserved or universal host proteins with vital functions, rather than species/cultivar-specific Resistance “R” proteins such as NLS-LRR genes. Moreover, it was shown that many of the effector targets were common to the three pathogens (Mukhtar et al., 2011; Weßling et al., 2014). Therefore, the interaction of *Bgh* effectors with PR proteins or proteins involved in primary metabolism is not an oddity.

Although transcriptomes and proteomes of haustoria have been investigated for *Blumeria* (Both et al., 2005a; Both et al., 2005b; Bindschedler et al., 2009, Bindschedler et al., 2011; Godfrey et al., 2009; as reviewed in Bindschedler et al., 2016), sparse or no information is available about the host epidermis and extrahaustorial proteomes. Therefore, the epidermis proteome of a susceptible barley cultivar infected with *B. graminis* f. sp. *hordei* (*Bgh*), was compared with its non-infected counterpart. In a second comparison, the extrahaustorial barley proteome was analyzed in a haustorial fraction enriched from infected epidermis and compared with the proteome of infected epidermis, in an attempt to unravel putative components of resistance and susceptibility. The proteomics analysis was coupled to a transient gene silencing assay to query the role of the PR protein 5 (PR5) isoform TLP5, which was more abundant in infected epidermis and was previously shown to interact with the effector BEC1054 (CSEP0064). Surprisingly, results suggested that TLP5 is a negative modulator of resistance during the barley powdery mildew interaction.

## Materials And Methods

### Plant and Fungus Maintenance

Barley [*Hordeum vulgare* (*Hv*)] cultivar Golden Promise, susceptible to *Bgh* isolate DH14, was grown at 22°C under a 16-h photoperiod by sowing 50 seeds in 13-cm-diameter pots containing John Innes No. 1 compost. *Bgh* isolate DH14 was maintained by weekly transfer of high-density spore inoculum to 7-day-old barley seedlings.

### Epidermis and Haustorium Sample Preparation

**Figure 2:**
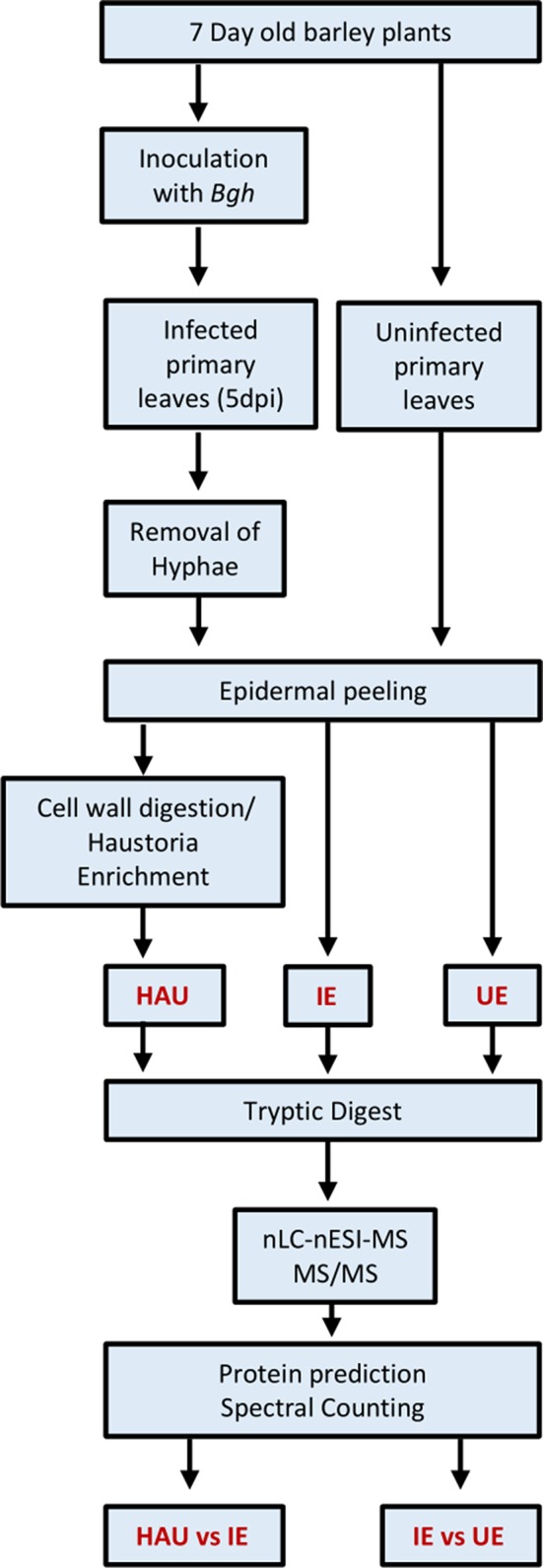
Workflow used to prepare the three types of barley protein samples for proteomics investigation: uninfected epidermis (UE, four biological replicates), infected epidermis (IE, four biological replicates), and enriched haustoria from infected epidermis (HAU, three biological replicates). Powdery mildew infected barley leaves were collected at 5 dpi.

Three different types of samples were prepared: a haustoria-enriched fraction from infected epidermis (HAU; three biological replicates, rep 1–3), infected epidermis (IE; four biological replicates rep 1–4), and uninfected epidermis control (UE; four biological replicates, rep 1–4), according to the workflow summarized in **Figure 2**.

For the IE samples, two pots with about 100 seven-day-old barley plants were inoculated with *Bgh* spores. At 5 days postinoculation (5 dpi), excised primary leaves were individually dipped into 5% cellulose acetate in acetone. Once dried, the film of cellulose acetate containing the epiphytic fungal material was removed (Godfrey et al., 2009). Immediately after this, epidermal strips were peeled from the abaxial surface as detailed previously (Shen et al., 2015), flash frozen, and stored at −80°C until further use. The same procedure was used to harvest uninfected epidermis (UE samples).

For the haustoria-enriched samples (HAU), the infected epidermal strips were not frozen but immediately transferred to a petri dish containing 10 ml of 10 mM MOPS, pH 7.2 buffer, at room temperature. Once 100 leaves’ worth of epidermis samples were harvested, cellulase (Onozuka R-10, Melford #C8001) and Macerozyme (R-10, Melford #M8002) were added to the buffer to a final concentration of 0.1% w/v for each enzyme. Epidermis samples were digested for 2 h at room temperature on a rotating platform. The digest was filtered through a 70-µm nylon mesh sieve, and the petri dishes were rinsed with an additional 10 ml of buffer, before epidermis remains were gently squeezed with a pipette tip and all liquids were recovered and filtered. The filtrate was centrifuged at 400 × *g* for 10 min. All but 1 ml of the supernatant was removed. The haustorial pellet was resuspended. A small aliquot (20 µL) was diluted 1:1 in 2× isolation buffer to obtain a final concentration of 0.2 M of sucrose, 10 mM of MOPS, 1 mM of KCl, 1 mM of MgCl_2_, and 1 mM of CaCl_2_, pH 7.2. From this, 10 µL was mixed with 10 µL of lactophenol blue stain (Pro-Lab Diagnostics #P/L740/08) to estimate the quantity and visualize the quality of the haustorial preparation using a Nikon Ni-Eclipse upright microscope at ×1,000 magnification with differential interference contrast (DIC), under oil immersion. Proteins were precipitated by addition of 10 ml cold 10% trichloroacetic acid (TCA) in acetone to the haustorial suspension and vortexed vigorously. The mixture was stored for 20 min at −20°C for protein precipitation.

### Protein Extraction and Tryptic Digestion

Epidermis samples (IE and UE) were kept frozen while ground to a fine paste in the presence of liquid nitrogen and a small amount of quartz sand in a precooled mortar.

For each samples (HAU, IE, or UE), proteins were extracted and precipitated in 1:10 v/v TCA–acetone as described previously (Bindschedler et al., 2009), but protein pellets were resuspended in a urea resuspension buffer containing 7 M of urea, 20 mM of DTT, and 20 mM of Tris, pH 7.2. Protein concentrations were estimated with a Bradford essay (Bio-Rad, #5000006).

Proteins were digested with trypsin in solution as adapted from (Bindschedler et al., 2011). Proteins (10 µg/10 µL) were prepared in 7 M of urea, 50 mM of ammonium bicarbonate (ABC), and 10 mM of DTT. Proteins were denatured at 42°C for 30 min prior to addition of 5 µL of iodoacetamide in 50 mM of ABC for 10 mg/ml of final concentration. After 30-min incubation in the dark at room temperature, 2.5 µL of 100 mM DTT was added to quench the iodoacetamide, and 50 mM of ABC was added to reduce the final urea concentration to below 1.5 M. Proteins were digested with 200-ng sequencing grade trypsin (Sigma #T6567) and incubated at 37°C for 2 h. An additional 200 ng of trypsin was added, followed by another 2 h incubation. The reaction was stopped by addition of 2 µL of 10% trifluoroacetic acid (TFA). Samples were then cleaned using C18 Ziptips (Millipore, Z720070), as per the manufacturer’s instructions.

### Nano-Liquid Chromatography–Electrospray Ionization–Tandem Mass Spectrometry (nLC-nESI-MS/MS) Analysis

Tryptic peptides were trapped and separated by reversed-phase chromatography on an Ultimate 3000 RSLC nano system (Thermo Fisher Scientific, Dionex, Harpenden, UK) equipped with an Acclaim PepMap µ-precolumn cartridge 300 µm i.d. × 5 mm, 5 μm, 100 Å, and an Acclaim PepMap RSLC 75 µm × 25 cm, 2 µm, 100 Å analytical column (Thermo Fisher Scientific). Peptides in 2% aqueous acetonitrile containing 0.1% TFA were loaded, and the precolumn was washed in the same buffer for 8 min at 10 µL/min. Following transfer to the analytical column, peptides were separated and eluted at 250 nL/min in a 3–25% 140-min acetonitrile gradient and 25–35% 23-min gradient containing 0.1% formic acid (FA). Columns were then reconditioned in 90% acetonitrile, 0.1% FA over 3 min, followed by a 15-min re-equilibration at 3% acetonitrile and 0.1% FA. Eluting peptides were subjected to nano-ESI and analyzed on a Thermo Orbitrap Fusion (Q-OT-qIT, Thermo Fisher Scientific). Survey scans of peptide precursors from 375 to 1,575 *m*/*z* were performed at 120K resolution (at 200 *m*/*z*) with automatic gain control (AGC) 2 × 10^5^. Precursor ions with charge state of 2–7 were isolated (1.6 *m*/*z* isolation window in the quadrupole) and subjected to higher-energy collisional dissociation (HCD) fragmentation with normalized collision energy of 33. The MS scan speed was set to rapid, the AGC was set to 5 × 10^3^, and the max injection time was 200 ms. Dynamic exclusion duration was set to 50 s with a 10-ppm tolerance around the selected precursor and its isotopes. Monoisotopic precursor selection was turned on. The instrument was run in top speed mode with 2-s cycles.

### Database Search for Protein Identification

*.RAW files with MS/MS outputs were converted to *.mgf files and submitted to an in-house Mascot search engine version 2.6.2 (Matrix Science)^1^. Each dataset was searched against both databases of barley 2017 version^2^ (Mascher et al., 2017) and *Bgh* isolate DH14 version 4 (Frantzeskakis and Panstruga, personnel communication, Frantzeskakis et al., 2018). Although only barley proteins are described, both databases were searched to reflect the real search space, with a total of 46,852 sequences and 16,174,129 residues. The Mascot search parameters selected were as follows: 15-ppm tolerance in MS mode and 0.8-Da tolerance in MS/MS mode, carbamidomethyl as fixed modification for cysteine, oxidation for methionine and proline as variable modifications, and two trypsin miss-cleavage allowance, using the Trypsin/P rule. The Mascot *.DAT result files were retrieved as *.CSV files for further processing in Excel using a previously published macro (Pennington et al., 2016). Proteins with same set or subset of peptides were included for the output. Proteins were validated if identified with at least two significant peptides, setting peptide thresholds to P 0.05 confidence with Percolator score above 13, while the false discovery rate was always below 1%.

### Protein Quantitation

A semi-quantitative approach known as spectral counting was performed to identify proteins with differential abundances in infected epidermis relative to uninfected epidermis (IE vs UE) and in haustoria-enriched samples relative to infected epidermis (HAU vs IE). “Spectral count” (SC) values were obtained from the “protein match significant” column of the Mascot output file, that is, the number of times significant peptides (with a significant confidence value) were identified for a given protein. For each biological replicate, in which a protein was not identified with at least two unique and significant peptides, an SC value of 0 was given. Then, for each replicate, the SC values were normalized (SC_norm_) for each protein, as described previously (Lundgren et al., 2010) by dividing SC by the SC sum of all identified and validated proteins for that biological replicate and then multiplied by 1,000. The SC_norm_ averages were calculated for each experimental set: HAU (three biological replicates) and IE and UE (both with four biological replicates). SC_norm_ averages were ratioed to calculate the respective relative abundances HAU/IE and IE/UE. Change in abundance was expressed as the log_2_ HAU/IE and log_2_ IE/UE. A two-tailed homoscedastic *t*-test was performed to estimate differentially abundant proteins (DAPs) between the two samples compared, considering significance for *P* values <0.1 (*), <0.05 *, <0.01 **, and <0.001***. Adapting criteria from Gokce et al. (2011), we identified proteins considered as more abundant in HAU compared with IE in at least three HAU replicates, with log_2_ HAU/IE ratio above 1.585 (corresponding to more than a three-fold increase in relative abundance) for *P* < 0.1 or log_2_ HAU/IE ratio above 1 (more than two-fold increase, for *P* < 0.05). The same rule was applied for the IE/UE comparison. Proteins present in only one type of sample and in at least two biological replicates were qualified as exclusive to that set (denoted as IE, UE, or HAU).

### Gene Ontology and Cellular Localization Analysis

Lists of DAPs were generated from the IE/UE and HAU/IE comparisons. Gene ontology (GO) terms overrepresented in these lists were retrieved using the g:Profiler web tool^3^ (Reimand et al., 2016). The significance threshold was determined by the g:SCS algorithm, which accounts for multiple dependent testing bias. Parameters were as follows: Sort_by_structure = 1, user_ hr = 1.00, organism = Hvulgare, analytical = 1, significant = 1, and version: r1760_e93_eg40.

Cellular localization predictions were obtained with WolfpSort^4^ selecting the “plant organism” setting. Transmembrane domains were predicted with TMHMM v.2.0^5^ and DeepLoc-1.0^6^ using BLOSUM62. Occurrence of glycosylphosphatidylinositol (GPI) anchor motifs was predicted with PredGPI using the general model with an upper confidence threshold set to 99%^7^. The presence of an N-terminal signal peptide for protein secretion was estimated with Signal P 5.0^8^.

### Transient-Induced Gene Silencing (TIGS) Workflow for Functional Genomics

The TIGS workflow was adapted from Sun et al. (2005), Sun et al. (2007), and Dinç et al. (2011). The CDS sequence of the barley *PR5* isoform known to interact with the BEC1054/CSEP0064 effector (Pennington et al., 2016; GenBank ID KP293850, corresponding to UniProt ID O23997) was retrieved from the National Center for Biotechnology Information (NCBI) website^9^.

Gene-specific 19-mer silencing antisense phosphorothioate-modified oligodeoxyribonucleotides (PTOs) were designed with the OligoWalk software^10^ (Lu and Mathews, 2008). Two PTO-predicted sequences with high probability of causing silencing (PTO PR5.1: TTGAAGAACATTGAGTAGT; PTO PR5.2: AAGAAGTCCTTGTTCGCGC; **Supplementary Material 3**) were selected and subjected to Basic Local Alignment Search Tool (BLAST) search against the barley and *Blumeria* genomes to detect any silencing off-targets. PTOZ (AAGCGGTTGAGCACTGAA), targeting the protein Z only expressed in seeds (GenBank ID X97636.1; UniProt ID P06293), was used as the non-target negative control (Sun et al., 2005), in addition to a water treatment control, and a “no silencing treatment” control. Moreover, to validate the methodology in this experimental context, a positive control treatment, targeting the well-characterized susceptibility gene *HvMLO1*, was included for silencing (PTO MLO1 TAGTCAACGTACTTGCTGG). The PTOs purchased from Sigma (Sigma Aldrich, Gillingham, UK) were reconstituted as 100 µM stocks in sterile dH_2_O and stored at −20°C until further use.

For the silencing treatments, excised 7-day-old barley primary leaves were cut under water (to prevent blockage of vascular tissue) with a sharp razor blade to 8 cm from the tip. Three leaves were then transferred to 2-ml microfuge tubes containing 1 ml of 10 µM PTO solution. The tubes were incubated under continuous light for 24 h in a partially closed transparent Plexiglas chamber to encourage leaf transpiration and allow for PTO uptake *via* the vascular tissues. Then, the submerged region of the leaf was removed by cutting off 2 cm at the base of the leaf. Leaves were immediately plated on 12 cm square petri dishes containing 50 ml of 0.6% agar supplemented with 20 mg/L benzimidazole. The leaves were flattened by placing glass Pasteur pipettes on each end. A pot of 7- to 8-dpi powdery mildew infected barley was shaken 24 h before inoculation to remove old spores and have a uniform spore population. This pot was then placed in an infection cabinet. Petri dishes containing the PTO-treated leaves were transferred to the inoculation cabinet after the lid was removed. Fungal inoculation was achieved by creating a spore cloud using a hair dryer to blow cold air on the infected barley leaves from the inoculation pot through small holes in the incubation chamber. A hemocytometer was also included to estimate the spore density, usually 30 to 60 spores/mm^2^. Spores were left to settle for 20 min before closing the petri dishes and incubating them under a 16-h photoperiod and low-light conditions to favor *Bgh* infection. For consistency of the infection, fungal inoculation was always performed towards the end of the photoperiod.

### Microscopy Observations and Disease Scoring in Blumeria-Infected Leaves

At 44 hpi, PTO-treated and infected barley leaves were cut at 2.5 cm from the tip (“tip”) and 2.5–4.5 cm from the tip (“base”) segments and immediately transferred into 2-mL microfuge tubes containing lactophenol blue (Pro-Lab Diagnostics #P/L740/08) before incubation at 90°C for 20 min to permit staining. Leaves were destained twice in 3:1 ethanol:acetic acid for 20 min and overnight, respectively, prior to storage in 20% glycerol at 4°C until microscopic observation. Disease scoring was performed by mounting the leaves in 20% glycerol and monitoring infection events under 250× or 400× magnification. At least 400 spores per leaf segments were observed, recording non-germinated conidia (NG), conidia with appressoria (AP), or conidia with secondary hyphae (SH), with the proportion of SH (%SH) reflecting successful infection. For PR5 gene silencing experiments and the corresponding PTOZ controls, a total of 43 individual leaves were analyzed for each treatment, spread across seven independent biological replicates. In the case of MLO gene silencing and the corresponding PTOZ controls, a total of 19 individual leaves were analyzed for each treatment, spanning across four independent biological replicates. Generalized linear mixed models (GLMMs) were calculated with the R software^11^ to estimate the statistical significance.

For detection and imaging of fungal specific structures *in planta*, staining was performed as described above with lactophenol blue (Pro-Lab Diagnostics). For the detection of haustoria in epidermal strips, these were directly mounted in 20% glycerol and observed by DIC light microscopy. For isolated haustoria preparations, samples were briefly incubated with lactophenol blue: 2× haustorium isolation buffer at room temperature in 1:1 volume prior mounting of the specimen on glass slide in 20% glycerol for observation. Alternatively, isolated haustoria were stained in 1× haustoria isolation buffer containing 10 μg/ml wheat germ agglutinin lectin *conjugated to* Alexa Fluor 488 (WGA-AF 488; #W11261 Thermo Fisher Scientific). These samples were observed by fluorescence microscopy, using an EVOS™ Light Cube filter system with excitation/emission wavelengths at 470/20 and 510/42 nm, respectively, in pre-set conditions suitable for green fluorescent protein (GFP) detection.

### RNA Extraction and Quantitative Reverse Transcription Polymerase Chain Reaction (qRT-PCR) to Estimate mRNA Expression Levels

For each sample, total RNA was extracted from a pool of six leaves using a RNeasy Plant Mini kit (QIAGEN, Manchester, UK). RNA concentration and purity were checked with a NanoDrop1000 spectrophotometer (Thermo Fisher Scientific, Hemel Hempstead, UK). cDNA was synthesized from 1 µg of RNA using the Quantiscript Reverse Transcription kit (QIAGEN) according to the manufacturer’s protocol, including a gDNA removal step.

The qPCR was set up using PrecisionPlus qPCR master mix (PrimerDesign, Southampton, UK) containing SYBR Green dye. The qRT-PCRs were performed in a Rotor-Gene Q real-time PCR cycler (QIAGEN) and included an initial step at 95°C for 2 min prior to 40 cycles (14 s at 95°C, 60 s at 60°C). Melt curves were acquired to check primer specificity. Ct values were determined using the Rotor-Gene Q software version 2.3 (QIAGEN). The relative transcript level of the targeted gene was determined according to Pfaffl (2001) using barley *GAPDH* (GenBank ID X60343) as the reference gene. Each biological replicate included a pool of six leaves and was analyzed in three technical replicates. *TLP5* expression level in the time course experiment was performed across three biological replicates. For the estimation of gene silencing impact on the targeted gene, expression of *HvMLO1* and *HvTLP5* was performed as three and six independent biological replicates, respectively. One-sample *t*-test was performed to determine the statistical significance.

### DAB Staining for Detection of H_2_O_2_ in Barley Infected Cells

Hydrogen peroxide was detected by 3,3′-diaminobenzidine (DAB; Sigma Aldrich) staining of *Bgh*-infected barley leaves, harvesting the 2.5-cm “tip” segments at 18 hpi, adapting the processes from previous protocols (Bindschedler et al., 2006; Daudi and O’Brien, 2012). The solution was prepared freshly by solubilizing 1 mg/mL DAB in dH_2_O acidified with 1:10 volume of 0.1 M of HCl to lower the pH to 3.8 and shaking vigorously for 2 h in the dark. Sodium phosphate (pH 7) to a final concentration of 10 mM and Tween 20 to a final concentration of 0.05% were added just before use. The freshly excised 2.5-cm leaf tips were vacuum infiltrated with the DAB solution. The infiltrated leaves were then incubated on damp filter paper under low light for 4 h to enable DAB precipitation in the presence of H_2_O_2_. The leaves were finally stained with lactophenol blue and destained as described above. H_2_O_2_-producing barley cells were observed by microscopy at 400× magnification as light to dark brown-stained cells. At least 100 germinated spores per leaf were observed to monitor the percent germinated spores associated with DAB-stained cells, in a total of 18 leaves (across three biological replicates) for each PTO gene silencing and control treatments. A one-way analysis of variance (ANOVA) test and post-hoc Tukey honestly significant difference (HSD) was performed to determine the statistical significance.

## Results

### Proteomics Workflow to Identify Proteins Co-Localized With Infected Cells and Haustorial Structures

Three different types of sample sets were prepared from isolated epidermis of infected or uninfected barley primary leaves, collected at 5 dpi, following infection with barley powdery mildew, as summarized in **Figure 2**. The three sample sets were as follows: UE, uninfected epidermis (four biological replicates); IE, infected epidermis (four biological replicates); and HAU, haustoria-enriched fraction prepared from infected epidermis (three biological replicates). A 5-dpi time point was chosen, since at this stage, the haustoria density was the highest, before any signs of chlorosis were visible (**Figure 1A**). Also, following heavy infection with *Bgh* spores, the fungus was well established, with many haustoria packed in a large proportion of epidermal cells. This ensured enough fungal biomass and a sufficiently large number of haustoria to allow for a successful proteomics analysis (**Figure 1C**). The HAU samples were obtained from digesting IE samples with cell-wall-digesting enzymes. Haustoria were released by disruption of the plant epidermis plasma membrane and cytolysis while keeping intact haustoria. Haustorial structures were recovered and enriched by filtration and differential centrifugation (**Figures 1D, E**). Haustoria were surrounded by a perihaustorial structure, which is presumed to include the plant EHM (**Figure 1D**) and matrix. Haustoria-enrichment steps were kept to a minimum to avoid damaging the fragile structures, since it was observed that additional steps led to a drastic decrease of intact haustoria.

**Figure 3:**
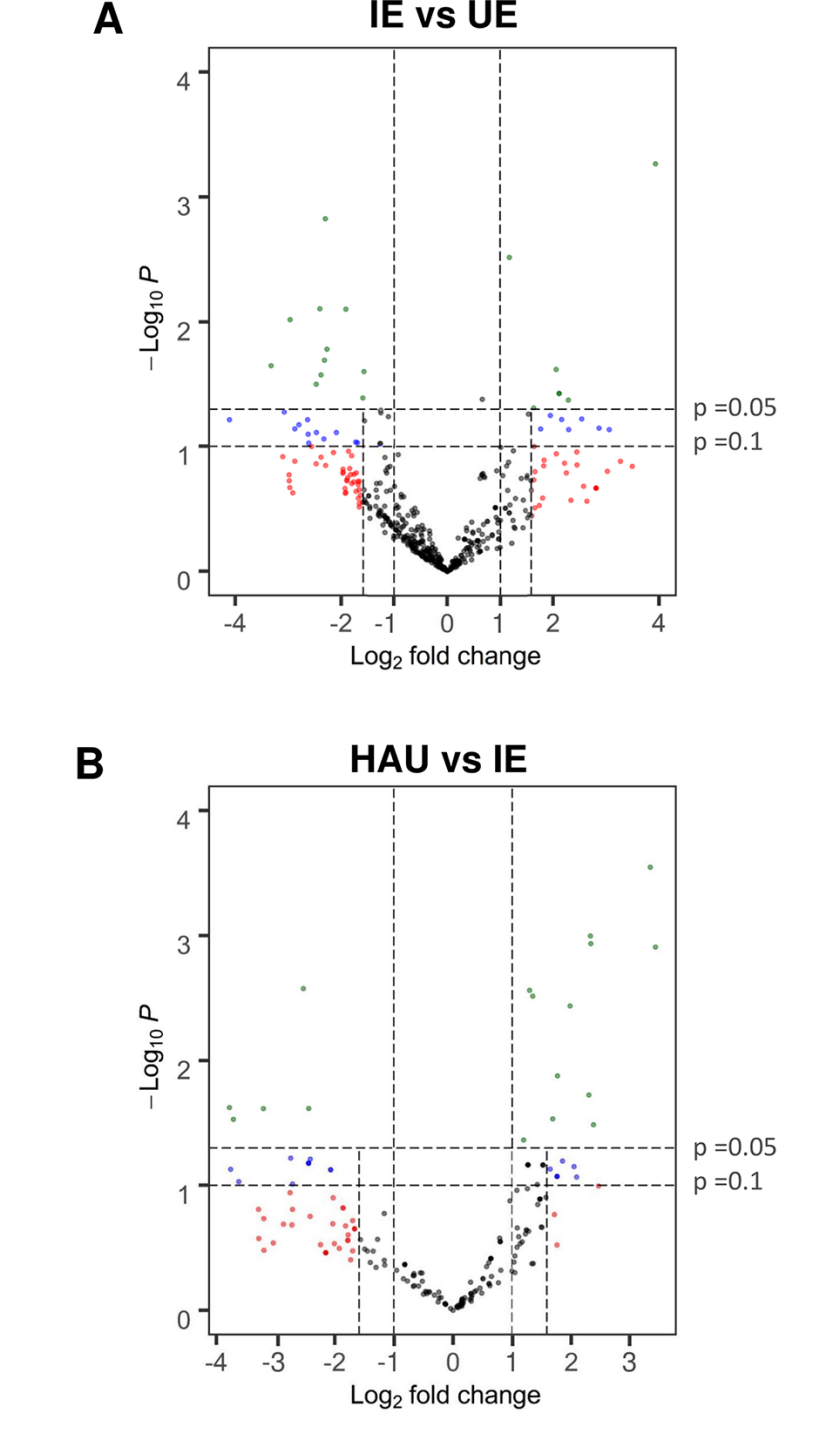
Volcano plot showing relative protein abundance calculated as ratios of normalized spectral count for proteins identified in both **(A)**, IE and UE, and **(B)**, HAU and IE; with identification in at least three replicates of either set. A *t*-test was performed on normalized spectral count values for each protein comparison. Proteins with a *P* value ≤0.1 and a log_2_ fold change ≥1.585 (more than three-fold change), and proteins with a *P* value ≤0.05 and a log_2_ fold change ≥1 (more than two-fold change) were considered differentially abundant. Proteins that were exclusive to either IE, UE, or HAU are not included. IE, infected epidermis; UE, uninfected epidermis; HAU, haustoria-enriched fraction.

For the following label-free differential proteomics analysis, protein extracted with TCA-acetone was digested with trypsin and subjected to nLC–nESI–MS/MS for protein identification. Then relative protein abundances were estimated for each identified protein (**Supplementary Material S1**), comparing the ratios of normalized SC (SC_norm_) IE with UE (IE/UE) and HAU with IE (HAU/IE) to identify DAPs. We considered as more abundant DAPs those proteins identified in at least three biological replicates of infected epidermis (IE) or enriched haustoria (HAU) increased by more than three-fold when compared with uninfected epidermis (UE) or infected epidermis (IE) samples, respectively (SC_norm_ log_2_ ratio >1.585) with a *P* value <0.1. For *P* values lower than 0.05, a two-fold threshold was set. These were depicted as green dots on the volcano plots presented (**Figure 3**). Proteins exclusive to IE or HAU, seen at least twice in that set, are also presented (**Tables 1** and **2**). With the use of these criteria, a total of 40 DAPs were “more abundant” in IE when compared with UE, while 29 DAPs were more abundant in HAU relative to IE. Prior to normalization of the SC values (SC_norm_), the average log_2_ values of IE vs UE and HAU vs IE were below 0, indicating a bias towards globally less abundant barley proteins in IE and HAU samples. This may be accounted for by the amount of *Bgh* biomass in IE, but especially in HAU samples. This led to subsequent reduction in the amount of barley proteins identified overall, hence the requirement of normalizing the SC values. Furthermore, proteins in infected samples may have been subjected to more oxidation abuse and post-translational modifications, potentially reducing their extractability or their identification. Consequently, following normalization, a threshold of log_2_ ratio >1 (>2-fold change) was considered to be sufficiently conservative.

Because proteins accumulating in infected epidermis or re-localizing to the plant component of the haustorial complexes might be putative mediators of the barley–*Bgh* interaction, these were scrutinized further.

### Comparison of Epidermis Proteomes From Infected vs Healthy Barley Leaves

A list of DAPs that were more abundant in the infected epidermis (IE vs UE) was generated (**Table 1**). It included many isoforms of classical, predicted to be secreted, PR proteins, such as chitinases, peroxidases, cysteine-rich venom proteins, which contain an N-terminal PR1 motif (Guo et al., 2005), and several polysaccharide hydrolases (endo-1,3-β-glucosidase or PR2 and β-1,3-1,4-glucanase, β-galactosidase, chitinase, or PR3). In particular, two TLPs or PR5 isoforms were identified with an abundance increase of five-fold (SC_norm_ log_2_ ratio 2.30) and 15-fold (SC_norm_ log_2_ ratio 3.94) in the IE. Other likely stress-related proteins that were exclusive to the IE set included heat shock proteins (HSP40 and HSP70), a xylanase inhibitor, and the ethylene biosynthetic enzyme aminocyclopropane carboxylate oxidase. In addition to HSPs with protein folding or chaperone activity, two FKBP-like peptidyl prolyl *cis*–*trans* isomerases, related to cyclophilins, also showed increased abundance in infected epidermis. Noticeably, there were also numerous proteins involved in redox reactions or homeostasis, including thioredoxins, 2-oxoglutarate (2OG), and Fe(II)-dependent oxygenases, as well as peroxidases. Finally, several enzymes involved in primary metabolism were identified as more abundant DAPs, including several different subunits of ATP synthases/ATPases, a cysteine synthase, an adenosylhomocysteinase, and a malate dehydrogenase likely to be involved in the TCA cycle.

**Table 1:** List of differentially abundant proteins (DAPs) with a significant increase in relative abundance in IE when compared with UE (IE vs UE comparison). The number of replications per experimental set (Rep count) and log_2_ fold change (average of spectral count) or exclusivity in one set are reported for both HAU vs IE and IE vs UE comparisons.

		Rep count		IE vs UE	Graphs of normalized Spectral match		Rep count		HAU vs IE
Transcript ID	Description	IE	UE	Log2 fold change	IE	UE	HAU	IE	Log2 fold change
HORVU4Hr1G002650.2	Pathogenesis-related thaumatin superfamily protein^#^	4	1	3.94***	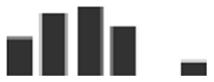		2	4	0.32
HORVU3Hr1G073780.1	Alpha-1,4-glucan-protein synthase (UDP forming)	3	1	3.06(*)	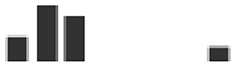		0	3	IE
HORVU3Hr1G105630.7	Glucan endo-1,3-beta-glucosidase^#^	3	3	2.87(*)	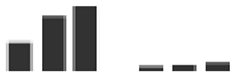		2	3	1.05
HORVU0Hr1G001490.2	Photosystem I reaction center subunit VI, chloroplastic^§^	3	2	2.54(*)	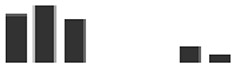		0	3	IE
HORVU5Hr1G051970.4	Pathogenesis-related thaumatin superfamily protein^#^	3	2	2.30(*)	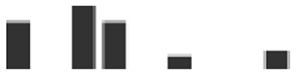		2	3	0.29
HORVU2Hr1G044340.1	Peroxidase superfamily protein^#^	4	3	2.29*	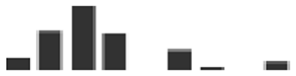		3	4	0.15
HORVU4Hr1G056820.1	Elongation factor 1-alpha	4	1	2.16*	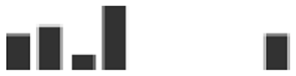		1	4	−2.41
HORVU6Hr1G049300.1 HORVU0Hr1G031480.1	Cytochrome *f* ^§^	4	2	2.12*	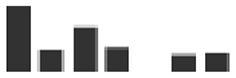		2	4	−0.13
HORVU5Hr1G056040.1	Cysteine-rich venom protein^#^	4	4	2.06*	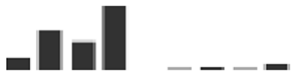		3	4	1.24
HORVU2Hr1G018480.1	Peroxidase superfamily protein^#^	4	3	1.95(*)	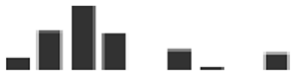		3	4	0.15
HORVU2Hr1G018510.1	Peroxidase superfamily protein^#^	4	3	1.77(*)	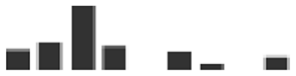		1	4	−2.03
HORVU5Hr1G055950.2	Cysteine-rich venom protein^#^	4	4	1.64*	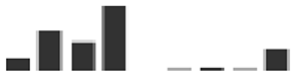		3	4	1.24
HORVU1Hr1G081550.1	Malate dehydrogenase	4	4	1.18**	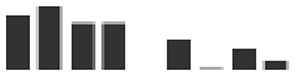		0	4	IE
HORVU3Hr1G052370.1	Mitochondrial ATP synthase subunit G protein	2	0	IE	IE		0	2	IE
HORVU4Hr1G058970.6	ATP synthase subunit b∼^§^	2	0	IE	IE		0	2	IE
HORVU2Hr1G000280.3	Cysteine synthase C1^§^	2	0	IE	IE		1	2	0.71
HORVU2Hr1G109370.1	Adenosylhomocysteinase	2	0	IE	IE		1	2	−2.67
HORVU2Hr1G125110.6	Peroxidase superfamily protein^#^	2	0	IE	IE		0	2	IE
HORVU2Hr1G091130.1	Thioredoxin superfamily protein	2	0	IE	IE		0	2	IE
HORVU2Hr1G091200.3	Thioredoxin superfamily protein	2	0	IE	IE		0	2	IE
HORVU6Hr1G088430.1 HORVU6Hr1G088440.1	2-Oxoglutarate (2OG) and Fe(II)-dependent oxygenase superfamily protein	2	0	IE	IE		0	2	IE
HORVU6Hr1G088420.1	2-Oxoglutarate (2OG) and Fe(II)-dependent oxygenase superfamily protein	3	0	IE	IE		0	3	IE
HORVU1Hr1G062030.1	Chitinase family protein^#^	2	0	IE	IE		0	2	IE
HORVU7Hr1G086690.2	Beta galactosidase 1^§^	2	0	IE	IE		1	2	−1.03
HORVU1Hr1G062080.5	Endo-1,3;1,4-beta-d-glucanase	2	0	IE	IE		0	2	IE
HORVU3Hr1G100350.2	Xylanase inhibitor	2	0	IE	IE		0	2	IE
HORVU0Hr1G009410.1	Germin-like protein 2^#^	2	0	IE	IE		0	2	IE
HORVU3Hr1G099760.1	l-Gulonolactone oxidase 2^#^	2	0	IE	IE		1	2	−2.36
HORVU6Hr1G075080.4	FKBP-like peptidyl-prolyl *cis*–*trans* isomerase family protein	2	0	IE	IE		0	2	IE
HORVU5Hr1G073050.2	FKBP-like peptidyl-prolyl *cis*–*trans* isomerase family protein	2	0	IE	IE		1	2	−1.33
HORVU1Hr1G020450.1	1-Aminocyclopropane-1-carboxylate oxidase	2	0	IE	IE		0	2	IE
HORVU1Hr1G072220.4	Heat shock 70-kDa protein	2	0	IE	IE		1	2	−0.32
HORVU7Hr1G081510.1	DnaJ/Hsp40 cysteine-rich domain superfamily protein	2	0	IE	IE		0	2	IE
HORVU1Hr1G060090.2	Tocopherol cyclase, chloroplastic	2	0	IE	IE		0	2	IE
HORVU5Hr1G064700.7	Ribulose bisphosphate carboxylase large chain	3	0	IE	IE		1	3	−0.67
HORVU7Hr1G040380.5	Chlorophyll *a*/*b*-binding protein, chloroplastic	2	0	IE	IE		1	2	0.89
HORVU1Hr1G088900.2	Chlorophyll *a*/*b*-binding protein, chloroplastic	2	0	IE	IE		1	2	−0.78
HORVU6Hr1G016880.1	Chlorophyll *a*/*b*-binding protein, chloroplastic	2	0	IE	IE		1	2	0.54
HORVU1Hr1G088900.2 HORVU1Hr1G089180.1	Chlorophyll *a*/*b*-binding protein, chloroplastic	2	0	IE	IE		1	2	−0.78
HORVU6Hr1G049190.1	Photosystem I P700 chlorophyll *a* apoprotein A2	2	0	IE	IE		1	2	−0.51

IE, infected epidermis; UE, uninfected epidermis; HAU, haustoria-enriched fraction.

^§^Proteins predicted with transmembrane domains. ^#^Proteins predicted with a signal peptide or extracellular. Significance of differential ratios is shown as ***P < 0.001; **P < 0.01; *P < 0.05; (*) P < 0.1.

A GO profiling analysis was performed to find GO terms that were significantly overrepresented in the infected and uninfected epidermis (IE and UE), as well as GO terms that were overrepresented in the list of DAPs with increased abundance in IE (IE vs UE comparison; **Supplementary Material S2**). Of interest were the biological processes for response to stress, cellular oxidant detoxification, external biotic stimuli, and oxidative stress. Biological processes for ATP synthesis and transport, and hydrolysis, were also overrepresented.

Several chloroplastic proteins were also identified as more abundant in the IE set relative to the UE set, including chlorophyll *a*/*b*-binding proteins, a RuBisCO large subunit protein, and a component of the photosystem I. This might be due to the infected epidermis preparations containing more mesophyllic tissue than the uninfected epidermis. However, there was no significant overrepresentation of GO terms related to “chloroplast” or “photosynthesis” in either IE or UE (**Supplementary Material 2**). Moreover, the relative protein abundance for most of the chloroplastic-associated proteins identified in both tissues remained unchanged (**Supplementary Material 1**).

### Analysis of the Haustoria-Enriched Proteome From Infected Barley Epidermis

A list was generated to identify the DAPs that were more abundant in or exclusive to the enriched haustorial fraction (HAU) when compared with infected epidermis (IE) (HAU vs IE, **Table 2** and **Supplementary Material S1**). Proteins with increased abundance in HAU were typically seen with an unchanged or decreased abundance in the IE vs UE comparison. This suggests that these DAPs are not merely induced by infection or mesophyllic contamination occurring from preparing epidermal strips but are more likely to be relocated to the EHM or organelles trapped within the encasements (Micali et al., 2011). DAPs increased or exclusive to HAU included isoforms of histone H2B (sharing the same peptides for protein identification) but no other histones, suggesting that these DAPs did not originate from an increase of nuclei that might be co-enriched in HAU samples. Similarly, fewer chloroplastic proteins were identified as DAPs with higher abundance in the HAU samples than was the case for the IE samples. In contrast to the IE vs UE comparison, there was no abundance increase for “classical PR proteins” in the HAU sample when compared with the IE sample. This suggests that PR proteins do not preferentially relocate to the haustorial structures, after their induction in infected epidermis. It also confirms that the barley proteome of the enriched haustorial fraction is genuinely different from the barley proteome of infected epidermis.

**Table 2:** List of differentially abundant proteins (DAPs) with a significant increase in relative abundance in HAU when compared with IE (HAU vs IE comparison). The number of replications per experimental set (Rep count) and log_2_ fold change (average of spectral count) or exclusivity in one set are reported, for both HAU vs IE and IE vs UE comparisons.

				IE vs UE			HAU vs IE	Graphs of normalized spectral match	
Transcript ID	Description	IE	UE	Log2 fold change	HAU	IE	Log2 fold change	HAU	IE
HORVU4Hr1G068460.3	Mitochondrial-processing peptidase subunit beta	1	4	−2.40	3	1	3.42**	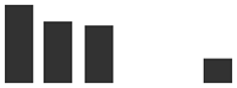	
HORVU3Hr1G067040.5	V-type ATP synthase beta chain	3	3	−1.72	3	3	3.33***	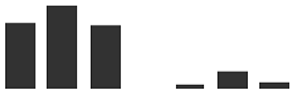	
HORVU6Hr1G049390.3	Photosystem II CP47 reaction center protein^§^	1	3	0.06	3	1	2.37*	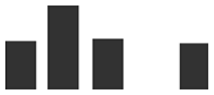	
HORVU6Hr1G039890.3	V-type ATP synthase beta chain	4	4	−1.25	3	4	2.33**	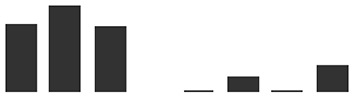	
HORVU7Hr1G087190.1	V-type ATP synthase beta chain	4	4	−1.26	3	4	2.32**	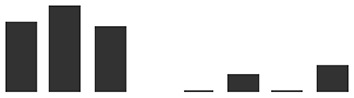	
HORVU7Hr1G109770.1	V-type ATP synthase alpha chain	3	4	−1.07	3	3	2.30*	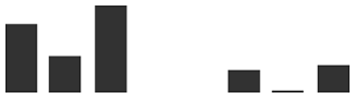	
HORVU6Hr1G070780.1	ADP, ATP carrier protein, mitochondrial^§^	2	4	−0.92	3	2	2.09(*)	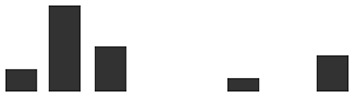	
HORVU1Hr1G046400.1	Non-specific lipid-transfer protein 2^§^	2	3	−0.40	3	2	2.04(*)	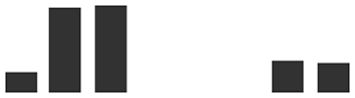	
HORVU0Hr1G019950.1	ATP synthase subunit alpha	3	4	-0.05	3	3	1.98**	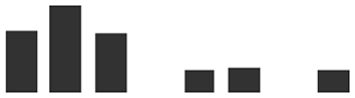	
HORVU7Hr1G058120.1	Chlorophyll *a*/*b*-binding protein 1B, chloroplastic	2	2	0.29	3	2	1.85(*)	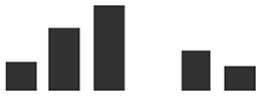	
HORVU1Hr1G083840.2	ATP synthase subunit beta	3	4	−0.44	3	3	1.77*	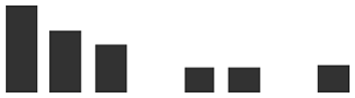	
HORVU1Hr1G088920.3	Chlorophyll *a*/*b*-binding protein, chloroplastic^a^	2	1	2.32	3	2	1.76(*)	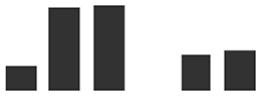	
HORVU4Hr1G080640.1	Aconitate hydratase 1	2	3	−0.06	3	2	1.68*	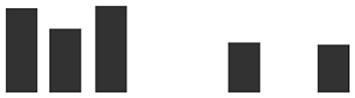	
HORVU5Hr1G102900.1	Cysteine-rich receptor-like protein kinase 25^#^	3	4	−0.16	3	3	1.64 (*)	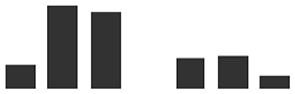	
HORVU3Hr1G019540.2	mitochondrial processing peptidase alpha subunit	4	3	0.33	3	4	1.35**	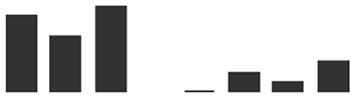	
HORVU2Hr1G079840.1	Lipase/lipooxygenase, PLAT/LH2 family protein	4	4	−0.33	3	4	1.29**	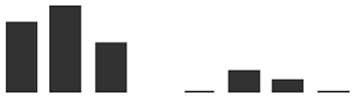	
HORVU6Hr1G008880.1	Heat shock 70 kDa protein C BiP^§#¥^	3	4	0.48	3	3	1.19*	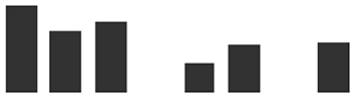	
HORVU7Hr1G104350.1	Early nodulin-like protein 9^†#^	0	3	UE	2	0	HAU	HAU	
HORVU2Hr1G089940.1	Aquaporin-like superfamily^§^	0	2	UE	2	0	HAU	HAU	
HORVU6Hr1G071190.3	Eukaryotic aspartyl protease family protein^#^	0	3	UE	2	0	HAU	HAU	
HORVU4Hr1G026720.5	Alpha-l-arabinofuranosidase 1	0	4	UE	2	0	HAU	HAU	
HORVU6Hr1G003770.1	Dihydrolipoyllysine-residue acetyltransferase component of pyruvate dehydrogenase complex	0	3	UE	2	0	HAU	HAU	
HORVU2Hr1G043960.1	Oxygen-evolving enhancer protein 3-1, chloroplastic	0	1	UE	2	0	HAU	HAU	
HORVU3Hr1G088500.2	Fructose-bisphosphate aldolase 2	0	0	UE	2	0	HAU	HAU	
HORVU3Hr1G086620.1	Histone H2B.5	0	0	UE	2	0	HAU	HAU	
HORVU2Hr1G079550.1	Histone superfamily protein ^b^	0	2	UE	2	0	HAU	HAU	
HORVU5Hr1G066000.1	60S ribosomal protein L34	0	0	UE	2	0	HAU	HAU	
HORVU5Hr1G036630.1	Ribosomal protein 1	0	3	UE	2	0	HAU	HAU	
HORVU4Hr1G019980.1	Ribosomal protein 1	0	1	UE	2	0	HAU	HAU	

IE, infected epidermis; UE, uninfected epidermis; HAU, haustoria-enriched fraction.

Additional proteins that have identical description and log_2_ fold change ratio: (a) HORVU6Hr1G016940.1, HORVU1Hr1G078380.1; (b) HORVU7Hr1G033030.1, HORVU7Hr1G100690.1, HORVU1Hr1G073670.1, HORVU1Hr1G074340.1, HORVU7Hr1G012820.2. † Proteins predicted with a glycosylphosphatidylinositol (GPI)-anchored sequence. ^§^Proteins predicted with transmembrane domains. ^#^Proteins predicted with a signal peptide or extracellular. ^¥^Presence of HDEL motif for ER retention. Significance of differential ratios is shown as *** p < 0.001; ** p < 0.01; * p < 0.05; (*) P < 0.1. Significance of differential ratios is shown as *** p < 0.001; (*) P < 0.1.

A large proportion of DAPs with increased abundance or exclusive to HAU included several subunits (alpha and beta subunits) of V-type ATP synthases (ATPases) as well as an ADP/ATP carrier protein, which, collectively, are associated with the overrepresented GO term for biological function “ATPase movement of ions” (**Supplementary Material 2**). Other membrane-associated proteins identified with increased abundance in HAU included an aquaporin-like protein, predicted with six transmembrane domains and an early nodulin-like protein 9, predicted with a GPI anchoring site. An aconitate hydratase involved in the TCA cycle was also more abundant in HAU-enriched samples (**Table 2**). Several proteases, including a secreted aspartic protease and two mitochondrial processing peptidase subunits (alpha, beta), were also more abundant in HAU. Another DAP of note, more abundant in the HAU samples, was an arabinofuranosidase, and finally, a 70-kDa chaperone, similar to the ER-based binding immunoglobulin protein (BiP).

### PR5 TLP 5 (TLP5) Gene Expression During Infection

The TLP5, encoded by the HORVU5Hr1G051970.4 gene, was shown to be five times more abundant in the infected epidermis when compared with the uninfected counterpart (IE vs UE comparison; **Table 3**). A pairwise alignment between HORVU5Hr1G051970.4 and HORVU5HrG005180.6 showed 93% homology between these two isoforms (**Supplementary Material S3**). Moreover, these two isoforms shared many of the peptides that were identified experimentally and are, therefore, difficult to distinguish (**Supplementary Material S3**). HORVU5HrG005180.6, which originates from the sequencing of the barley cultivar Morex, is 100% identical to the CDS KP293850.1 encoding for the TLP5 protein from the barley cultivar Golden Promise used in this study. TLP5 was previously shown to be a target of the *Bgh* effector BEC1054/CSEP0064, as it directly interacted with this effector as shown in pull-down and yeast two-hybrid experiments (**Supplementary Material S3**; Pennington et al., 2016). Since two TLP isoforms were identified as more abundant in infected epidermis in the IE vs UE comparison and since the *TLP5* CDS sequence was available for the barley cultivar Golden Promise (KP293850.1, 100% homologue to HORVU5HrG005180.6), the role of TLP5 in the compatible barley–*Bgh* interaction was further investigated using a gene silencing approach.

**Table 3:** Thaumatin-like proteins with differential relative abundance in the IE vs UE comparison.

			Rep count		IE vs UE		Rep count		HAU vs IE	
Transcript ID	UniProt ID	PTO match	IE	UE	Log_2_ fold change	P	HAU	IE	Log2 fold change	P
HORVU4Hr1G002650.2	M0W099	NA	4	1	3.94 ***	0.001	2	4	0.32	0.757
HORVU5Hr1G005180.1	A0A287Q7Z4	NA	4	4	0.18	0.835	3	4	-0.04	0.967
HORVU5Hr1G051970.4	A0A287R7D5	PR5.2	3	2	2.30 (*)	0.074	2	3	0.29	0.818
HORVU5Hr1G005180.6/KP293850.1	O23997	PR5.1 PR5.2	Protein interacting with BEC1054/CSEP0064 (Pennington et al., 2016) 90% Protein homology to A0A287R7D5 94% nucleotide identity to HORVU5Hr1GO51970.4 (380/719 NTs)							

IE, infected epidermis; UE, uninfected epidermis; HAU, haustoria-enriched fraction.

For each PR5 isoform described, accession number of transcript ID and UniProt ID are given. PTO match describes PTO sequences that can potentially target the given isoform. The number of replication per experimental set (Rep count) and log_2_ fold change (average of spectral count) are also reported. Significance of differential ratios is shown as *** p < 0.001; (*) P < 0.1.

**Figure 4:**
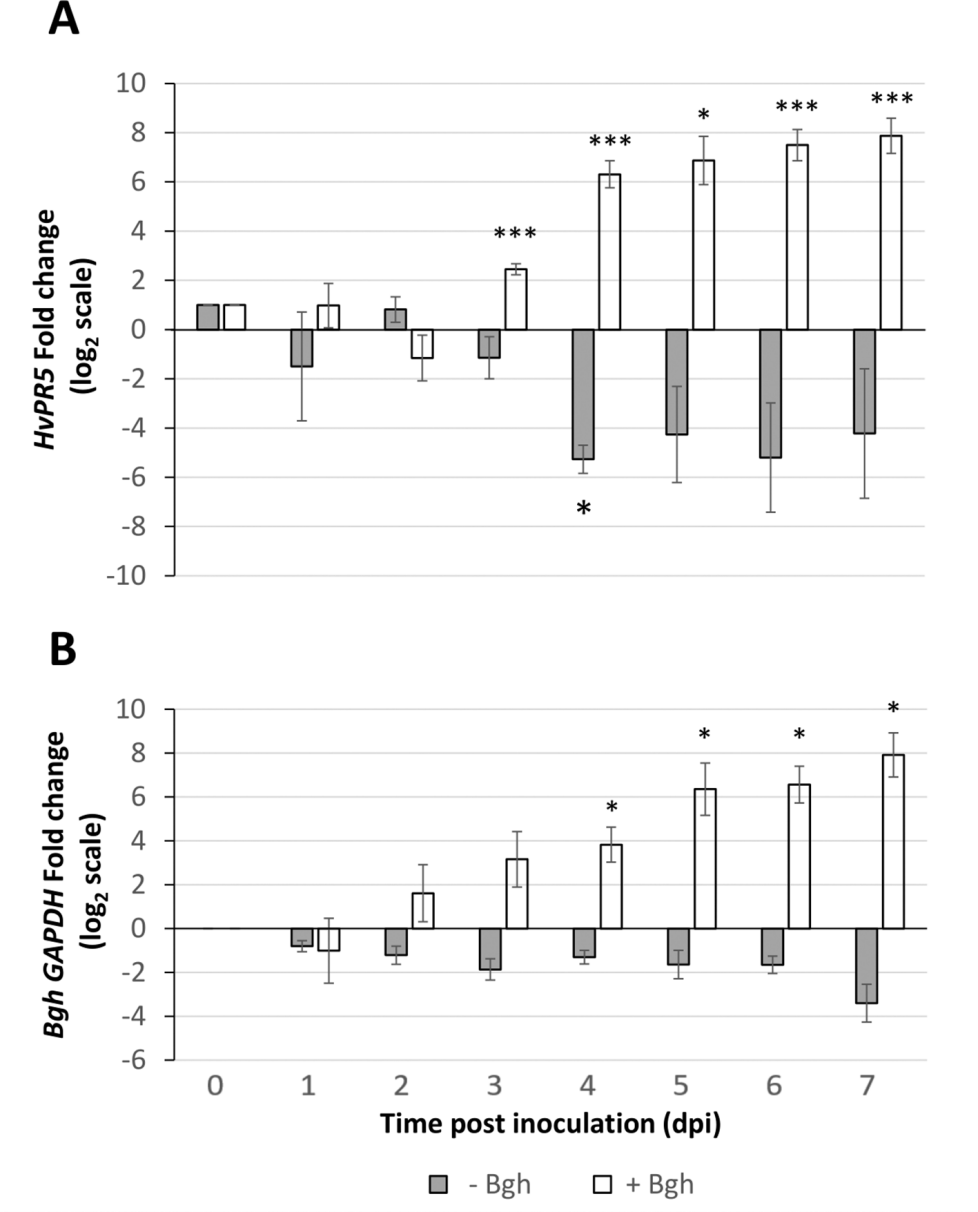
Time course showing relative expression level of **(A)**, the *PR5* isoform thaumatin-like protein 5 (*TLP5*) (KP293850.1/HORVU5HrG005180.6) and **(B)**, *BghGAPDH* (BLGH_00691) in control (grey) and *Bgh*-infected (white) barley leaves. In control (gray) and *Bgh*-infected (white) barley leaves. “Zero time point” corresponds to time immediately before inoculation of 7-day-old plants. Relative transcript levels were estimated by qRT-PCR, including *HvGAPDH* as reference gene. Relative expression levels were normalized to the 0-dpi time point and expressed as log_2_ fold change of the reference time point. Each data point shown is the mean ( ± SD) of three independent biological replicates, performed in three technical replicates. Significance was estimated by one-sample *t*-test. * indicates *P* < 0.05; *** indicates *P* < 0.001. qRT-PCR, quantitative reverse transcription polymerase chain reaction.

*TLP5* (KP293850.1) transcripts levels in whole primary barley leaves were similar to uninfected controls during the first stage of infection, increasing modestly at 3 dpi (6×, **Figure 4A**). This initial increase of the *TLP5* transcript happened after the increase of the powdery mildew housekeeping gene *BghGAPDH* at 2 dpi, meaning that the fungal biomass increased noticeably before the induction of *TLP5* (**Figure 4B**). Therefore, *TLP5* induction is likely to be a consequence of *Bgh* successful establishment rather than the onset of resistance in this compatible interaction setup. From 4 to 7 dpi, *TLP5* transcript levels rose from 100× to 380× increase, while levels in the uninfected control remained unchanged (**Figure 4A**). The drastic increase in transcript levels at later time points does correlate with the increased abundance of the TLP5 protein in 5-dpi infected epidermis.

A functional genomics approach was used to query whether the TLP5 protein isoform can influence the early stages of *Bgh* establishment in the barley host, a TIGS approach using short antisense phosphorothioate-modified oligodeoxyribonucleotides (PTOs) was adapted from previous work (Sun et al., 2005; Sun et al., 2007; Dinç et al., 2011). The method allowed for PTO uptake to whole excised barley leaves and thus resulting in silencing *TLP5* in all the cells of the treated leaves. To validate the assay, the recessive dominant susceptibility barley gene *MLO1*, whose loss of function has been reported to convey durable resistance to barley powdery mildew (Büschges et al, 1997), was included as a positive control.

**Figure 5:**
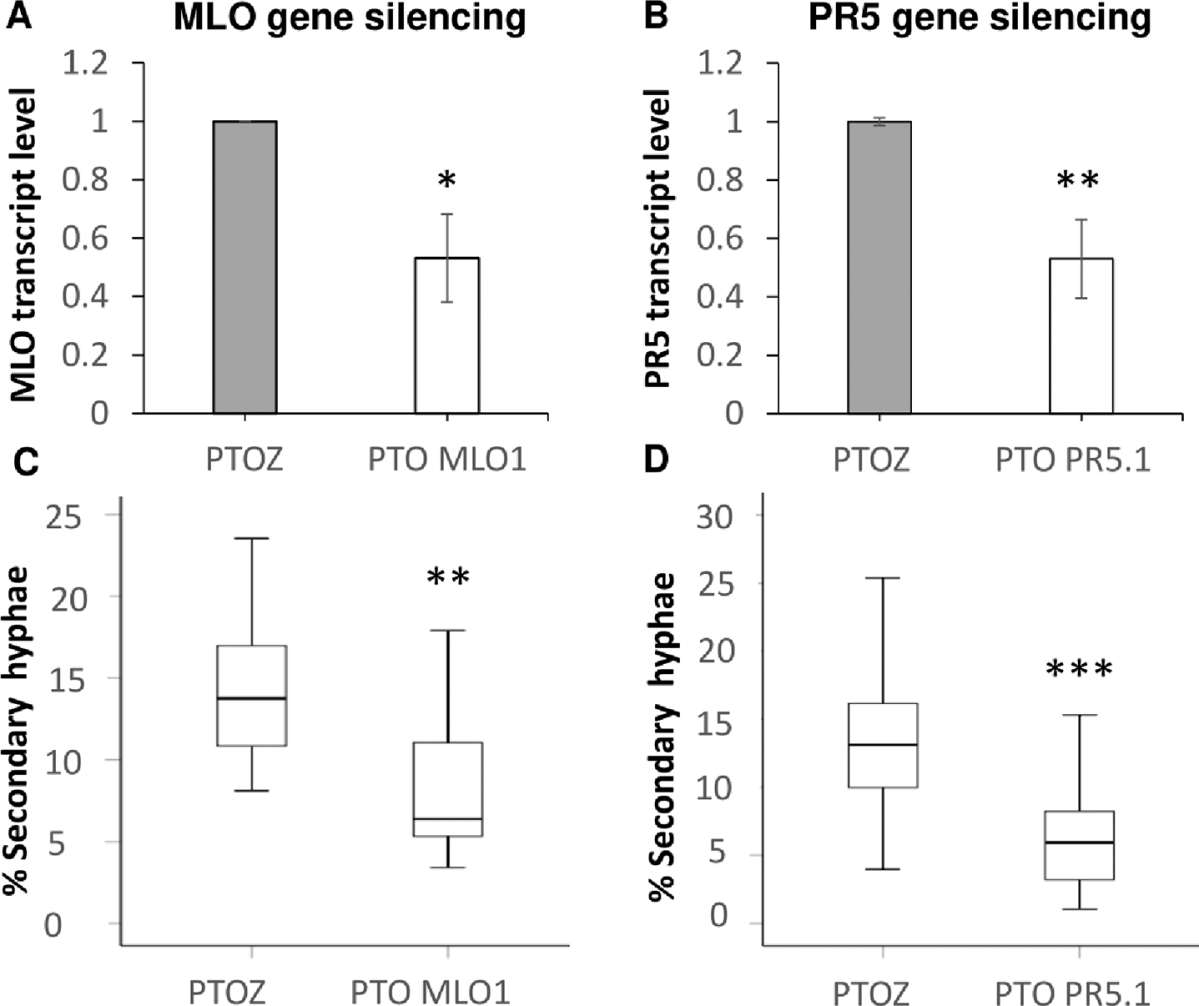
Transient-induced gene silencing (TIGS) of **(A)**, *MLO1* (HORVU4Hr1G082710), and **(B)**, *PR5* isoform *TLP5* (KP293850.1/HORVU5HrG005180.6); excised barley leaves were treated with PTO MLO.1 and PTO PR5.1 short antisense phosphorothioate-modified oligodeoxynucleotides for gene-specific silencing. PTOZ was used as negative control **(A)**. *TLP5* relative transcript levels estimated by qRT-PCR using PTOZ negative control for normalization. *HvGAPDH* was used as reference gene. Average of three (*MLO1*) and six (*TLP5*) independent biological replicates each analyzed in three technical replicates are shown. Significance was estimated by one-sample *t*-test. * indicates *P* < 0.05; ** indicates *P* < 0.01; *** indicates *P* < 0.001. Fungal development was compared between **(C)** *MLO1*- and **(D)** *TLP5*-silenced leaves with and PTOZ-treated control leaves, monitoring the proportion of conidia able to produce secondary hyphae (%SH). *N* = 19/43 total individual leaves analyzed for each treatment, spanning across four and seven independent biological replicates for *MLO1* and *TLP5* silencing experiments, respectively. Results are presented as boxplots showing median, interquartile, and minimum/maximum values. Significance was estimated with GLMM. * indicates *P* < 0.05; ** indicates *P* < 0.01; *** indicates *P* < 0.001. qRT-PCR, quantitative reverse transcription polymerase chain reaction; GLMM, generalized linear mixed model.

Care was taken to select gene-specific short antisense silencing oligos (ASOs) since several TLP isoforms with high similarity are present (**Table 3**). Preliminary treatments to silence *TLP5* were performed with two different PTOs (PR5.1 and PR5.2), and both gave very similar results for the infection phenotype (data not shown). This similar phenotype suggests the unlikely effect due to putative off-targets, since these were different for each of the PTOs. Moreover, these putative off-targets might not be silenced since the PTOs match only 17 or less nucleotides (data not shown; **Supplementary Material S4**). A sequence alignment revealed that PTO PR5.2 had a 100% sequence coverage and could potentially silence both HORVU5Hr1G051970.4 and KP293850.1/HORVU5HrG005180.6; therefore, the silencing experiment was only pursued with PTO PR5.1 to specifically target KP293850.1/HORVU5HrG005180.6. The silencing treatment with PR5.1 significantly reduced the levels of *TLP5* mRNA by 50% when compared with leaves treated with the PTO Z negative control. The reduction in *TLP5* transcript levels was similar to the reduction of *MLO1* transcripts in leaves treated with PTO MLO1 (**Figure 5A**). It can, therefore, be concluded that PTO MLO1 and PTO PR5.1 were efficient at silencing *MLO1* and *TLP5*, respectively, as monitored by qRT-PCR (**Figures 5A, B**). However, it cannot be ruled out that the silencing effect might be even greater than what was observed at the mRNA level, because PTO-based gene silencing can also act *via* translational arrest (Sun et al., 2005; Sun et al., 2007; Dinç et al., 2011). Nevertheless, data suggest that the TIGS assay is suitable for silencing barley genes expressed in barley leaves and relevant to the barley powdery mildew interaction. Moreover, it is a convenient method to evaluate gene expression at the whole-leaf level and can be exploited to investigate downstream phenotypic observations, such *Bgh* infection and reactive oxygen species (ROS) production, as described below.

### Silencing PR5 TLP5 Promotes Barley Resistance to Bgh Infection

The ability of conidia to produce secondary hyphae (%SH) was reduced by ca. 50% in *MLO1*- and *TLP5*-silenced leaves when compared with the PTOZ silencing control (**Figures 5C, D**). These data suggest that both MLO1 and TLP5 are required for the onset of infection, and thus, they are both involved in host susceptibility.

**Figure 6:**
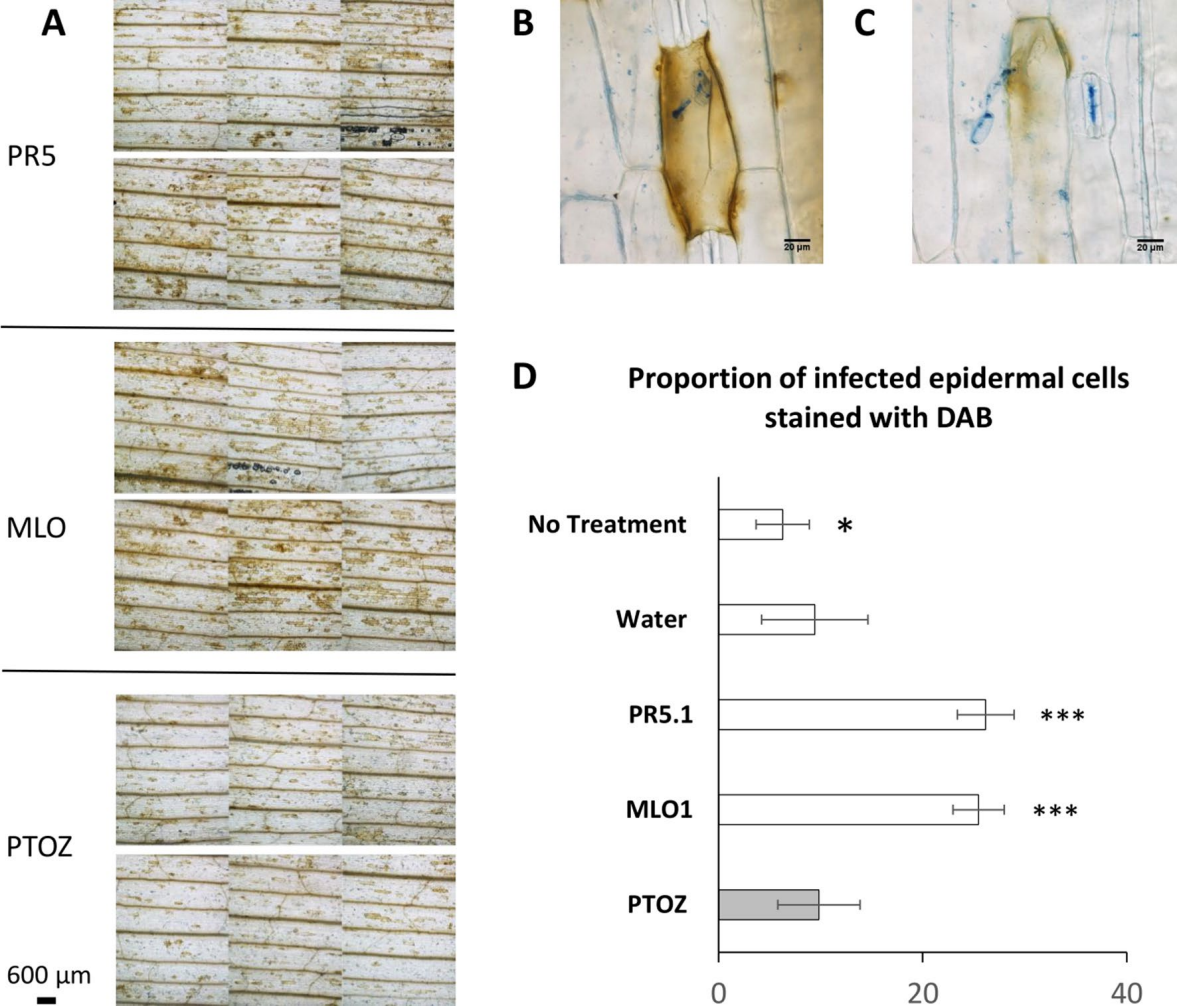
PTO MLO1-, PTO PR5.1-, and PTOZ-treated leaves, inoculated with *Bgh* conidia, were stained with 3,3′-diaminobenzidine (DAB) to detect H_2_O_2_ production in barley epidermal cells in response to *Bgh* inoculation at 18–22 hpi. Leaves were subsequently stained and cleared with lactophenol blue to visualize fungal structures and DAB-stained epidermal cells. **(A)**, Micrographs of PTO PR5.1-, PTO MLO1-, and PTOZ-treated leaves showing different intensities of DAB staining. Six panels are shown for each treatment, corresponding to each individual leaf from a typical experiment. Pictures were taken at 1.5 cm from the leaf tip, in bright field settings, at 40× magnification. **(B**–**C)**, Illustration of a DAB-stained barley cell indicating two degrees of H_2_O_2_ production in response to a germinated *Bgh* conidium. **(D)**, Graph showing the average (± SD) proportion of epidermal cells producing H_2_O_2_ following *Bgh* infection. This was monitored as the % of DAB-stained cells associated with germinated conidia (partial or whole cell response), comparing leaves from the PTOZ negative control treatment with PTO MLO1, PTO PR5.1, and the additional negative controls, “water,” and “no-treatment” leaves. *N* = 18 leaves from three independent biological replicates. Significance was calculated with one-way ANOVA and post-hoc Tukey HSD tests. Significance: * indicates *P* < 0.05; ** indicates *P* < 0.01; *** indicates *P* < 0.001. DAB, 3,3′-diaminobenzidine; ANOVA, analysis of variance; HSD, honestly significant difference.

Next, the generation of hydrogen peroxide, a marker of host resistance, was monitored at 18–22 hpi following *Bgh* infection in *MLO1*- and *TLP5*-silenced leaves. The presence of H_2_O_2_ was detected as a brown precipitate in leaves infiltrated with DAB (**Figures 6B, C**). Observations were first made from low-magnification micrographs (**Figure 6A** **Supplementary Figure 1** in, **Supplementary Material S5**). Both *MLO1*- and *TLP5*-silenced leaves showed a marked increase of brown areas in addition to the vascular tissues, than did the PTOZ silencing control, water, or absence of treatment. This could also be quantified as an increase in the number of brown-stained cells per defined surface area (**Supplementary Material S4**). In a more detailed observation, both *MLO1*- and *TLP5*-silenced leaves displayed a significant increase in the proportion of infected cells that appeared brown overall or for a large part of the cell surface area, indicating an increase in ROS production (**Figure 6D**). This suggests that *TLP5*-silenced leaves were more capable of producing hydrogen peroxide in response to the *Bgh* infection, as this was also the case and expected for *MLO1*-silenced leaves.

## Discussion

Barley powdery mildew is a well-studied pathosystem for which haustorial structures play a central role for biotrophy. But despite the investigation of the proteome of *Bgh* haustoria (Godfrey et al., 2009), this is the first attempt at probing the protein makeup of the host extrahaustorial structures surrounding *Blumeria* haustoria. Moreover, to the best of our knowledge, it is also the first report of a large-scale proteome analysis of extrahaustorial structures for any haustorium-bearing pathosystems. In addition, there are no proteomics data of barley epidermis following *Bgh* infection. Only transcriptomics data are available to describe differential gene expression in *Bgh*-infected barley epidermis in compatible and *MLO*-resistant interactions (Gjetting et al., 2004; Zierold et al., 2005). This proteomics study is, therefore, justified as a resource for data mining and exploring new lines of investigation. This is initially based on correlative evidence of DAPs in epidermis caused by *Bgh* infection and protein relocation to haustorial associated structures, such as the host-originated extrahaustorial membrane and matrix (EHM and EHMx). With the use of the workflow presented in this study, candidate gene products that are putatively toxic to the pathogen or that are modulating plant immunity or susceptibility, such as the PR5 isoform TLP5 or MLO1, can then be validated by functional genomics with an *in planta* TIGS assay adapted from existing protocols (Sun et al., 2005; Sun et al., 2007; Dinç et al., 2011), exploiting the delivery of short antisense gene-specific phosphorothioate modified oligodeoxriboynucleotides (PTOs). Due to the obligate biotrophic nature of powdery mildews, and the effort it takes to generate stable transformed barley plants, a straightforward and easy-to-implement transient *in planta* gene silencing assay is required. We successfully used the PTO-based TIGS assay to evaluate the gene silencing efficiency, the phenotype of infection, and host responses such as ROS production at the whole-leaf level rather than being confined to a small proportion of singly transformed cells within the leaf, as it is the case with the previously described TIGS assay that biolistically delivered plasmids carrying a hairpin sense/antisense construct (Nowara et al., 2010). Moreover, the PTO-based TIGS assay does not require gene cloning or plasmid construction and should be specific enough to potentially silence a single gene when targeting one particular isoform within a gene family. Neither does the assay require viruses potentially pathogenic to the host, thus modulating the subsequent host immune responses to fungal infection (Tufan et al., 2011).

### Putative Resistance or Susceptibility Roles of Proteins With Increased Abundance in Infected Epidermis or Haustoria-Associated Structures

DAPs were identified as more abundant in the epidermis of barley leaves following infection with powdery mildew or increased in haustoria-enriched fractions as the consequence of specific (re)location of proteins to EHM or EHMx structures associated with haustoria, and of host origin, although in the infected vs uninfected epidermis samples’ comparison (IE vs UE), some DAP proteins associated with plastids were identified as more abundant in the infected tissue. This might be attributed to increased mesophyllic contamination occurring during the harvesting of the infected epidermis, since epidermis samples were harder to strip from infected leaves than from healthy leaves. However, the GO categories related to chloroplasts were not overrepresented in the list of more abundant DAP proteins in infected epidermis (IE vs UE). Also, chloroplastic-related proteins were even less prominent within the list of DAPs that were more abundant in haustoria when compared with infected epidermis (Hau vs IE). This suggests that haustoria were effectively enriched and that such technical bias has less impact when comparing haustoria with epidermis. Moreover, many of the DAPs observed in IE were not predicted to be chloroplastic and were previously shown to be relevant in modulating plant responses to pathogens. Many “classical” secreted PR protein isoforms were identified as more abundant in infected epidermis in the IE vs UE comparison. These included cysteine-rich venom proteins, an oxalate oxidase-like protein or germin, chitinases, TLPs, and endo-1,3-β-glucanases, classified as PR1, PR2, PR3, PR5, PR9, and PR16 (van Loon et al., 2006). Other DAPs more abundant in IE (in the IE vs UE comparison) included stress response proteins, such as HSPs, and enzymes involved in redox homeostasis. Some additional examples are discussed below.

The present study also allowed to identify host proteins specifically associated with haustoria, as their abundance was higher than in infected epidermis. Strikingly, many ATP synthase subunits and an ADP/ATP transferase were identified as more abundant or exclusive to the haustoria-enriched fractions. An ER-associated BiP/HSP70C protein, proteases, as well as membrane-associated or signalling proteins such as an aquaporin, an early nodulin-like protein, and a cysteine-rich receptor-like protein kinase 25 (CRK25) were also more abundant in the haustoria-enriched fractions.

### Putative Role of PR5 Isoforms in the Barley Powdery Mildew Arms Race

PR5 proteins, or TLPs, are a large family of proteins, well and long known for their antifungal activity (Vigers et al., 1992). Different antifungal modes of action have been attributed to TLPs, such as pore making in membranes (Roberts and Selitrennikoff, 1990) and β-1,3-1,4- or β-1,3-glucan (callose)-binding capacity (Osmond et al., 2001; Singh et al., 2017). Many studies have shown that PR5, in particular *TLP5*, is induced by biotic stresses and has been associated with resistance, since it is more rapidly induced in resistant than in susceptible barley cultivars challenged by powdery mildew (Scheler et al., 2016). In the present study, the barley cultivar used was fully susceptible to *Bgh* infection, and *TLP5* expression was only increased 3 days after infection. In addition to the *Blumeria* effector BEC1054 (CSEP0064), which interacts directly with the barley PR5 isoform TLP5 (Pennington et al., 2016), there are several instances where it was shown that plant PR5 isoforms directly interacted with effector proteins produced by pathogenic fungi, placing PR5 at the center stage of the arms race. This includes the PevD1 effector from *Verticillium dahliae* (Zhang et al., 2018) and the *Botrytis cinerea* elicitor BClEB1 (González et al., 2017). It was suggested that such protein interactions can potentially protect the invading fungus from PR5 toxicity (González et al., 2017). Furthermore, it was shown that the PevD1 effector from *V. dahliae* also interacts with NPR1, a salicylic acid-responsive transcription factor that regulates PR protein expression (Zhang et al., 2018). As PR5 deletion increased susceptibility in the above instances, PR5 is either inhibiting the action of the effector or the effector is attempting to suppress PR5-mediated resistance.

Conversely, in the present study, TIGS experiments to knock down the *TLP5* isoform led to the opposite effect (i.e., *TLP5* silencing reduced fungal infection), suggesting that PR5 is required for susceptibility in a barley–*B. graminis*-compatible interaction. However, it is important to note that the negative control for the TIGS treatment had higher levels of TLP5 than otherwise unhandled leaves at 2 dpi following *Bgh* infection (**Supplementary Material S4**, section 3). This might explain to some extent that there is a noticeable infection phenotype and plant response phenotype in *TLP5*-silenced leaves. However, it should be taken into account that TLP5 is unlikely to act alone but acts together with additional TLP isoforms. Thus, redundancy cannot be excluded, and silencing of one isoform might be compensated by some of over 30 isoforms predicted in the barley genome. Moreover, the TIGS experiment was optimized for high *Bgh* infection, using a highly susceptible barley cultivar inoculated under a low-light, high-humidity regimen. It is widely accepted that plant immunity is modulated by light, depending on active chloroplasts for the production of ROS and PR protein accumulation, which may trigger programmed cell death (PCD)/hypersensitive response (HR) (Delprato et al., 2015). Therefore, the impact of *TLP5* and other *PR5* silencing on expression and *Bgh* infection should be investigated under different light regimens.

### The Other “Classical PR Proteins to Revisit”: As Antifungal Proteins or as Ergosterol Detectors?

It has been reported extensively that PR1, PR2, and PR5 have antifungal properties (van Loon et al., 2006). Their modes of action are still mainly enigmatic. PR1 proteins might have activity towards fungi and oomycetes through their ergosterol-binding properties, either for toxicity or for the perception of pathogen-associated molecular patterns (PAMPs) (Breen et al., 2017). It was previously reported that ergosterol can act as a PAMP, triggering cell responses similar to PTI (Rossard et al., 2010). In the barley–*Bgh* interaction, it could be expected that PAMPs such as ergosterol are likely to be perceived at the haustorial interface, since haustoria are the only structures within the leaf, the conidiospores and hyphae being epiphytic. Nevertheless, none of the classical PR proteins accumulated preferentially in haustoria-enriched samples, despite being more abundant in epidermis in a late stage of infection. Therefore, it would be interesting to further investigate the ergosterol-binding capability of suitable candidates such as the non-specific lipid-binding protein that was more abundant in the haustoria-enriched samples. Ergosterol was also shown to be a potent inhibitor of plant plasma membrane ATPases, thus blocking proton efflux, leading to alkalinization of the extracellular space (Rossard et al., 2010). The alkalinization can trigger downstream responses, such as ROS production. Although ergosterol is an accepted PAMP, its perception and signalling mechanisms are still mainly unclear (Klemptner et al., 2014). It can, therefore, be speculated that the increased presence of V-type ATPases at the EHM might allow for the perception of ergosterol through its ATPase inhibitory effect, leading to extracellular alkalinization or reduced acidification of the extrahaustorial space.

### Additional Proteins With Putative Antifungal Activity

A protein described in the barley database as a cysteine-rich receptor-like protein kinase 25 (CRK25) was more abundant in the haustorial fraction than in infected epidermis. Although it has been labelled as a CRK, a sequence homology search established that CRK25 is homologous to cysteine-rich repeat secretory proteins 55. These proteins lack the ser/threonine kinase and transmembrane domains typical of CRKs and only possessed the extracellular cysteine-rich domain pfam01657, previously named “domain of unknown function 26” DUF26 (Yadeta et al., 2017). The DUF26/pfam01657 domain is often duplicated and has been implicated in salt stress response and antifungal activity. For instance, the DUF26-containing protein ginkbilobin 2 (GKN2) from *Ginkgo biloba* has antifungal properties, while it acts as a lectin-binding mannose (Miyakawa et al., 2014). Based on these interesting properties, it will be interesting to investigate further the role of this protein in the barley powdery mildew pathosystem.

### GPI-Anchored Proteins and Other Plasmalemma-Associated Proteins

The plasma membrane microdomains or lipid rafts are usually associated with GPI-anchored proteins involved in signalling pathways. GPI proteins related to modulating immunity included plasmodesmatal PDLP1 protein, the *PMR4* callose synthase, or RPW8 protein (as reviewed in Faulkner, 2015), as well as a remorin associated with *Phytophthora* haustoria (Bozkurt et al., 2014). In the present study, the early nodulin-like protein 9, associated with haustoria, was predicted with a GPI anchor. It is now emerging that early nodulin-like proteins are not only involved in legume–rhizobacteria nodulation but may also contribute to pathogen fitness by acting as nutrient and signal transporters as reviewed (Denancé et al., 2014).

In the present study, an aquaporin with sequence homology to PIP2 isoforms was also shown to be more abundant in the haustoria-enriched fraction. Aquaporins are intrinsic plasma membrane proteins, initially believed to be primarily involved in water transport, but some isoforms were shown to transport other molecules such as hydrogen peroxide (Tian et al., 2016). Aquaporins are likely key players at the plant–pathogen interface, as they have been shown to be targets of pathogen effectors, as exemplified by the rice PIP 1;3 aquaporin targeted by the Hpa1 protein that is a component of the type III secretion system and facilitate *Xanthomonas oryzae* effectors delivery (Li et al., 2019).

### ATPases

An ATPase transcript was shown to be induced in infected barley leaves, and its cognate ATPase protein interacted with a 14-3-3 protein (Finni et al., 2002). In more recent studies, it was shown that the well-known regulator of plant immunity RIN4 interacts with the plasma membrane ATPAses AHA1/2, a remorin, and the resistance gene RPS2 in *Arabidopsis* (Liu et al., 2009). It was proposed that 14-3-3 and RIN4 binding to AHA1/2 may modulate the ATPAse activity. Previous proteomics studies have identified P-ATPases, aquaporins, leucine-rich repeat receptor kinases, a remorin, and NAPH oxidases in detergent-resistant membranes (Takahashi et al., 2013). Therefore, it will be interesting to follow up the role of V-ATPases co-localized to haustorial structures in order to identify their interactors and query their association to lipid rafts. In addition to this, a link might be made with the perturbation of V-ATPase activities by ergosterol (as discussed above), thus influencing also the makeup of the plasma membrane microdomains (Rossard et al., 2010; Klemptner et al., 2014).

In our study, the modulation of the acidification of the EHMx might also influence the activity of acidic proteases such as aspartate protease shown to accumulate in haustoria. Acidification of the extracellular space might also influence cell wall loosening.

### Aspartic Proteases

Proteases have been shown to play an active role at different levels of resistance including in the perception of pathogens *via* microbe-associated molecular pattern (MAMP)-triggered immunity, ETI, PCD, and systemic acquired resistance (Balakireva and Zamyatnin, 2018). In the present study, one aspartate protease was exclusive to haustoria-enriched samples. In a transcriptomic study, an aspartic protease and a subtilase-like protease were induced following *Bgh* infection and were more abundant in epidermis than in mesophyll (Zierold et al., 2005).

*Arabidopsis* mutants overexpressing the endogenous secreted aspartate protease CDR1 were more resistant to *Pseudomonas syringae* infection in a salicylic acid-dependent manner. PR1 and PR2 were increased in these overexpressing mutants. PR1 and PR2 expression was also induced by external application of CDR1. Interestingly, CDR1 shares some amino acid sequence similarity with a cathepsin-D (Xia et al., 2004). *Arabidopsis* plants overexpressing the grapevine Asp protease AP13 were also more resistant to a powdery mildew pathogen but were more susceptible to *Botrytis* infection (Guo et al., 2013; Guo et al., 2016). Therefore, the role of the aspartate proteases identified in this study should be investigated further.

### Cell Wall-Associated and Cell Wall-Modifying (Polysaccharides) Enzymes

Xylans are the main hemicellulositic components of cereal cell walls. Following barley powdery mildew infection, arabinoxylans were shown to be a major constituent of barley papillae, alongside callose and cellulose, to provide penetration resistance (Chowdhury et al., 2014). This was evidenced by two glycosyltransferases associated with xylan biosynthesis, GT43 and GT47, which were shown to contribute to penetration resistance during *Bgh* infection (Chowdhury et al., 2017). Moreover, changes in the plant cell wall, as well as cell wall degradation products, can trigger the perception of a microbe invader *via* degradation-associated molecular patterns (DAMPs) (Bacete et al., 2018). Various cell wall-degrading enzymes associated with haustoria or infected epidermis (several β-1,3(1,4)-glucosidases, an arabinofuranosidase inhibitor, and a xylanase inhibitor) were identified in this study. The arabinofuranosidase, detected as more abundant in haustoria, might act as a cell wall-degrading enzyme producing oligosaccharides, which might be generating molecules that might be relevant for the perception of *Bgh* by the host, *via* either PAMPs or DAMPs.

### HSPs, BiPs, and Cyclophilins to Cope With ER Stress or Their Involvement in Plant Immunity

Several HSPs, a HSP40/DNAJ protein with a cysteine-rich domain, and an HSP70, as well as cyclophilins, were more abundant in the infected epidermis when compared with the uninfected controls. There was also an HSP70C protein exclusive to the haustoria-enriched fraction. HSP70C has a high sequence similarity to the ER BiP protein, involved in ER stress and plant immunity, *via* folding quality control monitoring of secreted proteins such as plasma membrane-located PAMPs or pattern recognition receptors (PRRs) (as reviewed in Park and Seo, 2015). ER resident chaperones, such as BiP and HSP40/DNAJ, are involved in protein folding within the ER lumen. These chaperones assist the ER-associated degradation (ERAD) pathway, which drives unfolded proteins to proteasome degradation and eventually may trigger PCD (Howell, 2013). In *Arabidopsis*, HSP70 was shown to be required for resistance and was targeted by the *P. syringae* effector HopI1, with which it forms a complex. HopI1 highjacked plant resistance and increased HSP70 ATPase activity *in vitro* (Jelenska et al., 2010). Kwaaitaal et al. (2017) showed that EHMs from *Bgh*-infected barley shared properties with ER membranes (2017); thus, it is not surprising to identify ER proteins in haustoria-enriched samples.

Cyclophilins and relative peptidyl-prolyl *cis*–*trans* isomerases (PPIases) were identified as more abundant proteins in infected epidermis in the present study. These proteins catalyze protein folding and can act as chaperones. They have been involved in both pathogen virulence and plant susceptibility. For instance, the *P. syringae* effector avrRPS2 protease was shown to be activated by the *Arabidopsis* cyclophilin CPR1 (Coaker et al., 2005). In another case, a cyclophilin was shown to activate the Nudix hydroxylase activity of the *Phytophthora* effector Avr3b (Kong et al., 2015). It seems that cyclophilins and HSPs might act in concert, as HSP90 has been shown to directly interact with large-molecular-weight FKBP cyclophilins in mammals, to translocate proteins to the nucleus (Pratt et al., 2001).

### Pyruvate Metabolism and TCA Cycle

The abundance of several enzymes involved in pyruvate metabolism and the TCA cycle were increased in either haustoria (aconitate hydratase) or infected epidermis (malate dehydrogenase and an enzyme belonging to the pyruvate dehydrogenase complex). This might corroborate with a metabolomics study, which showed that malate was one of the metabolites with most increased abundance in barley following a compatible infection with Magnaporthe grisea. On the other hand, the NADP-malic enzyme accumulates at sites of infection, mainly in incompatible interactions, and is likely involved in NADPH synthesis for ROS production by mitochondrial perturbation of the TCA pathway (Parker et al., 2009). In another study, an Arabidopsis mutant accumulating an excess of NADH in chloroplasts triggered an excess of malate production (Zhao et al., 2018). In turn, this excess of malate was translocated from chloroplasts to mitochondria, where malate was metabolized, generating NADH and itself triggering mitochondrial ROS, which finally led to PCD (Zhao et al., 2018). In addition, ectopic application of malate to HELA cells led to increased PCD (Zhao et al., 2018). During Bgh infection, a barley malate dehydrogenase is the target of the Bgh effector BEC1054 (CSEP0064) (Pennington et al., 2016). Thus, all these findings suggest the importance of the TCA cycle as a potential source of nutrients for the pathogen, as well as a pathway for PCD in the host cell.

## Conclusion

Coupling a proteomics study of infected epidermis and haustorial complexes with a TIGS method has the potential to discriminate between and identify novel resistance and susceptibility factors in barley powdery mildew. This study is also overcoming challenges associated with functional genomics studies involving an obligate pathogenic fungus such as *B. graminis*, adapting a transient gene silencing (TIGS) approach. It allowed to show that the barley PR5 isoform TLP5, with increased abundance in infected epidermis, acted like a susceptibility gene, since *TLP5* silencing led to decreased infection and increased ROS production in a similar fashion to *MLO*-silenced leaves. Furthermore, this initial proteomics study has identified proteins preferentially associated with extrahaustorial structures. Several proteins are interesting potential modulators of plant immunity and thus are worth of further investigation. An improved large-scale proteome analysis of purer haustorial complexes will aid in revealing additional modulators of the barley–*Bgh* interaction, opening novel routes of investigations; and following validation of the key players by functional genomics, it will be possible to devise new strategies to control powdery mildew diseases, by targeting either host susceptibility genes or the fungus pathogenicity genes, exploiting the ectopic delivery of gene silencing oligodeoxynucleotides.

## Data Availability Statement

The datasets generated for this study can be found in PRIDE Archive, PXD012684.

## Author Contributions

LB initiated, designed, and led the project; advised on experimental work and analysis; and was the lead writer of the manuscript. SL co-designed, performed, analyzed proteomics work and silencing experiments, performed statistical analysis, prepared figures, and wrote the manuscript. KO designed silencing workflow and led PR5/MLO silencing experiments, performed statistics, and prepared figures, SDG performed qRT-PCR experiment and analysis, prepared figures, and edited the manuscript. SG and JF conducted gene silencing experiments and their validation. RW and RC contributed and advised on proteomics data processing.

## Funding

This work was carried out thanks to funding from BBSRC (UK) DTP studentships to Imperial College and Royal Holloway University of London (SL and KO grants # BB/M011178/1 and BB/J014575/1 respectively), BBSRC and Syngenta iCASE studentship (SDG, BB/N503897/1), and British Society of Plant Pathology (BSPP) MSc studentship (SL).

## Conflict of Interest

The authors declare that the research was conducted in the absence of any commercial or financial relationships that could be construed as a potential conflict of interest.

## Footnotes

1
http://www.matrixscience.com

2
http://plants.ensembl.org

3
https://biit.cs.ut.ee/gprofiler/page.cgi?welcome

4
https://wolfpsort.hgc.jp/links.html

5
http://www.cbs.dtu.dk/services/TMHMM /

6
http://www.cbs.dtu.dk/services/DeepLoc/

7
http://gpcr.biocomp.unibo.it/predgpi/

8
http://www.cbs.dtu.dk/services/SignalP/

9
http://www.ncbi.nlm.nih.gov/

10
http://rna.urmc.rochester.edu/cgi-bin/server_exe/oligowalk/oligowalk_form.cgi

11
http://www.R-project.org/

## Abbreviations

*Bgh*, *Blumeria graminis* f. sp. *hordei*; DAPs, differentially abundant proteins; EHM, extrahaustorial membrane; EHMc, extrahaustorial complex; EHMx, extrahaustorial matrix; HAU, haustoria-enriched fraction; IE, infected epidermis.

## Supplementary Material

Supplementary Materials S1 Proteomics data analysis: Mascot results indicating protein IDs and normalised Spectral counting data for protein quantification.

Supplementary Materials S2 Gene ontology and protein cellular compartment predictions.

Supplementary Materials S3 Protein sequence and homology information of *TLP5* and other PR5 proteins.

Supplementary Materials S4
*TLP5* expression, PTO sequences for gene silencing and impact of transient induced gene silencing on infection process.

Supplementary Materials S5
**Supplementary Figure 1** containing Micrographs of DAB stained leaves for H2O2 monitoring in response to Bgh infection in MLO1 and TLP5 silenced leaves.
