# Supplementary material for: Analysis of Barley Leaf Epidermis and Extrahaustorial Proteomes During Powdery Mildew Infection Reveals That the PR5 Thaumatin-Like Protein TLP5 Is Required for Susceptibility Towards Blumeria graminis f. sp. hordei
Source: Front Plant Sci. 2019 Oct 30;10:1138. doi: 10.3389/fpls.2019.01138 (PMC6831746; doi:10.3389/fpls.2019.01138)
Supplement: Supplementary Materials S3 — Protein sequence and homology information of TLP5 and other PR5 proteins. [file Table_3.docx]

**Supplementary materials 3**

Supplementary materials detailing nucleotide and amino acid sequence alignments of Thaumatin-like transcripts and proteins. It also includes PTO sequence information for gene silencing and sequence homology search (BLAST) results for *TLP5* and PTOs PR5.1/PR5.2.

**Contents**

[Section 1 Thaumatin-like isoforms accession number and information 1](#_Toc1677775)

[Section 2 Sequences of phopsphothiorate modified short silencing oligodeoxynucleotides used to target *MLO1* and *TLP5* 2](#_Toc1677777)

[Section 3 Comparison of amino acid sequences of four thaumatin-like proteins identified as proteins in this study or elsewhere. 3](#_Toc1677779)

[Section 4 Comparison of CDS of four thaumatin-like proteins 4](#_Toc1677781)

[Section 5](#_Toc1677782) [BLAST search of *TLP5* CDS against *Hordeum vulgare* database 7](#_Toc1677783)

[Section 6 BLAST search of PTO PR5.1 against *Hordeum vulgare* database 24](#_Toc1677784)

[Section 7 BLAST search of PTO PR5.2 against *Hordeum vulgare* database 26](#_Toc1677785)

##

## Section 1

## Thaumatin-like isoforms accession number information

| **Gene ID** | **Uniprot KB ID** | **Protein Description** |
| --- | --- | --- |
| HORVU5Hr1G005180.6/ KP293850.1 | O23997 | Thaumatin-like protein 5 (*TLP5*) |
| HORVU5Hr1G051970.4 | A0A287R7D5 | Pathogenesis-related thaumatin superfamily protein |
| HORVU4Hr1G002650.2 | M0W099 | Pathogenesis-related thaumatin superfamily protein |
| HORVU5Hr1G005180.1 | A0A287Q7Z4 | Pathogenesis-related thaumatin superfamily protein |

## Section 2

## Sequences of phosphorothioate modified short silencing oligodeoxynucleotides used to target *MLO1* and *TLP5*

| **Gene targeted** | **PTO name** | **PTO sequence** | **Sequence on gene target (Reverse Complement of PTO seq)** | **Gene target ID** |
| --- | --- | --- | --- | --- |
| *HvMLO1* | PTO MLO1 | TAGTCAACGTACTTGCTGG | CCAGCAAGTACGTTGACTA | HORVU4Hr1G082710 |
| *HvTLP5* | PTO PR5.1 | TTGAAGAACATTGAGTAGT | ACTACTCAATGTTCTTCAA | HORVU5Hr1G005180.6 |
|  | PTO PR5.2 | AAGAAGTCCTTGTTCGCGC | GCGCGAACAAGGACTTCTT | HORVU5Hr1G005180  HORVU5Hr1G051970 |
| Negative Control  *Z* seed protein  (No target in leaves) | PTOZ | AAGCGGTTGAGCACTGAA | TTCAGTGCTCAACCGCTT | GenBank: X97636.1 |

## Section 3

## Comparison of amino acid sequences of four thaumatin-like proteins experimentally identified as proteins in this study or elsewhere.

**A:** Multiple alignment of protein sequences


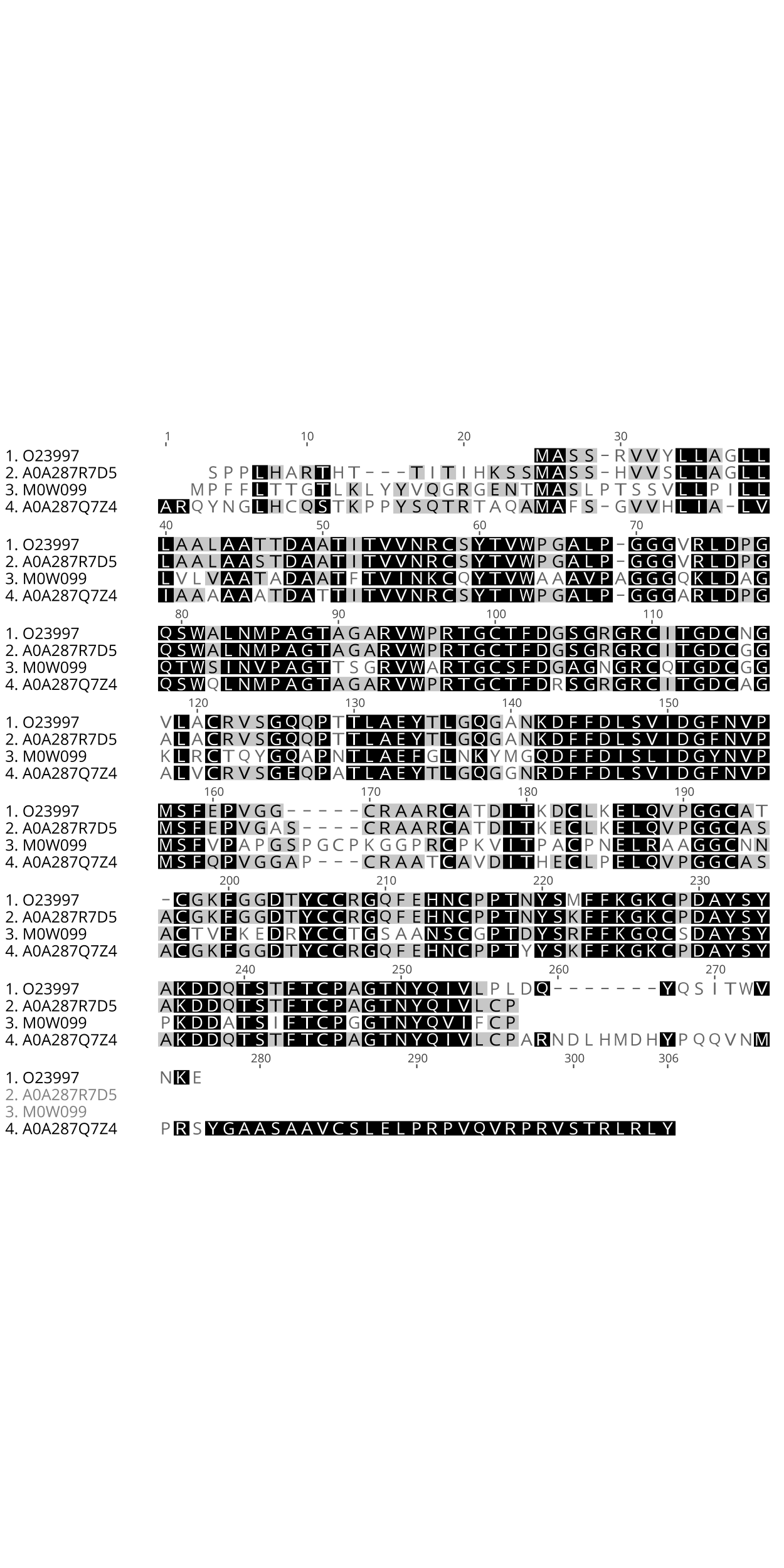


Geneious Prime^®^ (version 11.1.3) alignment using global alignment with free end gaps, using the cost matrix based upon identity (1.0/0.0). Gap open penalty =12, Gap extension penalty =3, refinement iterations =2.

**B: %** Identity of protein amino acid sequences.

| % Identity | O23997 | A0A287R7D5 | M0W099 | A0A287Q7Z4 |
| --- | --- | --- | --- | --- |
| O23997 |  | 93% | 54% | 78% |
| A0A287R7D5 | 93% |  | 52% | 79% |
| M0W099 | 54% | 52% |  | 48% |
| A0A287Q7Z4 | 78% | 79% | 48% |  |

## Section 4

## Comparison of CDS of four thaumatin-like proteins

**A:** Multiple alignment of four thaumatin-like CDS with free gaps based on identity. PTOs PR5.1 and PR5.2 have been annotated onto the CDS of HORVU5Hr1G005180.6.


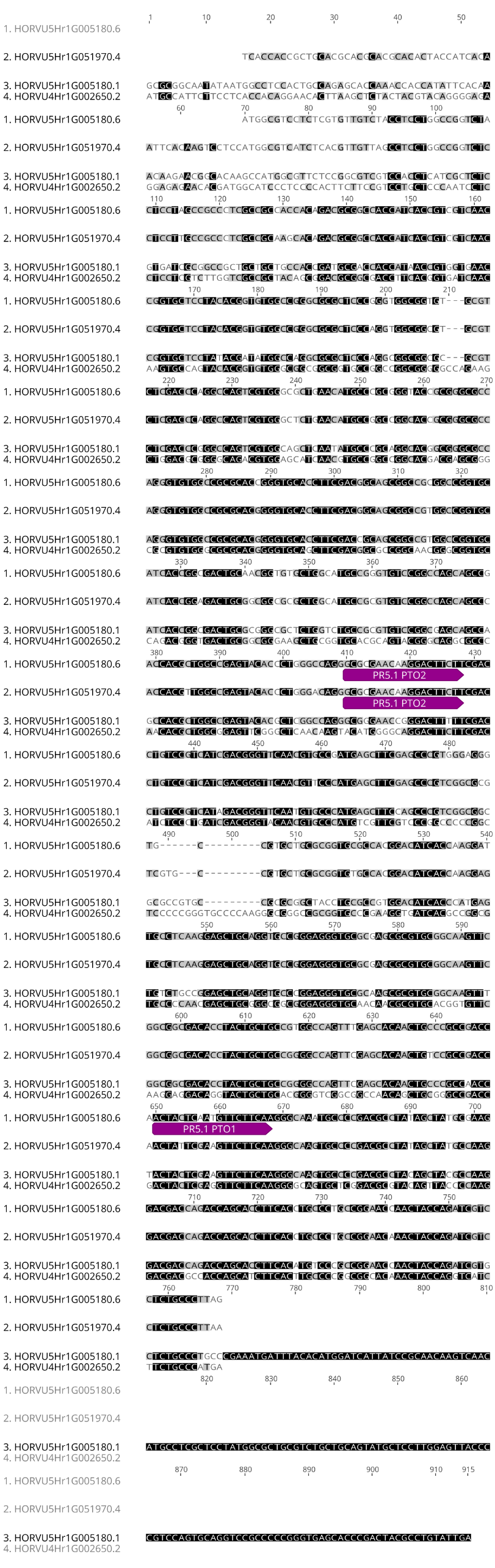


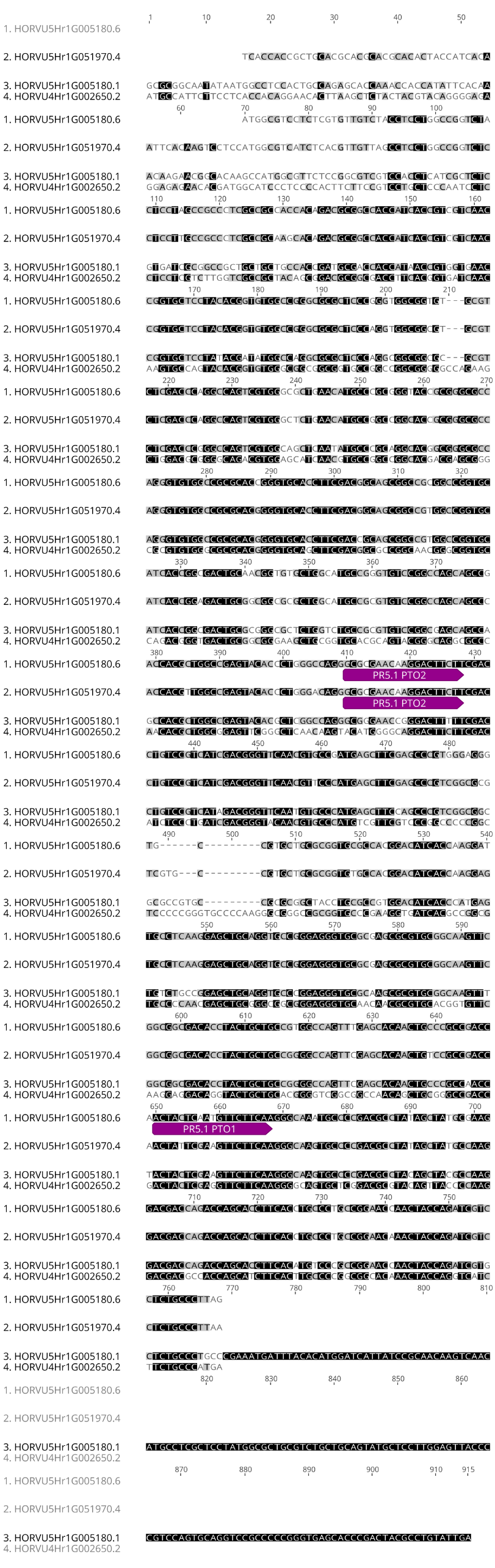


Alignment was performed with Geneious Prime^®^ (version 11.1.3) alignment using global alignment with free end gaps, using the cost matrix based upon identity (1.0/0.0). Gap open penalty =7, Gap extension penalty =3, refinement iterations =2.

**B: %** Identity of nucleotide sequences

|  | HORVU5Hr1G005180.6 | HORVU5Hr1G051970.4 | HORVU5Hr1G005180.1 | HORVU4Hr1G002650.2 |
| --- | --- | --- | --- | --- |
| HORVU5Hr1G005180.6 |  | 93% | 82% | 65% |
| HORVU5Hr1G051970.4 | 93% |  | 79% | 63% |
| HORVU5Hr1G005180.1 | 82% | 79% |  | 59% |
| HORVU4Hr1G002650.2 | 65% | 63% | 59% |  |

##

## Section 5

## BLAST search of *TLP5* CDS against *Hordeum vulgare* database

BLASTS (v.2.7.1+) were performed using the EMBL web tool against the Hordeum_vulgare.IBSC_v2.cdna.all database (48,391 sequences; 478,586,041 total letters)

BLAST outputs with the first 50 results are shown below, followed by the nucleotide alignment of significant alignments.

(<http://plants.ensembl.org/Multi/Tools/Blast?db=core>)

Reference: Stephen F. Altschul, Thomas L. Madden, Alejandro A.

Schaffer, Jinghui Zhang, Zheng Zhang, Webb Miller, and David J.

Lipman (1997), "Gapped BLAST and PSI-BLAST: a new generation of

protein database search programs", Nucleic Acids Res. 25:3389-3402.

Query= ENA|KP293850|KP293850.1 Hordeum vulgare Thaumatin like protein 5

TLP5 (*TLP5*) mRNA, complete cds.

Length=719

Score E

Sequences producing significant alignments: (Bits) Value

EG:**HORVU5Hr1G005180.6** cdna chromosome:IBSC_v2:chr5H:8614116:8615... 1425 0.0

EG:HORVU5Hr1G051970.4 cdna chromosome:IBSC_v2:chr5H:406435574:40... 579 1e-163

EG:HORVU5Hr1G051970.7 cdna chromosome:IBSC_v2:chr5H:406435630:40... 579 1e-163

EG:HORVU5Hr1G051970.6 cdna chromosome:IBSC_v2:chr5H:406435630:40... 579 1e-163

EG:HORVU5Hr1G051970.5 cdna chromosome:IBSC_v2:chr5H:406435615:40... 571 3e-161

EG:HORVU5Hr1G051970.1 cdna chromosome:IBSC_v2:chr5H:406435550:40... 442 2e-122

EG:HORVU5Hr1G051970.3 cdna chromosome:IBSC_v2:chr5H:406435574:40... 442 2e-122

EG:HORVU5Hr1G051970.2 cdna chromosome:IBSC_v2:chr5H:406435574:40... 442 2e-122

EG:HORVU5Hr1G005180.5 cdna chromosome:IBSC_v2:chr5H:8598547:8736... 295 3e-78

EG:HORVU5Hr1G005180.1 cdna chromosome:IBSC_v2:chr5H:8595475:8736... 295 3e-78

EG:HORVU5Hr1G005180.3 cdna chromosome:IBSC_v2:chr5H:8597589:8736... 295 3e-78

EG:HORVU5Hr1G005180.4 cdna chromosome:IBSC_v2:chr5H:8598500:8736... 295 3e-78

EG:HORVU5Hr1G005180.2 cdna chromosome:IBSC_v2:chr5H:8597589:8599... 295 3e-78

EG:HORVU5Hr1G005310.3 cdna chromosome:IBSC_v2:chr5H:8830987:8832... 287 7e-76

EG:HORVU5Hr1G005310.1 cdna chromosome:IBSC_v2:chr5H:8830987:8832... 287 7e-76

EG:HORVU5Hr1G005310.2 cdna chromosome:IBSC_v2:chr5H:8830987:8832... 287 7e-76

EG:HORVU5Hr1G005180.8 cdna chromosome:IBSC_v2:chr5H:8736390:8737... 210 1e-52

EG:HORVU5Hr1G005180.7 cdna chromosome:IBSC_v2:chr5H:8614572:8736... 210 1e-52

EG:HORVU5Hr1G051960.1 cdna chromosome:IBSC_v2:chr5H:406424994:40... 190 1e-46

EG:HORVU5Hr1G051950.1 cdna chromosome:IBSC_v2:chr5H:406424295:40... 159 4e-37

EG:HORVU5Hr1G005310.4 cdna chromosome:IBSC_v2:chr5H:8831045:8831... 109 4e-22

EG:HORVU5Hr1G005510.1 cdna chromosome:IBSC_v2:chr5H:8927171:8927... 69.9 3e-10

EG:HORVU5Hr1G005240.1 cdna chromosome:IBSC_v2:chr5H:8650476:8651... 67.9 1e-09

EG:HORVU5Hr1G005190.1 cdna chromosome:IBSC_v2:chr5H:8603703:8604... 54.0 2e-05

EG:HORVU3Hr1G012050.1 cdna chromosome:IBSC_v2:chr3H:26279584:262... 50.1 3e-04

EG:HORVU3Hr1G012050.3 cdna chromosome:IBSC_v2:chr3H:26279787:262... 50.1 3e-04

EG:HORVU3Hr1G012050.2 cdna chromosome:IBSC_v2:chr3H:26279784:262... 50.1 3e-04

EG:HORVU4Hr1G002640.1 cdna chromosome:IBSC_v2:chr4H:5066553:5067... 50.1 3e-04

EG:HORVU5Hr1G017530.1 cdna chromosome:IBSC_v2:chr5H:67689233:676... 50.1 3e-04

EG:HORVU5Hr1G021560.1 cdna chromosome:IBSC_v2:chr5H:105345418:10... 46.1 0.004

EG:HORVU4Hr1G002650.1 cdna chromosome:IBSC_v2:chr4H:5071114:5074... 46.1 0.004

EG:HORVU4Hr1G002650.2 cdna chromosome:IBSC_v2:chr4H:5073727:5074... 46.1 0.004

EG:HORVU5Hr1G005290.2 cdna chromosome:IBSC_v2:chr5H:8764462:8764... 40.1 0.27

EG:HORVU5Hr1G005290.1 cdna chromosome:IBSC_v2:chr5H:8764295:8765... 40.1 0.27

EG:HORVU5Hr1G083260.4 cdna chromosome:IBSC_v2:chr5H:569572888:56... 40.1 0.27

EG:HORVU5Hr1G083260.3 cdna chromosome:IBSC_v2:chr5H:569572084:56... 40.1 0.27

EG:HORVU3Hr1G002210.10 cdna chromosome:IBSC_v2:chr3H:4630613:463... 38.2 1.1

EG:HORVU3Hr1G002210.9 cdna chromosome:IBSC_v2:chr3H:4630604:4632... 38.2 1.1

EG:HORVU3Hr1G002210.8 cdna chromosome:IBSC_v2:chr3H:4630564:4632... 38.2 1.1

EG:HORVU4Hr1G063690.7 cdna chromosome:IBSC_v2:chr4H:532860843:53... 38.2 1.1

EG:HORVU4Hr1G063690.2 cdna chromosome:IBSC_v2:chr4H:532859440:53... 38.2 1.1

EG:HORVU4Hr1G063690.9 cdna chromosome:IBSC_v2:chr4H:532860915:53... 38.2 1.1

EG:HORVU4Hr1G063690.6 cdna chromosome:IBSC_v2:chr4H:532860840:53... 38.2 1.1

EG:HORVU4Hr1G063690.8 cdna chromosome:IBSC_v2:chr4H:532860846:53... 38.2 1.1

EG:HORVU4Hr1G063690.1 cdna chromosome:IBSC_v2:chr4H:532859323:53... 38.2 1.1

EG:HORVU4Hr1G063690.3 cdna chromosome:IBSC_v2:chr4H:532859502:53... 38.2 1.1

EG:HORVU4Hr1G063690.5 cdna chromosome:IBSC_v2:chr4H:532860834:53... 38.2 1.1

EG:HORVU4Hr1G063730.3 cdna chromosome:IBSC_v2:chr4H:533491850:53... 38.2 1.1

EG:HORVU4Hr1G063730.5 cdna chromosome:IBSC_v2:chr4H:533492184:53... 38.2 1.1

EG:HORVU4Hr1G063730.1 cdna chromosome:IBSC_v2:chr4H:533491696:53... 38.2 1.1

>EG:HORVU5Hr1G005180.6 cdna chromosome:IBSC_v2:chr5H:8614116:8615298:-1 gene:HORVU5Hr1G005180

gene_biotype:protein_coding transcript_biotype:protein_coding

Length=1183

Score = 1425 bits (719), Expect = 0.0

Identities = 719/719 (100%), Gaps = 0/719 (0%)

Strand=Plus/Plus

Query 1 ATGGCGTCCTCTCGTGTTGTCTACCTCCTGGCCGGTCTACTCCTAGCCGCCCTCGCCGCC 60

||||||||||||||||||||||||||||||||||||||||||||||||||||||||||||

Sbjct 205 ATGGCGTCCTCTCGTGTTGTCTACCTCCTGGCCGGTCTACTCCTAGCCGCCCTCGCCGCC 264

Query 61 ACCACAGACGCGGCCACCATCACCGTCGTCAACCGGTGCTCCTACACGGTGTGGCCGGGC 120

||||||||||||||||||||||||||||||||||||||||||||||||||||||||||||

Sbjct 265 ACCACAGACGCGGCCACCATCACCGTCGTCAACCGGTGCTCCTACACGGTGTGGCCGGGC 324

Query 121 GCGCTCCCGGGTGGCGGTGTGCGTCTCGACCCAGGCCAGTCGTGGGCGCTGAACATGCCC 180

||||||||||||||||||||||||||||||||||||||||||||||||||||||||||||

Sbjct 325 GCGCTCCCGGGTGGCGGTGTGCGTCTCGACCCAGGCCAGTCGTGGGCGCTGAACATGCCC 384

Query 181 GCGGGTACCGCGGGCGCCAGGGTGTGGCCGCGCACCGGGTGCACCTTCGACGGCAGCGGC 240

||||||||||||||||||||||||||||||||||||||||||||||||||||||||||||

Sbjct 385 GCGGGTACCGCGGGCGCCAGGGTGTGGCCGCGCACCGGGTGCACCTTCGACGGCAGCGGC 444

Query 241 CGCGGCCGGTGCATCACCGGCGACTGCAACGGTGTGCTGGCATGCCGGGTGTCCGGCCAG 300

||||||||||||||||||||||||||||||||||||||||||||||||||||||||||||

Sbjct 445 CGCGGCCGGTGCATCACCGGCGACTGCAACGGTGTGCTGGCATGCCGGGTGTCCGGCCAG 504

Query 301 CAGCCGACCACGCTGGCCGAGTACACCCTGGGCCAGGGCGCGAACAAGGACTTCTTCGAC 360

||||||||||||||||||||||||||||||||||||||||||||||||||||||||||||

Sbjct 505 CAGCCGACCACGCTGGCCGAGTACACCCTGGGCCAGGGCGCGAACAAGGACTTCTTCGAC 564

Query 361 CTGTCCGTCATCGACGGGTTCAACGTGCCGATGAGCTTCGAGCCCGTGGGAGGGTGCCGT 420

||||||||||||||||||||||||||||||||||||||||||||||||||||||||||||

Sbjct 565 CTGTCCGTCATCGACGGGTTCAACGTGCCGATGAGCTTCGAGCCCGTGGGAGGGTGCCGT 624

Query 421 GCTGCGCGGTGCGCCACGGACATCACCAAGGATTGCCTCAAGGAGCTGCAGGTGCCGGGA 480

||||||||||||||||||||||||||||||||||||||||||||||||||||||||||||

Sbjct 625 GCTGCGCGGTGCGCCACGGACATCACCAAGGATTGCCTCAAGGAGCTGCAGGTGCCGGGA 684

Query 481 GGGTGCGCGAGCGCGTGCGGCAAGTTCGGCGGCGACACCTACTGCTGCCGTGGCCAGTTT 540

||||||||||||||||||||||||||||||||||||||||||||||||||||||||||||

Sbjct 685 GGGTGCGCGAGCGCGTGCGGCAAGTTCGGCGGCGACACCTACTGCTGCCGTGGCCAGTTT 744

Query 541 GAGCACAACTGCCCGCCGACCAACTACTCAATGTTCTTCAAGGGCAAATGCCCCGACGCC 600

||||||||||||||||||||||||||||||||||||||||||||||||||||||||||||

Sbjct 745 GAGCACAACTGCCCGCCGACCAACTACTCAATGTTCTTCAAGGGCAAATGCCCCGACGCC 804

Query 601 TATAGCTATGCGAAGGACGACCAGACCAGCACCTTCACCTGCCCTGCCGGAACCAACTAC 660

||||||||||||||||||||||||||||||||||||||||||||||||||||||||||||

Sbjct 805 TATAGCTATGCGAAGGACGACCAGACCAGCACCTTCACCTGCCCTGCCGGAACCAACTAC 864

Query 661 CAGATCGTCCTCTGCCCTTAGATCAATATCAGTCAATTACTTGGGTCAATAAGGAATAA 719

|||||||||||||||||||||||||||||||||||||||||||||||||||||||||||

Sbjct 865 CAGATCGTCCTCTGCCCTTAGATCAATATCAGTCAATTACTTGGGTCAATAAGGAATAA 923

>EG:HORVU5Hr1G051970.4 cdna chromosome:IBSC_v2:chr5H:406435574:406436533:1 gene:HORVU5Hr1G051970

gene_biotype:protein_coding transcript_biotype:protein_coding

Length=889

Score = 579 bits (292), Expect = 1e-163

Identities = 361/384 (94%), Gaps = 0/384 (0%)

Strand=Plus/Plus

Query 24 CCTCCTGGCCGGTCTACTCCTAGCCGCCCTCGCCGCCACCACAGACGCGGCCACCATCAC 83

||||||||||||||| ||||| |||||||||||||| | |||||||||||||||||||||

Sbjct 80 CCTCCTGGCCGGTCTCCTCCTTGCCGCCCTCGCCGCAAGCACAGACGCGGCCACCATCAC 139

Query 84 CGTCGTCAACCGGTGCTCCTACACGGTGTGGCCGGGCGCGCTCCCGGGTGGCGGTGTGCG 143

||||||||||||||||||||||||||||||||||||||||||||| |||||||| |||||

Sbjct 140 CGTCGTCAACCGGTGCTCCTACACGGTGTGGCCGGGCGCGCTCCCAGGTGGCGGCGTGCG 199

Query 144 TCTCGACCCAGGCCAGTCGTGGGCGCTGAACATGCCCGCGGGTACCGCGGGCGCCAGGGT 203

|||||||||||||||||||||||| ||||||||||| || || |||||||||||||||||

Sbjct 200 TCTCGACCCAGGCCAGTCGTGGGCTCTGAACATGCCGGCCGGCACCGCGGGCGCCAGGGT 259

Query 204 GTGGCCGCGCACCGGGTGCACCTTCGACGGCAGCGGCCGCGGCCGGTGCATCACCGGCGA 263

|||||||||||| |||||||||||||||||||||||||| ||||||||||||||||| ||

Sbjct 260 GTGGCCGCGCACGGGGTGCACCTTCGACGGCAGCGGCCGTGGCCGGTGCATCACCGGAGA 319

Query 264 CTGCAACGGTGTGCTGGCATGCCGGGTGTCCGGCCAGCAGCCGACCACGCTGGCCGAGTA 323

|||| ||| | |||||||||||| ||||||||||||||||| |||||| ||||||||||

Sbjct 320 CTGCGGCGGCGCGCTGGCATGCCGCGTGTCCGGCCAGCAGCCCACCACGTTGGCCGAGTA 379

Query 324 CACCCTGGGCCAGGGCGCGAACAAGGACTTCTTCGACCTGTCCGTCATCGACGGGTTCAA 383

||||||||| ||||||||||||||||||||||||||||||||||||||||||||||||||

Sbjct 380 CACCCTGGGACAGGGCGCGAACAAGGACTTCTTCGACCTGTCCGTCATCGACGGGTTCAA 439

Query 384 CGTGCCGATGAGCTTCGAGCCCGT 407

||| || |||||||||||||||||

Sbjct 440 CGTTCCCATGAGCTTCGAGCCCGT 463

Score = 442 bits (223), Expect = 2e-122

Identities = 256/267 (96%), Gaps = 0/267 (0%)

Strand=Plus/Plus

Query 414 GTGCCGTGCTGCGCGGTGCGCCACGGACATCACCAAGGATTGCCTCAAGGAGCTGCAGGT 473

|||||||||||||||||| |||||||||||||||||||| ||||||||||||||||||||

Sbjct 473 GTGCCGTGCTGCGCGGTGTGCCACGGACATCACCAAGGAGTGCCTCAAGGAGCTGCAGGT 532

Query 474 GCCGGGAGGGTGCGCGAGCGCGTGCGGCAAGTTCGGCGGCGACACCTACTGCTGCCGTGG 533

||||||||||||||||||||||||||||||||||||||||||||||||||||||||| ||

Sbjct 533 GCCGGGAGGGTGCGCGAGCGCGTGCGGCAAGTTCGGCGGCGACACCTACTGCTGCCGGGG 592

Query 534 CCAGTTTGAGCACAACTGCCCGCCGACCAACTACTCAATGTTCTTCAAGGGCAAATGCCC 593

|||||| ||||||||||| |||||||||||||| || | ||||||||||||||| |||||

Sbjct 593 CCAGTTCGAGCACAACTGTCCGCCGACCAACTATTCGAAGTTCTTCAAGGGCAAGTGCCC 652

Query 594 CGACGCCTATAGCTATGCGAAGGACGACCAGACCAGCACCTTCACCTGCCCTGCCGGAAC 653

|||||||||||||||||| |||||||||||||||||||||||||||||||||||||||||

Sbjct 653 CGACGCCTATAGCTATGCCAAGGACGACCAGACCAGCACCTTCACCTGCCCTGCCGGAAC 712

Query 654 CAACTACCAGATCGTCCTCTGCCCTTA 680

||||||||||||||||||||||||||

Sbjct 713 AAACTACCAGATCGTCCTCTGCCCTTA 739

>EG:HORVU5Hr1G051970.7 cdna chromosome:IBSC_v2:chr5H:406435630:406436415:1 gene:HORVU5Hr1G051970

gene_biotype:protein_coding transcript_biotype:protein_coding

Length=715

Score = 579 bits (292), Expect = 1e-163

Identities = 361/384 (94%), Gaps = 0/384 (0%)

Strand=Plus/Plus

Query 24 CCTCCTGGCCGGTCTACTCCTAGCCGCCCTCGCCGCCACCACAGACGCGGCCACCATCAC 83

||||||||||||||| ||||| |||||||||||||| | |||||||||||||||||||||

Sbjct 24 CCTCCTGGCCGGTCTCCTCCTTGCCGCCCTCGCCGCAAGCACAGACGCGGCCACCATCAC 83

Query 84 CGTCGTCAACCGGTGCTCCTACACGGTGTGGCCGGGCGCGCTCCCGGGTGGCGGTGTGCG 143

||||||||||||||||||||||||||||||||||||||||||||| |||||||| |||||

Sbjct 84 CGTCGTCAACCGGTGCTCCTACACGGTGTGGCCGGGCGCGCTCCCAGGTGGCGGCGTGCG 143

Query 144 TCTCGACCCAGGCCAGTCGTGGGCGCTGAACATGCCCGCGGGTACCGCGGGCGCCAGGGT 203

|||||||||||||||||||||||| ||||||||||| || || |||||||||||||||||

Sbjct 144 TCTCGACCCAGGCCAGTCGTGGGCTCTGAACATGCCGGCCGGCACCGCGGGCGCCAGGGT 203

Query 204 GTGGCCGCGCACCGGGTGCACCTTCGACGGCAGCGGCCGCGGCCGGTGCATCACCGGCGA 263

|||||||||||| |||||||||||||||||||||||||| ||||||||||||||||| ||

Sbjct 204 GTGGCCGCGCACGGGGTGCACCTTCGACGGCAGCGGCCGTGGCCGGTGCATCACCGGAGA 263

Query 264 CTGCAACGGTGTGCTGGCATGCCGGGTGTCCGGCCAGCAGCCGACCACGCTGGCCGAGTA 323

|||| ||| | |||||||||||| ||||||||||||||||| |||||| ||||||||||

Sbjct 264 CTGCGGCGGCGCGCTGGCATGCCGCGTGTCCGGCCAGCAGCCCACCACGTTGGCCGAGTA 323

Query 324 CACCCTGGGCCAGGGCGCGAACAAGGACTTCTTCGACCTGTCCGTCATCGACGGGTTCAA 383

||||||||| ||||||||||||||||||||||||||||||||||||||||||||||||||

Sbjct 324 CACCCTGGGACAGGGCGCGAACAAGGACTTCTTCGACCTGTCCGTCATCGACGGGTTCAA 383

Query 384 CGTGCCGATGAGCTTCGAGCCCGT 407

||| || |||||||||||||||||

Sbjct 384 CGTTCCCATGAGCTTCGAGCCCGT 407

Score = 442 bits (223), Expect = 2e-122

Identities = 256/267 (96%), Gaps = 0/267 (0%)

Strand=Plus/Plus

Query 414 GTGCCGTGCTGCGCGGTGCGCCACGGACATCACCAAGGATTGCCTCAAGGAGCTGCAGGT 473

|||||||||||||||||| |||||||||||||||||||| ||||||||||||||||||||

Sbjct 417 GTGCCGTGCTGCGCGGTGTGCCACGGACATCACCAAGGAGTGCCTCAAGGAGCTGCAGGT 476

Query 474 GCCGGGAGGGTGCGCGAGCGCGTGCGGCAAGTTCGGCGGCGACACCTACTGCTGCCGTGG 533

||||||||||||||||||||||||||||||||||||||||||||||||||||||||| ||

Sbjct 477 GCCGGGAGGGTGCGCGAGCGCGTGCGGCAAGTTCGGCGGCGACACCTACTGCTGCCGGGG 536

Query 534 CCAGTTTGAGCACAACTGCCCGCCGACCAACTACTCAATGTTCTTCAAGGGCAAATGCCC 593

|||||| ||||||||||| |||||||||||||| || | ||||||||||||||| |||||

Sbjct 537 CCAGTTCGAGCACAACTGTCCGCCGACCAACTATTCGAAGTTCTTCAAGGGCAAGTGCCC 596

Query 594 CGACGCCTATAGCTATGCGAAGGACGACCAGACCAGCACCTTCACCTGCCCTGCCGGAAC 653

|||||||||||||||||| |||||||||||||||||||||||||||||||||||||||||

Sbjct 597 CGACGCCTATAGCTATGCCAAGGACGACCAGACCAGCACCTTCACCTGCCCTGCCGGAAC 656

Query 654 CAACTACCAGATCGTCCTCTGCCCTTA 680

||||||||||||||||||||||||||

Sbjct 657 AAACTACCAGATCGTCCTCTGCCCTTA 683

>EG:HORVU5Hr1G051970.6 cdna chromosome:IBSC_v2:chr5H:406435630:406436384:1 gene:HORVU5Hr1G051970

gene_biotype:protein_coding transcript_biotype:protein_coding

Length=684

Score = 579 bits (292), Expect = 1e-163

Identities = 361/384 (94%), Gaps = 0/384 (0%)

Strand=Plus/Plus

Query 24 CCTCCTGGCCGGTCTACTCCTAGCCGCCCTCGCCGCCACCACAGACGCGGCCACCATCAC 83

||||||||||||||| ||||| |||||||||||||| | |||||||||||||||||||||

Sbjct 24 CCTCCTGGCCGGTCTCCTCCTTGCCGCCCTCGCCGCAAGCACAGACGCGGCCACCATCAC 83

Query 84 CGTCGTCAACCGGTGCTCCTACACGGTGTGGCCGGGCGCGCTCCCGGGTGGCGGTGTGCG 143

||||||||||||||||||||||||||||||||||||||||||||| |||||||| |||||

Sbjct 84 CGTCGTCAACCGGTGCTCCTACACGGTGTGGCCGGGCGCGCTCCCAGGTGGCGGCGTGCG 143

Query 144 TCTCGACCCAGGCCAGTCGTGGGCGCTGAACATGCCCGCGGGTACCGCGGGCGCCAGGGT 203

|||||||||||||||||||||||| ||||||||||| || || |||||||||||||||||

Sbjct 144 TCTCGACCCAGGCCAGTCGTGGGCTCTGAACATGCCGGCCGGCACCGCGGGCGCCAGGGT 203

Query 204 GTGGCCGCGCACCGGGTGCACCTTCGACGGCAGCGGCCGCGGCCGGTGCATCACCGGCGA 263

|||||||||||| |||||||||||||||||||||||||| ||||||||||||||||| ||

Sbjct 204 GTGGCCGCGCACGGGGTGCACCTTCGACGGCAGCGGCCGTGGCCGGTGCATCACCGGAGA 263

Query 264 CTGCAACGGTGTGCTGGCATGCCGGGTGTCCGGCCAGCAGCCGACCACGCTGGCCGAGTA 323

|||| ||| | |||||||||||| ||||||||||||||||| |||||| ||||||||||

Sbjct 264 CTGCGGCGGCGCGCTGGCATGCCGCGTGTCCGGCCAGCAGCCCACCACGTTGGCCGAGTA 323

Query 324 CACCCTGGGCCAGGGCGCGAACAAGGACTTCTTCGACCTGTCCGTCATCGACGGGTTCAA 383

||||||||| ||||||||||||||||||||||||||||||||||||||||||||||||||

Sbjct 324 CACCCTGGGACAGGGCGCGAACAAGGACTTCTTCGACCTGTCCGTCATCGACGGGTTCAA 383

Query 384 CGTGCCGATGAGCTTCGAGCCCGT 407

||| || |||||||||||||||||

Sbjct 384 CGTTCCCATGAGCTTCGAGCCCGT 407

Score = 442 bits (223), Expect = 2e-122

Identities = 256/267 (96%), Gaps = 0/267 (0%)

Strand=Plus/Plus

Query 414 GTGCCGTGCTGCGCGGTGCGCCACGGACATCACCAAGGATTGCCTCAAGGAGCTGCAGGT 473

|||||||||||||||||| |||||||||||||||||||| ||||||||||||||||||||

Sbjct 417 GTGCCGTGCTGCGCGGTGTGCCACGGACATCACCAAGGAGTGCCTCAAGGAGCTGCAGGT 476

Query 474 GCCGGGAGGGTGCGCGAGCGCGTGCGGCAAGTTCGGCGGCGACACCTACTGCTGCCGTGG 533

||||||||||||||||||||||||||||||||||||||||||||||||||||||||| ||

Sbjct 477 GCCGGGAGGGTGCGCGAGCGCGTGCGGCAAGTTCGGCGGCGACACCTACTGCTGCCGGGG 536

Query 534 CCAGTTTGAGCACAACTGCCCGCCGACCAACTACTCAATGTTCTTCAAGGGCAAATGCCC 593

|||||| ||||||||||| |||||||||||||| || | ||||||||||||||| |||||

Sbjct 537 CCAGTTCGAGCACAACTGTCCGCCGACCAACTATTCGAAGTTCTTCAAGGGCAAGTGCCC 596

Query 594 CGACGCCTATAGCTATGCGAAGGACGACCAGACCAGCACCTTCACCTGCCCTGCCGGAAC 653

|||||||||||||||||| |||||||||||||||||||||||||||||||||||||||||

Sbjct 597 CGACGCCTATAGCTATGCCAAGGACGACCAGACCAGCACCTTCACCTGCCCTGCCGGAAC 656

Query 654 CAACTACCAGATCGTCCTCTGCCCTTA 680

||||||||||||||||||||||||||

Sbjct 657 AAACTACCAGATCGTCCTCTGCCCTTA 683

>EG:HORVU5Hr1G051970.5 cdna chromosome:IBSC_v2:chr5H:406435615:406436443:1 gene:HORVU5Hr1G051970

gene_biotype:protein_coding transcript_biotype:protein_coding

Length=758

Score = 571 bits (288), Expect = 3e-161

Identities = 360/384 (94%), Gaps = 0/384 (0%)

Strand=Plus/Plus

Query 24 CCTCCTGGCCGGTCTACTCCTAGCCGCCCTCGCCGCCACCACAGACGCGGCCACCATCAC 83

||||||||||||||| ||||| |||||||||||||| | |||||||||||||||||||||

Sbjct 39 CCTCCTGGCCGGTCTCCTCCTTGCCGCCCTCGCCGCAAGCACAGACGCGGCCACCATCAC 98

Query 84 CGTCGTCAACCGGTGCTCCTACACGGTGTGGCCGGGCGCGCTCCCGGGTGGCGGTGTGCG 143

||||||||||||||||||||||||||||||||||||||||||||| |||||||| |||||

Sbjct 99 CGTCGTCAACCGGTGCTCCTACACGGTGTGGCCGGGCGCGCTCCCAGGTGGCGGCGTGCG 158

Query 144 TCTCGACCCAGGCCAGTCGTGGGCGCTGAACATGCCCGCGGGTACCGCGGGCGCCAGGGT 203

|||||||||||||||||||||||| ||||||||||| || || ||||||| |||||||||

Sbjct 159 TCTCGACCCAGGCCAGTCGTGGGCTCTGAACATGCCGGCCGGCACCGCGGTCGCCAGGGT 218

Query 204 GTGGCCGCGCACCGGGTGCACCTTCGACGGCAGCGGCCGCGGCCGGTGCATCACCGGCGA 263

|||||||||||| |||||||||||||||||||||||||| ||||||||||||||||| ||

Sbjct 219 GTGGCCGCGCACGGGGTGCACCTTCGACGGCAGCGGCCGTGGCCGGTGCATCACCGGAGA 278

Query 264 CTGCAACGGTGTGCTGGCATGCCGGGTGTCCGGCCAGCAGCCGACCACGCTGGCCGAGTA 323

|||| ||| | |||||||||||| ||||||||||||||||| |||||| ||||||||||

Sbjct 279 CTGCGGCGGCGCGCTGGCATGCCGCGTGTCCGGCCAGCAGCCCACCACGTTGGCCGAGTA 338

Query 324 CACCCTGGGCCAGGGCGCGAACAAGGACTTCTTCGACCTGTCCGTCATCGACGGGTTCAA 383

||||||||| ||||||||||||||||||||||||||||||||||||||||||||||||||

Sbjct 339 CACCCTGGGACAGGGCGCGAACAAGGACTTCTTCGACCTGTCCGTCATCGACGGGTTCAA 398

Query 384 CGTGCCGATGAGCTTCGAGCCCGT 407

||| || |||||||||||||||||

Sbjct 399 CGTTCCCATGAGCTTCGAGCCCGT 422

Score = 442 bits (223), Expect = 2e-122

Identities = 256/267 (96%), Gaps = 0/267 (0%)

Strand=Plus/Plus

Query 414 GTGCCGTGCTGCGCGGTGCGCCACGGACATCACCAAGGATTGCCTCAAGGAGCTGCAGGT 473

|||||||||||||||||| |||||||||||||||||||| ||||||||||||||||||||

Sbjct 432 GTGCCGTGCTGCGCGGTGTGCCACGGACATCACCAAGGAGTGCCTCAAGGAGCTGCAGGT 491

Query 474 GCCGGGAGGGTGCGCGAGCGCGTGCGGCAAGTTCGGCGGCGACACCTACTGCTGCCGTGG 533

||||||||||||||||||||||||||||||||||||||||||||||||||||||||| ||

Sbjct 492 GCCGGGAGGGTGCGCGAGCGCGTGCGGCAAGTTCGGCGGCGACACCTACTGCTGCCGGGG 551

Query 534 CCAGTTTGAGCACAACTGCCCGCCGACCAACTACTCAATGTTCTTCAAGGGCAAATGCCC 593

|||||| ||||||||||| |||||||||||||| || | ||||||||||||||| |||||

Sbjct 552 CCAGTTCGAGCACAACTGTCCGCCGACCAACTATTCGAAGTTCTTCAAGGGCAAGTGCCC 611

Query 594 CGACGCCTATAGCTATGCGAAGGACGACCAGACCAGCACCTTCACCTGCCCTGCCGGAAC 653

|||||||||||||||||| |||||||||||||||||||||||||||||||||||||||||

Sbjct 612 CGACGCCTATAGCTATGCCAAGGACGACCAGACCAGCACCTTCACCTGCCCTGCCGGAAC 671

Query 654 CAACTACCAGATCGTCCTCTGCCCTTA 680

||||||||||||||||||||||||||

Sbjct 672 AAACTACCAGATCGTCCTCTGCCCTTA 698

>EG:HORVU5Hr1G051970.1 cdna chromosome:IBSC_v2:chr5H:406435550:406436444:1 gene:HORVU5Hr1G051970

gene_biotype:protein_coding transcript_biotype:protein_coding

Length=895

Score = 442 bits (223), Expect = 2e-122

Identities = 256/267 (96%), Gaps = 0/267 (0%)

Strand=Plus/Plus

Query 414 GTGCCGTGCTGCGCGGTGCGCCACGGACATCACCAAGGATTGCCTCAAGGAGCTGCAGGT 473

|||||||||||||||||| |||||||||||||||||||| ||||||||||||||||||||

Sbjct 568 GTGCCGTGCTGCGCGGTGTGCCACGGACATCACCAAGGAGTGCCTCAAGGAGCTGCAGGT 627

Query 474 GCCGGGAGGGTGCGCGAGCGCGTGCGGCAAGTTCGGCGGCGACACCTACTGCTGCCGTGG 533

||||||||||||||||||||||||||||||||||||||||||||||||||||||||| ||

Sbjct 628 GCCGGGAGGGTGCGCGAGCGCGTGCGGCAAGTTCGGCGGCGACACCTACTGCTGCCGGGG 687

Query 534 CCAGTTTGAGCACAACTGCCCGCCGACCAACTACTCAATGTTCTTCAAGGGCAAATGCCC 593

|||||| ||||||||||| |||||||||||||| || | ||||||||||||||| |||||

Sbjct 688 CCAGTTCGAGCACAACTGTCCGCCGACCAACTATTCGAAGTTCTTCAAGGGCAAGTGCCC 747

Query 594 CGACGCCTATAGCTATGCGAAGGACGACCAGACCAGCACCTTCACCTGCCCTGCCGGAAC 653

|||||||||||||||||| |||||||||||||||||||||||||||||||||||||||||

Sbjct 748 CGACGCCTATAGCTATGCCAAGGACGACCAGACCAGCACCTTCACCTGCCCTGCCGGAAC 807

Query 654 CAACTACCAGATCGTCCTCTGCCCTTA 680

||||||||||||||||||||||||||

Sbjct 808 AAACTACCAGATCGTCCTCTGCCCTTA 834

Score = 369 bits (186), Expect = 2e-100

Identities = 228/242 (94%), Gaps = 0/242 (0%)

Strand=Plus/Plus

Query 24 CCTCCTGGCCGGTCTACTCCTAGCCGCCCTCGCCGCCACCACAGACGCGGCCACCATCAC 83

||||||||||||||| ||||| |||||||||||||| | |||||||||||||||||||||

Sbjct 104 CCTCCTGGCCGGTCTCCTCCTTGCCGCCCTCGCCGCAAGCACAGACGCGGCCACCATCAC 163

Query 84 CGTCGTCAACCGGTGCTCCTACACGGTGTGGCCGGGCGCGCTCCCGGGTGGCGGTGTGCG 143

||||||||||||||||||||||||||||||||||||||||||||| |||||||| |||||

Sbjct 164 CGTCGTCAACCGGTGCTCCTACACGGTGTGGCCGGGCGCGCTCCCAGGTGGCGGCGTGCG 223

Query 144 TCTCGACCCAGGCCAGTCGTGGGCGCTGAACATGCCCGCGGGTACCGCGGGCGCCAGGGT 203

|||||||||||||||||||||||| ||||||||||| || || |||||||||||||||||

Sbjct 224 TCTCGACCCAGGCCAGTCGTGGGCTCTGAACATGCCGGCCGGCACCGCGGGCGCCAGGGT 283

Query 204 GTGGCCGCGCACCGGGTGCACCTTCGACGGCAGCGGCCGCGGCCGGTGCATCACCGGCGA 263

|||||||||||| |||||||||||||||||||||||||| |||||| |||||||||| ||

Sbjct 284 GTGGCCGCGCACGGGGTGCACCTTCGACGGCAGCGGCCGGGGCCGGGGCATCACCGGAGA 343

Query 264 CT 265

||

Sbjct 344 CT 345

Score = 319 bits (161), Expect = 2e-85

Identities = 200/213 (94%), Gaps = 0/213 (0%)

Strand=Plus/Plus

Query 195 CGCCAGGGTGTGGCCGCGCACCGGGTGCACCTTCGACGGCAGCGGCCGCGGCCGGTGCAT 254

||||||||||||||||||||| |||||||||||||||||||||||||| |||||||||||

Sbjct 346 CGCCAGGGTGTGGCCGCGCACGGGGTGCACCTTCGACGGCAGCGGCCGTGGCCGGTGCAT 405

Query 255 CACCGGCGACTGCAACGGTGTGCTGGCATGCCGGGTGTCCGGCCAGCAGCCGACCACGCT 314

|||||| |||||| ||| | |||||||||||| ||||||||||||||||| |||||| |

Sbjct 406 CACCGGAGACTGCGGCGGCGCGCTGGCATGCCGCGTGTCCGGCCAGCAGCCCACCACGTT 465

Query 315 GGCCGAGTACACCCTGGGCCAGGGCGCGAACAAGGACTTCTTCGACCTGTCCGTCATCGA 374

|||||||||||||||||| |||||||||||||||||||||||||||||||||||||||||

Sbjct 466 GGCCGAGTACACCCTGGGACAGGGCGCGAACAAGGACTTCTTCGACCTGTCCGTCATCGA 525

Query 375 CGGGTTCAACGTGCCGATGAGCTTCGAGCCCGT 407

|||||||||||| || |||||||||||||||||

Sbjct 526 CGGGTTCAACGTTCCCATGAGCTTCGAGCCCGT 558

>EG:HORVU5Hr1G051970.3 cdna chromosome:IBSC_v2:chr5H:406435574:406436446:1 gene:HORVU5Hr1G051970

gene_biotype:protein_coding transcript_biotype:protein_coding

Length=806

Score = 442 bits (223), Expect = 2e-122

Identities = 256/267 (96%), Gaps = 0/267 (0%)

Strand=Plus/Plus

Query 414 GTGCCGTGCTGCGCGGTGCGCCACGGACATCACCAAGGATTGCCTCAAGGAGCTGCAGGT 473

|||||||||||||||||| |||||||||||||||||||| ||||||||||||||||||||

Sbjct 477 GTGCCGTGCTGCGCGGTGTGCCACGGACATCACCAAGGAGTGCCTCAAGGAGCTGCAGGT 536

Query 474 GCCGGGAGGGTGCGCGAGCGCGTGCGGCAAGTTCGGCGGCGACACCTACTGCTGCCGTGG 533

||||||||||||||||||||||||||||||||||||||||||||||||||||||||| ||

Sbjct 537 GCCGGGAGGGTGCGCGAGCGCGTGCGGCAAGTTCGGCGGCGACACCTACTGCTGCCGGGG 596

Query 534 CCAGTTTGAGCACAACTGCCCGCCGACCAACTACTCAATGTTCTTCAAGGGCAAATGCCC 593

|||||| ||||||||||| |||||||||||||| || | ||||||||||||||| |||||

Sbjct 597 CCAGTTCGAGCACAACTGTCCGCCGACCAACTATTCGAAGTTCTTCAAGGGCAAGTGCCC 656

Query 594 CGACGCCTATAGCTATGCGAAGGACGACCAGACCAGCACCTTCACCTGCCCTGCCGGAAC 653

|||||||||||||||||| |||||||||||||||||||||||||||||||||||||||||

Sbjct 657 CGACGCCTATAGCTATGCCAAGGACGACCAGACCAGCACCTTCACCTGCCCTGCCGGAAC 716

Query 654 CAACTACCAGATCGTCCTCTGCCCTTA 680

||||||||||||||||||||||||||

Sbjct 717 AAACTACCAGATCGTCCTCTGCCCTTA 743

Score = 311 bits (157), Expect = 5e-83

Identities = 190/201 (95%), Gaps = 0/201 (0%)

Strand=Plus/Plus

Query 24 CCTCCTGGCCGGTCTACTCCTAGCCGCCCTCGCCGCCACCACAGACGCGGCCACCATCAC 83

||||||||||||||| ||||| |||||||||||||| | |||||||||||||||||||||

Sbjct 80 CCTCCTGGCCGGTCTCCTCCTTGCCGCCCTCGCCGCAAGCACAGACGCGGCCACCATCAC 139

Query 84 CGTCGTCAACCGGTGCTCCTACACGGTGTGGCCGGGCGCGCTCCCGGGTGGCGGTGTGCG 143

||||||||||||||||||||||||||||||||||||||||||||| |||||||| |||||

Sbjct 140 CGTCGTCAACCGGTGCTCCTACACGGTGTGGCCGGGCGCGCTCCCAGGTGGCGGCGTGCG 199

Query 144 TCTCGACCCAGGCCAGTCGTGGGCGCTGAACATGCCCGCGGGTACCGCGGGCGCCAGGGT 203

|||||||||||||||||||||||| ||||||||||| || || |||||||||||||||||

Sbjct 200 TCTCGACCCAGGCCAGTCGTGGGCTCTGAACATGCCGGCCGGCACCGCGGGCGCCAGGGT 259

Query 204 GTGGCCGCGCACCGGGTGCAC 224

|||||||||||| ||||||||

Sbjct 260 GTGGCCGCGCACGGGGTGCAC 280

Score = 276 bits (139), Expect = 3e-72

Identities = 175/187 (94%), Gaps = 0/187 (0%)

Strand=Plus/Plus

Query 221 GCACCTTCGACGGCAGCGGCCGCGGCCGGTGCATCACCGGCGACTGCAACGGTGTGCTGG 280

|||||||||||||||||||||| ||||||||||||||||| |||||| ||| | |||||

Sbjct 281 GCACCTTCGACGGCAGCGGCCGTGGCCGGTGCATCACCGGAGACTGCGGCGGCGCGCTGG 340

Query 281 CATGCCGGGTGTCCGGCCAGCAGCCGACCACGCTGGCCGAGTACACCCTGGGCCAGGGCG 340

||||||| ||||||||||||||||| |||||| ||||||||||||||||||| |||||||

Sbjct 341 CATGCCGCGTGTCCGGCCAGCAGCCCACCACGTTGGCCGAGTACACCCTGGGACAGGGCG 400

Query 341 CGAACAAGGACTTCTTCGACCTGTCCGTCATCGACGGGTTCAACGTGCCGATGAGCTTCG 400

|||||||||||||||||||||||||||||||||||||||||||||| || ||||||||||

Sbjct 401 CGAACAAGGACTTCTTCGACCTGTCCGTCATCGACGGGTTCAACGTTCCCATGAGCTTCG 460

Query 401 AGCCCGT 407

|||||||

Sbjct 461 AGCCCGT 467

>EG:HORVU5Hr1G051970.2 cdna chromosome:IBSC_v2:chr5H:406435574:406436438:1 gene:HORVU5Hr1G051970

gene_biotype:protein_coding transcript_biotype:protein_coding

Length=799

Score = 442 bits (223), Expect = 2e-122

Identities = 256/267 (96%), Gaps = 0/267 (0%)

Strand=Plus/Plus

Query 414 GTGCCGTGCTGCGCGGTGCGCCACGGACATCACCAAGGATTGCCTCAAGGAGCTGCAGGT 473

|||||||||||||||||| |||||||||||||||||||| ||||||||||||||||||||

Sbjct 478 GTGCCGTGCTGCGCGGTGTGCCACGGACATCACCAAGGAGTGCCTCAAGGAGCTGCAGGT 537

Query 474 GCCGGGAGGGTGCGCGAGCGCGTGCGGCAAGTTCGGCGGCGACACCTACTGCTGCCGTGG 533

||||||||||||||||||||||||||||||||||||||||||||||||||||||||| ||

Sbjct 538 GCCGGGAGGGTGCGCGAGCGCGTGCGGCAAGTTCGGCGGCGACACCTACTGCTGCCGGGG 597

Query 534 CCAGTTTGAGCACAACTGCCCGCCGACCAACTACTCAATGTTCTTCAAGGGCAAATGCCC 593

|||||| ||||||||||| |||||||||||||| || | ||||||||||||||| |||||

Sbjct 598 CCAGTTCGAGCACAACTGTCCGCCGACCAACTATTCGAAGTTCTTCAAGGGCAAGTGCCC 657

Query 594 CGACGCCTATAGCTATGCGAAGGACGACCAGACCAGCACCTTCACCTGCCCTGCCGGAAC 653

|||||||||||||||||| |||||||||||||||||||||||||||||||||||||||||

Sbjct 658 CGACGCCTATAGCTATGCCAAGGACGACCAGACCAGCACCTTCACCTGCCCTGCCGGAAC 717

Query 654 CAACTACCAGATCGTCCTCTGCCCTTA 680

||||||||||||||||||||||||||

Sbjct 718 AAACTACCAGATCGTCCTCTGCCCTTA 744

Score = 311 bits (157), Expect = 5e-83

Identities = 190/201 (95%), Gaps = 0/201 (0%)

Strand=Plus/Plus

Query 24 CCTCCTGGCCGGTCTACTCCTAGCCGCCCTCGCCGCCACCACAGACGCGGCCACCATCAC 83

||||||||||||||| ||||| |||||||||||||| | |||||||||||||||||||||

Sbjct 80 CCTCCTGGCCGGTCTCCTCCTTGCCGCCCTCGCCGCAAGCACAGACGCGGCCACCATCAC 139

Query 84 CGTCGTCAACCGGTGCTCCTACACGGTGTGGCCGGGCGCGCTCCCGGGTGGCGGTGTGCG 143

||||||||||||||||||||||||||||||||||||||||||||| |||||||| |||||

Sbjct 140 CGTCGTCAACCGGTGCTCCTACACGGTGTGGCCGGGCGCGCTCCCAGGTGGCGGCGTGCG 199

Query 144 TCTCGACCCAGGCCAGTCGTGGGCGCTGAACATGCCCGCGGGTACCGCGGGCGCCAGGGT 203

|||||||||||||||||||||||| ||||||||||| || || |||||||||||||||||

Sbjct 200 TCTCGACCCAGGCCAGTCGTGGGCTCTGAACATGCCGGCCGGCACCGCGGGCGCCAGGGT 259

Query 204 GTGGCCGCGCACCGGGTGCAC 224

|||||||||||| ||||||||

Sbjct 260 GTGGCCGCGCACGGGGTGCAC 280

Score = 278 bits (140), Expect = 7e-73

Identities = 176/188 (94%), Gaps = 0/188 (0%)

Strand=Plus/Plus

Query 220 TGCACCTTCGACGGCAGCGGCCGCGGCCGGTGCATCACCGGCGACTGCAACGGTGTGCTG 279

||||||||||||||||||||||| ||||||||||||||||| |||||| ||| | ||||

Sbjct 281 TGCACCTTCGACGGCAGCGGCCGTGGCCGGTGCATCACCGGAGACTGCGGCGGCGCGCTG 340

Query 280 GCATGCCGGGTGTCCGGCCAGCAGCCGACCACGCTGGCCGAGTACACCCTGGGCCAGGGC 339

|||||||| ||||||||||||||||| |||||| ||||||||||||||||||| ||||||

Sbjct 341 GCATGCCGCGTGTCCGGCCAGCAGCCCACCACGTTGGCCGAGTACACCCTGGGACAGGGC 400

Query 340 GCGAACAAGGACTTCTTCGACCTGTCCGTCATCGACGGGTTCAACGTGCCGATGAGCTTC 399

||||||||||||||||||||||||||||||||||||||||||||||| || |||||||||

Sbjct 401 GCGAACAAGGACTTCTTCGACCTGTCCGTCATCGACGGGTTCAACGTTCCCATGAGCTTC 460

Query 400 GAGCCCGT 407

||||||||

Sbjct 461 GAGCCCGT 468

>EG:HORVU5Hr1G005180.5 cdna chromosome:IBSC_v2:chr5H:8598547:8736897:-1 gene:HORVU5Hr1G005180

gene_biotype:protein_coding transcript_biotype:protein_coding

Length=942

Score = 295 bits (149), Expect = 3e-78

Identities = 200/217 (92%), Gaps = 0/217 (0%)

Strand=Plus/Plus

Query 462 GGAGCTGCAGGTGCCGGGAGGGTGCGCGAGCGCGTGCGGCAAGTTCGGCGGCGACACCTA 521

||||||||||||||| ||||||||||| ||||||||||||||||| ||||||||||||||

Sbjct 496 GGAGCTGCAGGTGCCCGGAGGGTGCGCAAGCGCGTGCGGCAAGTTTGGCGGCGACACCTA 555

Query 522 CTGCTGCCGTGGCCAGTTTGAGCACAACTGCCCGCCGACCAACTACTCAATGTTCTTCAA 581

||||||||| |||||||| ||||||||||||||||| ||| ||||||| | |||||||||

Sbjct 556 CTGCTGCCGGGGCCAGTTCGAGCACAACTGCCCGCCAACCTACTACTCGAAGTTCTTCAA 615

Query 582 GGGCAAATGCCCCGACGCCTATAGCTATGCGAAGGACGACCAGACCAGCACCTTCACCTG 641

|||||| |||||||||||||| ||||| || |||||||||||||||||||||||||| ||

Sbjct 616 GGGCAAGTGCCCCGACGCCTACAGCTACGCCAAGGACGACCAGACCAGCACCTTCACATG 675

Query 642 CCCTGCCGGAACCAACTACCAGATCGTCCTCTGCCCT 678

|| ||||||||||||||||||||||| |||||||||

Sbjct 676 TCCCGCCGGAACCAACTACCAGATCGTGCTCTGCCCT 712

Score = 210 bits (106), Expect = 1e-52

Identities = 172/194 (89%), Gaps = 0/194 (0%)

Strand=Plus/Plus

Query 74 CCACCATCACCGTCGTCAACCGGTGCTCCTACACGGTGTGGCCGGGCGCGCTCCCGGGTG 133

||||||| ||||| || |||||||||||||| ||| | ||||| ||||||||||| || |

Sbjct 102 CCACCATAACCGTGGTGAACCGGTGCTCCTATACGATATGGCCAGGCGCGCTCCCAGGCG 161

Query 134 GCGGTGTGCGTCTCGACCCAGGCCAGTCGTGGGCGCTGAACATGCCCGCGGGTACCGCGG 193

|||| | |||||||||||| |||||||||||| ||| || |||||||| || || ||||

Sbjct 162 GCGGCGCGCGTCTCGACCCGGGCCAGTCGTGGCAGCTCAATATGCCCGCAGGCACGGCGG 221

Query 194 GCGCCAGGGTGTGGCCGCGCACCGGGTGCACCTTCGACGGCAGCGGCCGCGGCCGGTGCA 253

|||||||||||||||||||||| ||||||||||||||| |||||||||| ||||||||||

Sbjct 222 GCGCCAGGGTGTGGCCGCGCACGGGGTGCACCTTCGACCGCAGCGGCCGTGGCCGGTGCA 281

Query 254 TCACCGGCGACTGC 267

||||||||||||||

Sbjct 282 TCACCGGCGACTGC 295

Score = 137 bits (69), Expect = 2e-30

Identities = 111/125 (89%), Gaps = 0/125 (0%)

Strand=Plus/Plus

Query 283 TGCCGGGTGTCCGGCCAGCAGCCGACCACGCTGGCCGAGTACACCCTGGGCCAGGGCGCG 342

||||| ||||||||| ||||||| ||||||||||||||||||| || |||||||||| |

Sbjct 311 TGCCGCGTGTCCGGCGAGCAGCCAGCCACGCTGGCCGAGTACACGCTCGGCCAGGGCGGG 370

Query 343 AACAAGGACTTCTTCGACCTGTCCGTCATCGACGGGTTCAACGTGCCGATGAGCTTCGAG 402

||| |||||| ||||||||||||||||| ||||||||||| ||||| ||||||||| ||

Sbjct 371 AACCGGGACTTTTTCGACCTGTCCGTCATAGACGGGTTCAATGTGCCCATGAGCTTCCAG 430

Query 403 CCCGT 407

|||||

Sbjct 431 CCCGT 435

>EG:HORVU5Hr1G005180.1 cdna chromosome:IBSC_v2:chr5H:8595475:8736940:-1 gene:HORVU5Hr1G005180

gene_biotype:protein_coding transcript_biotype:protein_coding

Length=1249

Score = 295 bits (149), Expect = 3e-78

Identities = 200/217 (92%), Gaps = 0/217 (0%)

Strand=Plus/Plus

Query 462 GGAGCTGCAGGTGCCGGGAGGGTGCGCGAGCGCGTGCGGCAAGTTCGGCGGCGACACCTA 521

||||||||||||||| ||||||||||| ||||||||||||||||| ||||||||||||||

Sbjct 539 GGAGCTGCAGGTGCCCGGAGGGTGCGCAAGCGCGTGCGGCAAGTTTGGCGGCGACACCTA 598

Query 522 CTGCTGCCGTGGCCAGTTTGAGCACAACTGCCCGCCGACCAACTACTCAATGTTCTTCAA 581

||||||||| |||||||| ||||||||||||||||| ||| ||||||| | |||||||||

Sbjct 599 CTGCTGCCGGGGCCAGTTCGAGCACAACTGCCCGCCAACCTACTACTCGAAGTTCTTCAA 658

Query 582 GGGCAAATGCCCCGACGCCTATAGCTATGCGAAGGACGACCAGACCAGCACCTTCACCTG 641

|||||| |||||||||||||| ||||| || |||||||||||||||||||||||||| ||

Sbjct 659 GGGCAAGTGCCCCGACGCCTACAGCTACGCCAAGGACGACCAGACCAGCACCTTCACATG 718

Query 642 CCCTGCCGGAACCAACTACCAGATCGTCCTCTGCCCT 678

|| ||||||||||||||||||||||| |||||||||

Sbjct 719 TCCCGCCGGAACCAACTACCAGATCGTGCTCTGCCCT 755

Score = 210 bits (106), Expect = 1e-52

Identities = 172/194 (89%), Gaps = 0/194 (0%)

Strand=Plus/Plus

Query 74 CCACCATCACCGTCGTCAACCGGTGCTCCTACACGGTGTGGCCGGGCGCGCTCCCGGGTG 133

||||||| ||||| || |||||||||||||| ||| | ||||| ||||||||||| || |

Sbjct 145 CCACCATAACCGTGGTGAACCGGTGCTCCTATACGATATGGCCAGGCGCGCTCCCAGGCG 204

Query 134 GCGGTGTGCGTCTCGACCCAGGCCAGTCGTGGGCGCTGAACATGCCCGCGGGTACCGCGG 193

|||| | |||||||||||| |||||||||||| ||| || |||||||| || || ||||

Sbjct 205 GCGGCGCGCGTCTCGACCCGGGCCAGTCGTGGCAGCTCAATATGCCCGCAGGCACGGCGG 264

Query 194 GCGCCAGGGTGTGGCCGCGCACCGGGTGCACCTTCGACGGCAGCGGCCGCGGCCGGTGCA 253

|||||||||||||||||||||| ||||||||||||||| |||||||||| ||||||||||

Sbjct 265 GCGCCAGGGTGTGGCCGCGCACGGGGTGCACCTTCGACCGCAGCGGCCGTGGCCGGTGCA 324

Query 254 TCACCGGCGACTGC 267

||||||||||||||

Sbjct 325 TCACCGGCGACTGC 338

Score = 137 bits (69), Expect = 2e-30

Identities = 111/125 (89%), Gaps = 0/125 (0%)

Strand=Plus/Plus

Query 283 TGCCGGGTGTCCGGCCAGCAGCCGACCACGCTGGCCGAGTACACCCTGGGCCAGGGCGCG 342

||||| ||||||||| ||||||| ||||||||||||||||||| || |||||||||| |

Sbjct 354 TGCCGCGTGTCCGGCGAGCAGCCAGCCACGCTGGCCGAGTACACGCTCGGCCAGGGCGGG 413

Query 343 AACAAGGACTTCTTCGACCTGTCCGTCATCGACGGGTTCAACGTGCCGATGAGCTTCGAG 402

||| |||||| ||||||||||||||||| ||||||||||| ||||| ||||||||| ||

Sbjct 414 AACCGGGACTTTTTCGACCTGTCCGTCATAGACGGGTTCAATGTGCCCATGAGCTTCCAG 473

Query 403 CCCGT 407

|||||

Sbjct 474 CCCGT 478

>EG:HORVU5Hr1G005180.3 cdna chromosome:IBSC_v2:chr5H:8597589:8736913:-1 gene:HORVU5Hr1G005180

gene_biotype:protein_coding transcript_biotype:protein_coding

Length=1916

Score = 295 bits (149), Expect = 3e-78

Identities = 200/217 (92%), Gaps = 0/217 (0%)

Strand=Plus/Plus

Query 462 GGAGCTGCAGGTGCCGGGAGGGTGCGCGAGCGCGTGCGGCAAGTTCGGCGGCGACACCTA 521

||||||||||||||| ||||||||||| ||||||||||||||||| ||||||||||||||

Sbjct 512 GGAGCTGCAGGTGCCCGGAGGGTGCGCAAGCGCGTGCGGCAAGTTTGGCGGCGACACCTA 571

Query 522 CTGCTGCCGTGGCCAGTTTGAGCACAACTGCCCGCCGACCAACTACTCAATGTTCTTCAA 581

||||||||| |||||||| ||||||||||||||||| ||| ||||||| | |||||||||

Sbjct 572 CTGCTGCCGGGGCCAGTTCGAGCACAACTGCCCGCCAACCTACTACTCGAAGTTCTTCAA 631

Query 582 GGGCAAATGCCCCGACGCCTATAGCTATGCGAAGGACGACCAGACCAGCACCTTCACCTG 641

|||||| |||||||||||||| ||||| || |||||||||||||||||||||||||| ||

Sbjct 632 GGGCAAGTGCCCCGACGCCTACAGCTACGCCAAGGACGACCAGACCAGCACCTTCACATG 691

Query 642 CCCTGCCGGAACCAACTACCAGATCGTCCTCTGCCCT 678

|| ||||||||||||||||||||||| |||||||||

Sbjct 692 TCCCGCCGGAACCAACTACCAGATCGTGCTCTGCCCT 728

Score = 210 bits (106), Expect = 1e-52

Identities = 172/194 (89%), Gaps = 0/194 (0%)

Strand=Plus/Plus

Query 74 CCACCATCACCGTCGTCAACCGGTGCTCCTACACGGTGTGGCCGGGCGCGCTCCCGGGTG 133

||||||| ||||| || |||||||||||||| ||| | ||||| ||||||||||| || |

Sbjct 118 CCACCATAACCGTGGTGAACCGGTGCTCCTATACGATATGGCCAGGCGCGCTCCCAGGCG 177

Query 134 GCGGTGTGCGTCTCGACCCAGGCCAGTCGTGGGCGCTGAACATGCCCGCGGGTACCGCGG 193

|||| | |||||||||||| |||||||||||| ||| || |||||||| || || ||||

Sbjct 178 GCGGCGCGCGTCTCGACCCGGGCCAGTCGTGGCAGCTCAATATGCCCGCAGGCACGGCGG 237

Query 194 GCGCCAGGGTGTGGCCGCGCACCGGGTGCACCTTCGACGGCAGCGGCCGCGGCCGGTGCA 253

|||||||||||||||||||||| ||||||||||||||| |||||||||| ||||||||||

Sbjct 238 GCGCCAGGGTGTGGCCGCGCACGGGGTGCACCTTCGACCGCAGCGGCCGTGGCCGGTGCA 297

Query 254 TCACCGGCGACTGC 267

||||||||||||||

Sbjct 298 TCACCGGCGACTGC 311

Score = 137 bits (69), Expect = 2e-30

Identities = 111/125 (89%), Gaps = 0/125 (0%)

Strand=Plus/Plus

Query 283 TGCCGGGTGTCCGGCCAGCAGCCGACCACGCTGGCCGAGTACACCCTGGGCCAGGGCGCG 342

||||| ||||||||| ||||||| ||||||||||||||||||| || |||||||||| |

Sbjct 327 TGCCGCGTGTCCGGCGAGCAGCCAGCCACGCTGGCCGAGTACACGCTCGGCCAGGGCGGG 386

Query 343 AACAAGGACTTCTTCGACCTGTCCGTCATCGACGGGTTCAACGTGCCGATGAGCTTCGAG 402

||| |||||| ||||||||||||||||| ||||||||||| ||||| ||||||||| ||

Sbjct 387 AACCGGGACTTTTTCGACCTGTCCGTCATAGACGGGTTCAATGTGCCCATGAGCTTCCAG 446

Query 403 CCCGT 407

|||||

Sbjct 447 CCCGT 451

>EG:HORVU5Hr1G005180.4 cdna chromosome:IBSC_v2:chr5H:8598500:8736900:-1 gene:HORVU5Hr1G005180

gene_biotype:protein_coding transcript_biotype:protein_coding

Length=992

Score = 295 bits (149), Expect = 3e-78

Identities = 200/217 (92%), Gaps = 0/217 (0%)

Strand=Plus/Plus

Query 462 GGAGCTGCAGGTGCCGGGAGGGTGCGCGAGCGCGTGCGGCAAGTTCGGCGGCGACACCTA 521

||||||||||||||| ||||||||||| ||||||||||||||||| ||||||||||||||

Sbjct 499 GGAGCTGCAGGTGCCCGGAGGGTGCGCAAGCGCGTGCGGCAAGTTTGGCGGCGACACCTA 558

Query 522 CTGCTGCCGTGGCCAGTTTGAGCACAACTGCCCGCCGACCAACTACTCAATGTTCTTCAA 581

||||||||| |||||||| ||||||||||||||||| ||| ||||||| | |||||||||

Sbjct 559 CTGCTGCCGGGGCCAGTTCGAGCACAACTGCCCGCCAACCTACTACTCGAAGTTCTTCAA 618

Query 582 GGGCAAATGCCCCGACGCCTATAGCTATGCGAAGGACGACCAGACCAGCACCTTCACCTG 641

|||||| |||||||||||||| ||||| || |||||||||||||||||||||||||| ||

Sbjct 619 GGGCAAGTGCCCCGACGCCTACAGCTACGCCAAGGACGACCAGACCAGCACCTTCACATG 678

Query 642 CCCTGCCGGAACCAACTACCAGATCGTCCTCTGCCCT 678

|| ||||||||||||||||||||||| |||||||||

Sbjct 679 TCCCGCCGGAACCAACTACCAGATCGTGCTCTGCCCT 715

Score = 210 bits (106), Expect = 1e-52

Identities = 172/194 (89%), Gaps = 0/194 (0%)

Strand=Plus/Plus

Query 74 CCACCATCACCGTCGTCAACCGGTGCTCCTACACGGTGTGGCCGGGCGCGCTCCCGGGTG 133

||||||| ||||| || |||||||||||||| ||| | ||||| ||||||||||| || |

Sbjct 105 CCACCATAACCGTGGTGAACCGGTGCTCCTATACGATATGGCCAGGCGCGCTCCCAGGCG 164

Query 134 GCGGTGTGCGTCTCGACCCAGGCCAGTCGTGGGCGCTGAACATGCCCGCGGGTACCGCGG 193

|||| | |||||||||||| |||||||||||| ||| || |||||||| || || ||||

Sbjct 165 GCGGCGCGCGTCTCGACCCGGGCCAGTCGTGGCAGCTCAATATGCCCGCAGGCACGGCGG 224

Query 194 GCGCCAGGGTGTGGCCGCGCACCGGGTGCACCTTCGACGGCAGCGGCCGCGGCCGGTGCA 253

|||||||||||||||||||||| ||||||||||||||| |||||||||| ||||||||||

Sbjct 225 GCGCCAGGGTGTGGCCGCGCACGGGGTGCACCTTCGACCGCAGCGGCCGTGGCCGGTGCA 284

Query 254 TCACCGGCGACTGC 267

||||||||||||||

Sbjct 285 TCACCGGCGACTGC 298

Score = 137 bits (69), Expect = 2e-30

Identities = 111/125 (89%), Gaps = 0/125 (0%)

Strand=Plus/Plus

Query 283 TGCCGGGTGTCCGGCCAGCAGCCGACCACGCTGGCCGAGTACACCCTGGGCCAGGGCGCG 342

||||| ||||||||| ||||||| ||||||||||||||||||| || |||||||||| |

Sbjct 314 TGCCGCGTGTCCGGCGAGCAGCCAGCCACGCTGGCCGAGTACACGCTCGGCCAGGGCGGG 373

Query 343 AACAAGGACTTCTTCGACCTGTCCGTCATCGACGGGTTCAACGTGCCGATGAGCTTCGAG 402

||| |||||| ||||||||||||||||| ||||||||||| ||||| ||||||||| ||

Sbjct 374 AACCGGGACTTTTTCGACCTGTCCGTCATAGACGGGTTCAATGTGCCCATGAGCTTCCAG 433

Query 403 CCCGT 407

|||||

Sbjct 434 CCCGT 438

>EG:HORVU5Hr1G005180.2 cdna chromosome:IBSC_v2:chr5H:8597589:8599517:-1 gene:HORVU5Hr1G005180

gene_biotype:protein_coding transcript_biotype:protein_coding

Length=1929

Score = 295 bits (149), Expect = 3e-78

Identities = 200/217 (92%), Gaps = 0/217 (0%)

Strand=Plus/Plus

Query 462 GGAGCTGCAGGTGCCGGGAGGGTGCGCGAGCGCGTGCGGCAAGTTCGGCGGCGACACCTA 521

||||||||||||||| ||||||||||| ||||||||||||||||| ||||||||||||||

Sbjct 525 GGAGCTGCAGGTGCCCGGAGGGTGCGCAAGCGCGTGCGGCAAGTTTGGCGGCGACACCTA 584

Query 522 CTGCTGCCGTGGCCAGTTTGAGCACAACTGCCCGCCGACCAACTACTCAATGTTCTTCAA 581

||||||||| |||||||| ||||||||||||||||| ||| ||||||| | |||||||||

Sbjct 585 CTGCTGCCGGGGCCAGTTCGAGCACAACTGCCCGCCAACCTACTACTCGAAGTTCTTCAA 644

Query 582 GGGCAAATGCCCCGACGCCTATAGCTATGCGAAGGACGACCAGACCAGCACCTTCACCTG 641

|||||| |||||||||||||| ||||| || |||||||||||||||||||||||||| ||

Sbjct 645 GGGCAAGTGCCCCGACGCCTACAGCTACGCCAAGGACGACCAGACCAGCACCTTCACATG 704

Query 642 CCCTGCCGGAACCAACTACCAGATCGTCCTCTGCCCT 678

|| ||||||||||||||||||||||| |||||||||

Sbjct 705 TCCCGCCGGAACCAACTACCAGATCGTGCTCTGCCCT 741

Score = 226 bits (114), Expect = 2e-57

Identities = 174/194 (90%), Gaps = 0/194 (0%)

Strand=Plus/Plus

Query 74 CCACCATCACCGTCGTCAACCGGTGCTCCTACACGGTGTGGCCGGGCGCGCTCCCGGGTG 133

||||||| ||||| || |||||||||||||| ||| | ||||| ||||||||||| || |

Sbjct 131 CCACCATAACCGTGGTGAACCGGTGCTCCTATACGATATGGCCAGGCGCGCTCCCAGGCG 190

Query 134 GCGGTGTGCGTCTCGACCCAGGCCAGTCGTGGGCGCTGAACATGCCCGCGGGTACCGCGG 193

|||| | |||||||||||| |||||||||||| ||| |||||||||||||| || ||||

Sbjct 191 GCGGCGCGCGTCTCGACCCGGGCCAGTCGTGGCAGCTCAACATGCCCGCGGGCACGGCGG 250

Query 194 GCGCCAGGGTGTGGCCGCGCACCGGGTGCACCTTCGACGGCAGCGGCCGCGGCCGGTGCA 253

|||||||||||||||||||||| ||||||||||||||| |||||||||| ||||||||||

Sbjct 251 GCGCCAGGGTGTGGCCGCGCACGGGGTGCACCTTCGACCGCAGCGGCCGTGGCCGGTGCA 310

Query 254 TCACCGGCGACTGC 267

||||||||||||||

Sbjct 311 TCACCGGCGACTGC 324

Score = 137 bits (69), Expect = 2e-30

Identities = 111/125 (89%), Gaps = 0/125 (0%)

Strand=Plus/Plus

Query 283 TGCCGGGTGTCCGGCCAGCAGCCGACCACGCTGGCCGAGTACACCCTGGGCCAGGGCGCG 342

||||| ||||||||| ||||||| ||||||||||||||||||| || |||||||||| |

Sbjct 340 TGCCGCGTGTCCGGCGAGCAGCCAGCCACGCTGGCCGAGTACACGCTCGGCCAGGGCGGG 399

Query 343 AACAAGGACTTCTTCGACCTGTCCGTCATCGACGGGTTCAACGTGCCGATGAGCTTCGAG 402

||| |||||| ||||||||||||||||| ||||||||||| ||||| ||||||||| ||

Sbjct 400 AACCGGGACTTTTTCGACCTGTCCGTCATAGACGGGTTCAATGTGCCCATGAGCTTCCAG 459

Query 403 CCCGT 407

|||||

Sbjct 460 CCCGT 464

>EG:HORVU5Hr1G005310.3 cdna chromosome:IBSC_v2:chr5H:8830987:8832925:1 gene:HORVU5Hr1G005310

gene_biotype:protein_coding transcript_biotype:protein_coding

Length=864

Score = 287 bits (145), Expect = 7e-76

Identities = 187/201 (93%), Gaps = 0/201 (0%)

Strand=Plus/Plus

Query 478 GGAGGGTGCGCGAGCGCGTGCGGCAAGTTCGGCGGCGACACCTACTGCTGCCGTGGCCAG 537

||||||||||||||||||||||||||||| |||||||||||||||||||| |||||||||

Sbjct 4 GGAGGGTGCGCGAGCGCGTGCGGCAAGTTTGGCGGCGACACCTACTGCTGTCGTGGCCAG 63

Query 538 TTTGAGCACAACTGCCCGCCGACCAACTACTCAATGTTCTTCAAGGGCAAATGCCCCGAC 597

|| |||||||||||||||||||| ||||||| | ||||||||||||||| |||||||||

Sbjct 64 TTCGAGCACAACTGCCCGCCGACATACTACTCGAGGTTCTTCAAGGGCAAGTGCCCCGAC 123

Query 598 GCCTATAGCTATGCGAAGGACGACCAGACCAGCACCTTCACCTGCCCTGCCGGAACCAAC 657

||||| ||||| || ||||| |||||||||||||||||||||||||| ||||||||||||

Sbjct 124 GCCTACAGCTACGCCAAGGATGACCAGACCAGCACCTTCACCTGCCCGGCCGGAACCAAC 183

Query 658 TACCAGATCGTCCTCTGCCCT 678

||||||||||| |||||||||

Sbjct 184 TACCAGATCGTGCTCTGCCCT 204

>EG:HORVU5Hr1G005310.1 cdna chromosome:IBSC_v2:chr5H:8830987:8832796:1 gene:HORVU5Hr1G005310

gene_biotype:protein_coding transcript_biotype:protein_coding

Length=630

Score = 287 bits (145), Expect = 7e-76

Identities = 187/201 (93%), Gaps = 0/201 (0%)

Strand=Plus/Plus

Query 478 GGAGGGTGCGCGAGCGCGTGCGGCAAGTTCGGCGGCGACACCTACTGCTGCCGTGGCCAG 537

||||||||||||||||||||||||||||| |||||||||||||||||||| |||||||||

Sbjct 4 GGAGGGTGCGCGAGCGCGTGCGGCAAGTTTGGCGGCGACACCTACTGCTGTCGTGGCCAG 63

Query 538 TTTGAGCACAACTGCCCGCCGACCAACTACTCAATGTTCTTCAAGGGCAAATGCCCCGAC 597

|| |||||||||||||||||||| ||||||| | ||||||||||||||| |||||||||

Sbjct 64 TTCGAGCACAACTGCCCGCCGACATACTACTCGAGGTTCTTCAAGGGCAAGTGCCCCGAC 123

Query 598 GCCTATAGCTATGCGAAGGACGACCAGACCAGCACCTTCACCTGCCCTGCCGGAACCAAC 657

||||| ||||| || ||||| |||||||||||||||||||||||||| ||||||||||||

Sbjct 124 GCCTACAGCTACGCCAAGGATGACCAGACCAGCACCTTCACCTGCCCGGCCGGAACCAAC 183

Query 658 TACCAGATCGTCCTCTGCCCT 678

||||||||||| |||||||||

Sbjct 184 TACCAGATCGTGCTCTGCCCT 204

>EG:HORVU5Hr1G005310.2 cdna chromosome:IBSC_v2:chr5H:8830987:8832896:1 gene:HORVU5Hr1G005310

gene_biotype:protein_coding transcript_biotype:protein_coding

Length=1910

Score = 287 bits (145), Expect = 7e-76

Identities = 187/201 (93%), Gaps = 0/201 (0%)

Strand=Plus/Plus

Query 478 GGAGGGTGCGCGAGCGCGTGCGGCAAGTTCGGCGGCGACACCTACTGCTGCCGTGGCCAG 537

||||||||||||||||||||||||||||| |||||||||||||||||||| |||||||||

Sbjct 4 GGAGGGTGCGCGAGCGCGTGCGGCAAGTTTGGCGGCGACACCTACTGCTGTCGTGGCCAG 63

Query 538 TTTGAGCACAACTGCCCGCCGACCAACTACTCAATGTTCTTCAAGGGCAAATGCCCCGAC 597

|| |||||||||||||||||||| ||||||| | ||||||||||||||| |||||||||

Sbjct 64 TTCGAGCACAACTGCCCGCCGACATACTACTCGAGGTTCTTCAAGGGCAAGTGCCCCGAC 123

Query 598 GCCTATAGCTATGCGAAGGACGACCAGACCAGCACCTTCACCTGCCCTGCCGGAACCAAC 657

||||| ||||| || ||||| |||||||||||||||||||||||||| ||||||||||||

Sbjct 124 GCCTACAGCTACGCCAAGGATGACCAGACCAGCACCTTCACCTGCCCGGCCGGAACCAAC 183

Query 658 TACCAGATCGTCCTCTGCCCT 678

||||||||||| |||||||||

Sbjct 184 TACCAGATCGTGCTCTGCCCT 204

>EG:HORVU5Hr1G005180.8 cdna chromosome:IBSC_v2:chr5H:8736390:8737025:-1 gene:HORVU5Hr1G005180

gene_biotype:protein_coding transcript_biotype:protein_coding

Length=636

Score = 210 bits (106), Expect = 1e-52

Identities = 172/194 (89%), Gaps = 0/194 (0%)

Strand=Plus/Plus

Query 74 CCACCATCACCGTCGTCAACCGGTGCTCCTACACGGTGTGGCCGGGCGCGCTCCCGGGTG 133

||||||| ||||| || |||||||||||||| ||| | ||||| ||||||||||| || |

Sbjct 230 CCACCATAACCGTGGTGAACCGGTGCTCCTATACGATATGGCCAGGCGCGCTCCCAGGCG 289

Query 134 GCGGTGTGCGTCTCGACCCAGGCCAGTCGTGGGCGCTGAACATGCCCGCGGGTACCGCGG 193

|||| | |||||||||||| |||||||||||| ||| || |||||||| || || ||||

Sbjct 290 GCGGCGCGCGTCTCGACCCGGGCCAGTCGTGGCAGCTCAATATGCCCGCAGGCACGGCGG 349

Query 194 GCGCCAGGGTGTGGCCGCGCACCGGGTGCACCTTCGACGGCAGCGGCCGCGGCCGGTGCA 253

|||||||||||||||||||||| ||||||||||||||| |||||||||| ||||||||||

Sbjct 350 GCGCCAGGGTGTGGCCGCGCACGGGGTGCACCTTCGACCGCAGCGGCCGTGGCCGGTGCA 409

Query 254 TCACCGGCGACTGC 267

||||||||||||||

Sbjct 410 TCACCGGCGACTGC 423

Score = 137 bits (69), Expect = 2e-30

Identities = 111/125 (89%), Gaps = 0/125 (0%)

Strand=Plus/Plus

Query 283 TGCCGGGTGTCCGGCCAGCAGCCGACCACGCTGGCCGAGTACACCCTGGGCCAGGGCGCG 342

||||| ||||||||| ||||||| ||||||||||||||||||| || |||||||||| |

Sbjct 439 TGCCGCGTGTCCGGCGAGCAGCCAGCCACGCTGGCCGAGTACACGCTCGGCCAGGGCGGG 498

Query 343 AACAAGGACTTCTTCGACCTGTCCGTCATCGACGGGTTCAACGTGCCGATGAGCTTCGAG 402

||| |||||| ||||||||||||||||| ||||||||||| ||||| ||||||||| ||

Sbjct 499 AACCGGGACTTTTTCGACCTGTCCGTCATAGACGGGTTCAATGTGCCCATGAGCTTCCAG 558

Query 403 CCCGT 407

|||||

Sbjct 559 CCCGT 563

>EG:HORVU5Hr1G005180.7 cdna chromosome:IBSC_v2:chr5H:8614572:8736944:-1 gene:HORVU5Hr1G005180

gene_biotype:protein_coding transcript_biotype:protein_coding

Length=604

Score = 210 bits (106), Expect = 1e-52

Identities = 172/194 (89%), Gaps = 0/194 (0%)

Strand=Plus/Plus

Query 74 CCACCATCACCGTCGTCAACCGGTGCTCCTACACGGTGTGGCCGGGCGCGCTCCCGGGTG 133

||||||| ||||| || |||||||||||||| ||| | ||||| ||||||||||| || |

Sbjct 149 CCACCATAACCGTGGTGAACCGGTGCTCCTATACGATATGGCCAGGCGCGCTCCCAGGCG 208

Query 134 GCGGTGTGCGTCTCGACCCAGGCCAGTCGTGGGCGCTGAACATGCCCGCGGGTACCGCGG 193

|||| | |||||||||||| |||||||||||| ||| || |||||||| || || ||||

Sbjct 209 GCGGCGCGCGTCTCGACCCGGGCCAGTCGTGGCAGCTCAATATGCCCGCAGGCACGGCGG 268

Query 194 GCGCCAGGGTGTGGCCGCGCACCGGGTGCACCTTCGACGGCAGCGGCCGCGGCCGGTGCA 253

|||||||||||||||||||||| ||||||||||||||| |||||||||| ||||||||||

Sbjct 269 GCGCCAGGGTGTGGCCGCGCACGGGGTGCACCTTCGACCGCAGCGGCCGTGGCCGGTGCA 328

Query 254 TCACCGGCGACTGC 267

||||||||||||||

Sbjct 329 TCACCGGCGACTGC 342

Score = 137 bits (69), Expect = 2e-30

Identities = 111/125 (89%), Gaps = 0/125 (0%)

Strand=Plus/Plus

Query 283 TGCCGGGTGTCCGGCCAGCAGCCGACCACGCTGGCCGAGTACACCCTGGGCCAGGGCGCG 342

||||| ||||||||| ||||||| ||||||||||||||||||| || |||||||||| |

Sbjct 358 TGCCGCGTGTCCGGCGAGCAGCCAGCCACGCTGGCCGAGTACACGCTCGGCCAGGGCGGG 417

Query 343 AACAAGGACTTCTTCGACCTGTCCGTCATCGACGGGTTCAACGTGCCGATGAGCTTCGAG 402

||| |||||| ||||||||||||||||| ||||||||||| ||||| ||||||||| ||

Sbjct 418 AACCGGGACTTTTTCGACCTGTCCGTCATAGACGGGTTCAATGTGCCCATGAGCTTCCAG 477

Query 403 CCCGT 407

|||||

Sbjct 478 CCCGT 482

Score = 123 bits (62), Expect = 2e-26

Identities = 62/62 (100%), Gaps = 0/62 (0%)

Strand=Plus/Plus

Query 462 GGAGCTGCAGGTGCCGGGAGGGTGCGCGAGCGCGTGCGGCAAGTTCGGCGGCGACACCTA 521

||||||||||||||||||||||||||||||||||||||||||||||||||||||||||||

Sbjct 543 GGAGCTGCAGGTGCCGGGAGGGTGCGCGAGCGCGTGCGGCAAGTTCGGCGGCGACACCTA 602

Query 522 CT 523

||

Sbjct 603 CT 604

>EG:HORVU5Hr1G051960.1 cdna chromosome:IBSC_v2:chr5H:406424994:406425200:1 gene:HORVU5Hr1G051960

gene_biotype:protein_coding transcript_biotype:protein_coding

Length=207

Score = 190 bits (96), Expect = 1e-46

Identities = 123/132 (93%), Gaps = 0/132 (0%)

Strand=Plus/Plus

Query 462 GGAGCTGCAGGTGCCGGGAGGGTGCGCGAGCGCGTGCGGCAAGTTCGGCGGCGACACCTA 521

||||||||||||||| ||||||||||||||||||||||||||||| ||||||||||||||

Sbjct 65 GGAGCTGCAGGTGCCCGGAGGGTGCGCGAGCGCGTGCGGCAAGTTTGGCGGCGACACCTA 124

Query 522 CTGCTGCCGTGGCCAGTTTGAGCACAACTGCCCGCCGACCAACTACTCAATGTTCTTCAA 581

|||||| ||||||||||| |||||||||||||||||||| ||||||| | |||||||||

Sbjct 125 CTGCTGTCGTGGCCAGTTCGAGCACAACTGCCCGCCGACATACTACTCGAGGTTCTTCAA 184

Query 582 GGGCAAATGCCC 593

|||||| |||||

Sbjct 185 GGGCAAGTGCCC 196

>EG:HORVU5Hr1G051950.1 cdna chromosome:IBSC_v2:chr5H:406424295:406424869:1 gene:HORVU5Hr1G051950

gene_biotype:protein_coding transcript_biotype:protein_coding

Length=575

Score = 159 bits (80), Expect = 4e-37

Identities = 152/176 (86%), Gaps = 0/176 (0%)

Strand=Plus/Plus

Query 74 CCACCATCACCGTCGTCAACCGGTGCTCCTACACGGTGTGGCCGGGCGCGCTCCCGGGTG 133

||||||| ||||| || |||||||||||||| ||| | ||||| ||||||||||| || |

Sbjct 161 CCACCATAACCGTGGTGAACCGGTGCTCCTATACGATATGGCCAGGCGCGCTCCCAGGCG 220

Query 134 GCGGTGTGCGTCTCGACCCAGGCCAGTCGTGGGCGCTGAACATGCCCGCGGGTACCGCGG 193

|||| | |||||||||||| |||||||||||| ||| || |||||||| || || ||||

Sbjct 221 GCGGCGCGCGTCTCGACCCGGGCCAGTCGTGGCAGCTCAATATGCCCGCAGGCACGGCGG 280

Query 194 GCGCCAGGGTGTGGCCGCGCACCGGGTGCACCTTCGACGGCAGCGGCCGCGGCCGG 249

|||||||||||||||||||||| ||| ||||||||||| ||||||| || ||||||

Sbjct 281 GCGCCAGGGTGTGGCCGCGCACGGGGCGCACCTTCGACCGCAGCGGACGTGGCCGG 336

Score = 137 bits (69), Expect = 2e-30

Identities = 87/93 (94%), Gaps = 0/93 (0%)

Strand=Plus/Plus

Query 175 ATGCCCGCGGGTACCGCGGGCGCCAGGGTGTGGCCGCGCACCGGGTGCACCTTCGACGGC 234

|||||||| || || |||||||||||||||||||||||||| ||||||||||||||| ||

Sbjct 347 ATGCCCGCAGGCACGGCGGGCGCCAGGGTGTGGCCGCGCACGGGGTGCACCTTCGACCGC 406

Query 235 AGCGGCCGCGGCCGGTGCATCACCGGCGACTGC 267

|||||||| ||||||||||||||||||||||||

Sbjct 407 AGCGGCCGTGGCCGGTGCATCACCGGCGACTGC 439

Score = 129 bits (65), Expect = 4e-28

Identities = 104/117 (89%), Gaps = 0/117 (0%)

Strand=Plus/Plus

Query 283 TGCCGGGTGTCCGGCCAGCAGCCGACCACGCTGGCCGAGTACACCCTGGGCCAGGGCGCG 342

||||| ||||||||| ||||||| ||||||||||||||||||| || |||||||||| |

Sbjct 455 TGCCGCGTGTCCGGCGAGCAGCCAGCCACGCTGGCCGAGTACACGCTCGGCCAGGGCGGG 514

Query 343 AACAAGGACTTCTTCGACCTGTCCGTCATCGACGGGTTCAACGTGCCGATGAGCTTC 399

||| |||||| ||||||||||||||||| ||||||||||| ||||| |||||||||

Sbjct 515 AACCGGGACTTTTTCGACCTGTCCGTCATAGACGGGTTCAATGTGCCCATGAGCTTC 571

>EG:HORVU5Hr1G005310.4 cdna chromosome:IBSC_v2:chr5H:8831045:8831363:1 gene:HORVU5Hr1G005310

gene_biotype:protein_coding transcript_biotype:protein_coding

Length=276

Score = 109 bits (55), Expect = 4e-22

Identities = 70/75 (93%), Gaps = 0/75 (0%)

Strand=Plus/Plus

Query 604 AGCTATGCGAAGGACGACCAGACCAGCACCTTCACCTGCCCTGCCGGAACCAACTACCAG 663

||||| || ||||| |||||||||||||||||||||||||| ||||||||||||||||||

Sbjct 29 AGCTACGCCAAGGATGACCAGACCAGCACCTTCACCTGCCCGGCCGGAACCAACTACCAG 88

Query 664 ATCGTCCTCTGCCCT 678

||||| |||||||||

Sbjct 89 ATCGTGCTCTGCCCT 103

Score = 48.1 bits (24), Expect = 0.001

Identities = 27/28 (96%), Gaps = 0/28 (0%)

Strand=Plus/Plus

Query 533 GCCAGTTTGAGCACAACTGCCCGCCGAC 560

||||||| ||||||||||||||||||||

Sbjct 1 GCCAGTTCGAGCACAACTGCCCGCCGAC 28

>EG:HORVU5Hr1G005510.1 cdna chromosome:IBSC_v2:chr5H:8927171:8927409:1 gene:HORVU5Hr1G005510

gene_biotype:protein_coding transcript_biotype:protein_coding

Length=239

Score = 69.9 bits (35), Expect = 3e-10

Identities = 68/79 (86%), Gaps = 0/79 (0%)

Strand=Plus/Plus

Query 74 CCACCATCACCGTCGTCAACCGGTGCTCCTACACGGTGTGGCCGGGCGCGCTCCCGGGTG 133

||||||| ||||| || |||||||||||||| ||| | ||||| ||||||||||| || |

Sbjct 149 CCACCATAACCGTGGTGAACCGGTGCTCCTATACGATATGGCCAGGCGCGCTCCCAGGCG 208

Query 134 GCGGTGTGCGTCTCGACCC 152

|||| | ||||||||||||

Sbjct 209 GCGGCGCGCGTCTCGACCC 227

## Section 6

## BLAST search of PTO PR5.1 against *Hordeum vulgare* database

Query= PR5.1 TTGAAGAACATTGAGTAGT;

Length=19

Score E

Sequences producing significant alignments: (Bits) Value

EG:HORVU5Hr1G005180.6 cdna chromosome:IBSC_v2:chr5H:8614116:8615... 38.2 0.006

EG:HORVU3Hr1G002670.18 cdna chromosome:IBSC_v2:chr3H:6816099:682... 34.2 0.097

EG:HORVU3Hr1G002670.6 cdna chromosome:IBSC_v2:chr3H:6816093:6821... 34.2 0.097

EG:HORVU3Hr1G002670.37 cdna chromosome:IBSC_v2:chr3H:6816538:682... 34.2 0.097

EG:HORVU3Hr1G002670.23 cdna chromosome:IBSC_v2:chr3H:6816127:682... 34.2 0.097

EG:HORVU3Hr1G002670.1 cdna chromosome:IBSC_v2:chr3H:6816020:6820... 34.2 0.097

EG:HORVU3Hr1G002670.8 cdna chromosome:IBSC_v2:chr3H:6816093:6822... 34.2 0.097

EG:HORVU3Hr1G002670.26 cdna chromosome:IBSC_v2:chr3H:6816155:682... 34.2 0.097

EG:HORVU3Hr1G002670.31 cdna chromosome:IBSC_v2:chr3H:6816182:682... 34.2 0.097

EG:HORVU3Hr1G002670.3 cdna chromosome:IBSC_v2:chr3H:6816020:6821... 34.2 0.097

EG:HORVU3Hr1G002670.19 cdna chromosome:IBSC_v2:chr3H:6816099:682... 34.2 0.097

EG:HORVU3Hr1G002670.25 cdna chromosome:IBSC_v2:chr3H:6816155:682... 34.2 0.097

EG:HORVU3Hr1G002670.14 cdna chromosome:IBSC_v2:chr3H:6816097:682... 34.2 0.097

EG:HORVU3Hr1G002670.36 cdna chromosome:IBSC_v2:chr3H:6816246:682... 34.2 0.097

EG:HORVU3Hr1G002670.33 cdna chromosome:IBSC_v2:chr3H:6816182:682... 34.2 0.097

EG:HORVU3Hr1G002670.27 cdna chromosome:IBSC_v2:chr3H:6816155:682... 34.2 0.097

EG:HORVU3Hr1G002670.5 cdna chromosome:IBSC_v2:chr3H:6816093:6821... 34.2 0.097

EG:HORVU3Hr1G002670.16 cdna chromosome:IBSC_v2:chr3H:6816099:682... 34.2 0.097

EG:HORVU3Hr1G002670.38 cdna chromosome:IBSC_v2:chr3H:6818133:682... 34.2 0.097

EG:HORVU3Hr1G002670.7 cdna chromosome:IBSC_v2:chr3H:6816093:6821... 34.2 0.097

EG:HORVU3Hr1G002670.17 cdna chromosome:IBSC_v2:chr3H:6816099:682... 34.2 0.097

EG:HORVU3Hr1G002670.10 cdna chromosome:IBSC_v2:chr3H:6816094:682... 34.2 0.097

EG:HORVU3Hr1G002670.32 cdna chromosome:IBSC_v2:chr3H:6816182:682... 34.2 0.097

EG:HORVU3Hr1G002670.29 cdna chromosome:IBSC_v2:chr3H:6816171:682... 34.2 0.097

EG:HORVU3Hr1G002670.15 cdna chromosome:IBSC_v2:chr3H:6816099:682... 34.2 0.097

EG:HORVU3Hr1G002670.9 cdna chromosome:IBSC_v2:chr3H:6816093:6822... 34.2 0.097

EG:HORVU3Hr1G002670.12 cdna chromosome:IBSC_v2:chr3H:6816094:682... 34.2 0.097

EG:HORVU3Hr1G002670.39 cdna chromosome:IBSC_v2:chr3H:6818532:682... 34.2 0.097

EG:HORVU3Hr1G002670.20 cdna chromosome:IBSC_v2:chr3H:6816099:682... 34.2 0.097

EG:HORVU3Hr1G002670.30 cdna chromosome:IBSC_v2:chr3H:6816171:682... 34.2 0.097

EG:HORVU3Hr1G002670.35 cdna chromosome:IBSC_v2:chr3H:6816246:682... 34.2 0.097

EG:HORVU3Hr1G002670.28 cdna chromosome:IBSC_v2:chr3H:6816155:682... 34.2 0.097

EG:HORVU3Hr1G002670.11 cdna chromosome:IBSC_v2:chr3H:6816094:682... 34.2 0.097

EG:HORVU3Hr1G002670.2 cdna chromosome:IBSC_v2:chr3H:6816020:6820... 34.2 0.097

EG:HORVU3Hr1G002670.13 cdna chromosome:IBSC_v2:chr3H:6816097:682... 34.2 0.097

EG:HORVU3Hr1G002670.4 cdna chromosome:IBSC_v2:chr3H:6816093:6821... 34.2 0.097

EG:HORVU3Hr1G002670.22 cdna chromosome:IBSC_v2:chr3H:6816127:682... 34.2 0.097

EG:HORVU5Hr1G068970.2 cdna chromosome:IBSC_v2:chr5H:519892674:51... 32.2 0.38

EG:HORVU5Hr1G068970.1 cdna chromosome:IBSC_v2:chr5H:519892631:51... 32.2 0.38

EG:HORVU5Hr1G068970.3 cdna chromosome:IBSC_v2:chr5H:519892774:51... 32.2 0.38

EG:HORVU5Hr1G010300.7 cdna chromosome:IBSC_v2:chr5H:25650197:256... 30.2 1.5

EG:HORVU5Hr1G010300.11 cdna chromosome:IBSC_v2:chr5H:25650694:25... 30.2 1.5

EG:HORVU5Hr1G010300.5 cdna chromosome:IBSC_v2:chr5H:25650194:256... 30.2 1.5

EG:HORVU5Hr1G010300.13 cdna chromosome:IBSC_v2:chr5H:25651735:25... 30.2 1.5

EG:HORVU5Hr1G010300.3 cdna chromosome:IBSC_v2:chr5H:25650184:256... 30.2 1.5

EG:HORVU5Hr1G010300.9 cdna chromosome:IBSC_v2:chr5H:25650503:256... 30.2 1.5

EG:HORVU5Hr1G010300.2 cdna chromosome:IBSC_v2:chr5H:25650078:256... 30.2 1.5

EG:HORVU5Hr1G010300.12 cdna chromosome:IBSC_v2:chr5H:25650697:25... 30.2 1.5

EG:HORVU5Hr1G010300.6 cdna chromosome:IBSC_v2:chr5H:25650194:256... 30.2 1.5

EG:HORVU5Hr1G010300.10 cdna chromosome:IBSC_v2:chr5H:25650640:25... 30.2 1.5

>EG:HORVU5Hr1G005180.6 cdna chromosome:IBSC_v2:chr5H:8614116:8615298:-1 gene:HORVU5Hr1G005180

gene_biotype:protein_coding transcript_biotype:protein_coding

Length=1183

Score = 38.2 bits (19), Expect = 0.006

Identities = 19/19 (100%), Gaps = 0/19 (0%)

Strand=Plus/Minus

Query 1 TTGAAGAACATTGAGTAGT 19

|||||||||||||||||||

Sbjct 785 TTGAAGAACATTGAGTAGT 767

>EG:HORVU3Hr1G002670.18 cdna chromosome:IBSC_v2:chr3H:6816099:6822191:-1 gene:HORVU3Hr1G002670

gene_biotype:protein_coding transcript_biotype:protein_coding

Length=4277

Score = 34.2 bits (17), Expect = 0.097

Identities = 17/17 (100%), Gaps = 0/17 (0%)

Strand=Plus/Minus

Query 3 GAAGAACATTGAGTAGT 19

|||||||||||||||||

Sbjct 768 GAAGAACATTGAGTAGT 752

>EG:HORVU3Hr1G002670.6 cdna chromosome:IBSC_v2:chr3H:6816093:6821209:-1 gene:HORVU3Hr1G002670

gene_biotype:protein_coding transcript_biotype:protein_coding

Length=4151

Score = 34.2 bits (17), Expect = 0.097

Identities = 17/17 (100%), Gaps = 0/17 (0%)

Strand=Plus/Minus

Query 3 GAAGAACATTGAGTAGT 19

|||||||||||||||||

Sbjct 634 GAAGAACATTGAGTAGT 618

>EG:HORVU5Hr1G068970.3 cdna chromosome:IBSC_v2:chr5H:519892774:519897263:-1 gene:HORVU5Hr1G068970

gene_biotype:protein_coding transcript_biotype:protein_coding

Length=4391

Score = 32.2 bits (16), Expect = 0.38

Identities = 16/16 (100%), Gaps = 0/16 (0%)

Strand=Plus/Minus

Query 4 AAGAACATTGAGTAGT 19

||||||||||||||||

Sbjct 4371 AAGAACATTGAGTAGT 4356

>EG:HORVU5Hr1G010300.7 cdna chromosome:IBSC_v2:chr5H:25650197:25653270:1 gene:HORVU5Hr1G010300

gene_biotype:protein_coding transcript_biotype:protein_coding

Length=1353

Score = 30.2 bits (15), Expect = 1.5

Identities = 15/15 (100%), Gaps = 0/15 (0%)

Strand=Plus/Plus

Query 1 TTGAAGAACATTGAG 15

|||||||||||||||

Sbjct 1329 TTGAAGAACATTGAG 1343

## Section 7

## BLAST search of PTO PR5.2 against *Hordeum vulgare* database

Query= PR5.2 oligo AAGAAGTCCTTGTTCGCGC;

Length=19

Score E

Sequences producing significant alignments: (Bits) Value

EG:HORVU5Hr1G005180.6 cdna chromosome:IBSC_v2:chr5H:8614116:8615... 38.2 0.006

EG:HORVU5Hr1G051970.1 cdna chromosome:IBSC_v2:chr5H:406435550:40... 38.2 0.006

EG:HORVU5Hr1G051970.4 cdna chromosome:IBSC_v2:chr5H:406435574:40... 38.2 0.006

EG:HORVU5Hr1G051970.5 cdna chromosome:IBSC_v2:chr5H:406435615:40... 38.2 0.006

EG:HORVU5Hr1G051970.3 cdna chromosome:IBSC_v2:chr5H:406435574:40... 38.2 0.006

EG:HORVU5Hr1G051970.7 cdna chromosome:IBSC_v2:chr5H:406435630:40... 38.2 0.006

EG:HORVU5Hr1G051970.2 cdna chromosome:IBSC_v2:chr5H:406435574:40... 38.2 0.006

EG:HORVU5Hr1G051970.6 cdna chromosome:IBSC_v2:chr5H:406435630:40... 38.2 0.006

EG:HORVU6Hr1G076320.11 cdna chromosome:IBSC_v2:chr6H:525340759:5... 30.2 1.5

EG:HORVU6Hr1G076320.13 cdna chromosome:IBSC_v2:chr6H:525340771:5... 30.2 1.5

EG:HORVU6Hr1G076320.12 cdna chromosome:IBSC_v2:chr6H:525340762:5... 30.2 1.5

EG:HORVU1Hr1G041150.24 cdna chromosome:IBSC_v2:chr1H:292351491:2... 28.2 6.0

EG:HORVU7Hr1G002370.2 cdna chromosome:IBSC_v2:chr7H:4498868:4499... 28.2 6.0

EG:HORVU7Hr1G002370.1 cdna chromosome:IBSC_v2:chr7H:4498848:4499... 28.2 6.0

EG:HORVU5Hr1G012660.29 cdna chromosome:IBSC_v2:chr5H:37812103:37... 28.2 6.0

EG:HORVU5Hr1G012660.6 cdna chromosome:IBSC_v2:chr5H:37812068:378... 28.2 6.0

EG:HORVU5Hr1G012660.69 cdna chromosome:IBSC_v2:chr5H:37812205:37... 28.2 6.0

EG:HORVU5Hr1G012660.34 cdna chromosome:IBSC_v2:chr5H:37812106:37... 28.2 6.0

EG:HORVU5Hr1G012660.17 cdna chromosome:IBSC_v2:chr5H:37812099:37... 28.2 6.0

EG:HORVU3Hr1G054100.1 cdna chromosome:IBSC_v2:chr3H:399272359:39... 26.3 24

EG:HORVU3Hr1G054100.7 cdna chromosome:IBSC_v2:chr3H:399273370:39... 26.3 24

EG:HORVU3Hr1G054100.2 cdna chromosome:IBSC_v2:chr3H:399272359:39... 26.3 24

EG:HORVU3Hr1G074680.1 cdna chromosome:IBSC_v2:chr3H:559145074:55... 26.3 24

EG:HORVU3Hr1G074680.4 cdna chromosome:IBSC_v2:chr3H:559145934:55... 26.3 24

EG:HORVU3Hr1G074680.3 cdna chromosome:IBSC_v2:chr3H:559145718:55... 26.3 24

EG:HORVU3Hr1G074680.2 cdna chromosome:IBSC_v2:chr3H:559145713:55... 26.3 24

EG:HORVU4Hr1G064940.1 cdna chromosome:IBSC_v2:chr4H:543015921:54... 26.3 24

EG:HORVU3Hr1G024500.1 cdna chromosome:IBSC_v2:chr3H:94783386:947... 26.3 24

EG:HORVU3Hr1G088850.6 cdna chromosome:IBSC_v2:chr3H:626143467:62... 26.3 24

EG:HORVU3Hr1G088850.3 cdna chromosome:IBSC_v2:chr3H:626143461:62... 26.3 24

EG:HORVU3Hr1G088850.13 cdna chromosome:IBSC_v2:chr3H:626145038:6... 26.3 24

EG:HORVU3Hr1G088850.10 cdna chromosome:IBSC_v2:chr3H:626143648:6... 26.3 24

EG:HORVU3Hr1G088850.12 cdna chromosome:IBSC_v2:chr3H:626144754:6... 26.3 24

EG:HORVU3Hr1G085680.27 cdna chromosome:IBSC_v2:chr3H:613630928:6... 26.3 24

EG:HORVU3Hr1G085680.18 cdna chromosome:IBSC_v2:chr3H:613628803:6... 26.3 24

EG:HORVU3Hr1G085680.19 cdna chromosome:IBSC_v2:chr3H:613628854:6... 26.3 24

EG:HORVU3Hr1G085680.17 cdna chromosome:IBSC_v2:chr3H:613628782:6... 26.3 24

EG:HORVU3Hr1G085680.31 cdna chromosome:IBSC_v2:chr3H:613631028:6... 26.3 24

EG:HORVU3Hr1G085680.24 cdna chromosome:IBSC_v2:chr3H:613628973:6... 26.3 24

EG:HORVU7Hr1G037210.1 cdna chromosome:IBSC_v2:chr7H:88750445:887... 26.3 24

EG:HORVU5Hr1G029490.2 cdna chromosome:IBSC_v2:chr5H:177481469:17... 26.3 24

EG:HORVU5Hr1G029490.1 cdna chromosome:IBSC_v2:chr5H:177481398:17... 26.3 24

EG:HORVU5Hr1G029490.4 cdna chromosome:IBSC_v2:chr5H:177481475:17... 26.3 24

EG:HORVU6Hr1G025260.1 cdna chromosome:IBSC_v2:chr6H:91006140:910... 26.3 24

EG:HORVU3Hr1G081720.3 cdna chromosome:IBSC_v2:chr3H:595860866:59... 26.3 24

EG:HORVU3Hr1G081720.1 cdna chromosome:IBSC_v2:chr3H:595860531:59... 26.3 24

EG:HORVU3Hr1G081720.4 cdna chromosome:IBSC_v2:chr3H:595860866:59... 26.3 24

EG:HORVU3Hr1G081720.2 cdna chromosome:IBSC_v2:chr3H:595860643:59... 26.3 24

EG:HORVU5Hr1G022470.2 cdna chromosome:IBSC_v2:chr5H:113581738:11... 26.3 24

EG:HORVU5Hr1G022470.3 cdna chromosome:IBSC_v2:chr5H:113581758:11... 26.3 24

>EG:HORVU5Hr1G005180.6 cdna chromosome:IBSC_v2:chr5H:8614116:8615298:-1 gene:HORVU5Hr1G005180

gene_biotype:protein_coding transcript_biotype:protein_coding

Length=1183

Score = 38.2 bits (19), Expect = 0.006

Identities = 19/19 (100%), Gaps = 0/19 (0%)

Strand=Plus/Minus

Query 1 AAGAAGTCCTTGTTCGCGC 19

|||||||||||||||||||

Sbjct 560 AAGAAGTCCTTGTTCGCGC 542

>EG:HORVU5Hr1G051970.1 cdna chromosome:IBSC_v2:chr5H:406435550:406436444:1 gene:HORVU5Hr1G051970

gene_biotype:protein_coding transcript_biotype:protein_coding

Length=895

Score = 38.2 bits (19), Expect = 0.006

Identities = 19/19 (100%), Gaps = 0/19 (0%)

Strand=Plus/Minus

Query 1 AAGAAGTCCTTGTTCGCGC 19

|||||||||||||||||||

Sbjct 507 AAGAAGTCCTTGTTCGCGC 489

>EG:HORVU6Hr1G076320.11 cdna chromosome:IBSC_v2:chr6H:525340759:525345025:1 gene:HORVU6Hr1G076320

gene_biotype:protein_coding transcript_biotype:protein_coding

Length=1582

Score = 30.2 bits (15), Expect = 1.5

Identities = 15/15 (100%), Gaps = 0/15 (0%)

Strand=Plus/Plus

Query 1 AAGAAGTCCTTGTTC 15

|||||||||||||||

Sbjct 35 AAGAAGTCCTTGTTC 4
