# Supplementary material for: Analysis of Barley Leaf Epidermis and Extrahaustorial Proteomes During Powdery Mildew Infection Reveals That the PR5 Thaumatin-Like Protein TLP5 Is Required for Susceptibility Towards Blumeria graminis f. sp. hordei
Source: Front Plant Sci. 2019 Oct 30;10:1138. doi: 10.3389/fpls.2019.01138 (PMC6831746; doi:10.3389/fpls.2019.01138)
Supplement: Supplementary Materials S4 — TLP5 expression, PTO sequences for gene silencing and impact of transient induced gene silencing on infection process. [file Table_4.docx]

**Supplementary materials 4**

Supplementary materials detailing primers used to amplify the transcripts of barley *HvMLO1*, *HvTLP5* and the reference genes *HvGAPDH* and *BghGAPDH*, by qRT-PCR, *TLP5* expression time course in infected and non-infected barley leaves. Data showing the response of cut leaves treated with PTO MLO1 and PTO PR5.1 including transcript and % secondary hyphae reduction, alongside the increased in % of DAB stained cells, is also shown.

**Contents**

Section [1](#_Toc9273375) [Primers used to amplify *MLO1, TLP5* and *BghGAPDH* by qPCR 1](#_Toc9273376)

Section 2 [*TLP5* and *BghGAPDH*  gene expression time course in infected and non-infected barley leaves 2](#_Toc9273378)

Section 3 [Impact on *TLP5* gene expression in excised leaves treated with the mock gene silencing treatment PTOZ and challenged with *Bgh* 3](#_Toc9273380)

Section 4 [Plant responses following *MLO1* silencing with PTO MLO.1 3](#_Toc9273382)

Section 5 [Plant responses following *TLP5* silencing with PTO PR5.1 4](#_Toc9273384)

Section 6 [% Germinated conidia associated with cells producing H_2_O_2_ compared to PTOZ 4](#_Toc9273386)

Section 7 [% of DAB stained cells relative to PTOZ treatment 5](#_Toc9273388)

# Section 1

# Primers used to amplify *MLO1, TLP5* and *BghGAPDH* by qPCR

| Gene target (Barley) | Accession ID | Primer sequence 5’ to 3’ | | Primer sequence reverse complement |
| --- | --- | --- | --- | --- |
| Hv*MLO1* | HORVU4Hr1G082710 | Fw | TCCAGAACGCGTTTCAGATGGC | GCCATCTGAAACGCGTTCTGGA |
|  |  | Rev | GCGTGTGGTAGCATTTCTTCAAGC | GCTTGAAGAAATGCTACCACACGC |
| Hv*TLP5* | HORVU5Hr1  G005180.6 | Fw | CACGGACATCACCAAGGATT^†^ | AATCCTTGGTGATGTCCGTG |
|  |  | Rev | TTGCCCTTGAAGAACATTGAG^†^ | CTCAATGTTCTTCAAGGGCAA |
| Hv*GAPDH* | CAA42901.1 | Fw | CTGATTGAGAAGGCTGATGGAT* | ATCCATCAGCCTTCTCAATCAG |
|  |  | Rev | AGAGCAGGAGCGTCATTGA* | TCAATGACGCTCCTGCTCT |
| Bgh*GAPDH* | BLGH_00691 | Fw | ATGAACTACAAGGCATCCTGTCA§ | TGACAGGATGCCTTGTAGTTCAT |
|  |  | Rev | TACCATGCGACTAGCTTAACAAAG§ | CTTTGTTAAGCTAGTCGCATGGTA |

*Pennington, H. G., Li, L. and Spanu, P. D. (2016). Normalization controls in qPCR. Molecular Plant Pathology, 17: 625-633. doi:10.1111/mpp.12300

^†^Scheler, B., Schnepf, V., Galgenmüller, C., Ranf, S., and Hückelhoven, R. (2016). Barley disease susceptibility factor RACB acts in epidermal cell polarity and positioning of the nucleus. *J. Exp. Bot.* 67, 3263–3275. doi:10.1093/jxb/erw141.

§Zhang, W. , Pedersen, C. , Kwaaitaal, M. , Gregersen, P. L., Mørch, S. M., Hanisch, S. , Kristensen, A. , Fuglsang, A. T., Collinge, D. B. and Thordal‐Christensen, H. (2012), Powdery mildew effector candidate targets PR17c. Molecular Plant Pathology, 13: 1110-1119. doi:10.1111/j.1364-3703.2012.00820.x

# Section 2

# *TLP5* and *BghGAPDH* gene expression time course in infected and non-infected barley leaves

*TLP5* and *BghGAPDH* expression time course in whole barley primary leaves following *Blumeria* infection over a 7 day period (**Inf**). Non-infected (**NI**) leaves were harvested in parallel as negative controls. The zero-time point was used for normalisation of relative amounts. Values shown are the mean of 3 independent biological replicates (each with 3 technical replicates), presented as the fold change of *TLP5* relatively to the barley housekeeping gene *HvGAPDH*.

| Time post innoculation | **0 dpi** | **1 dpi** | **2 dpi** | **3 dpi** | **4 dpi** | **5 dpi** | **6 dpi** | **7 dpi** |
| --- | --- | --- | --- | --- | --- | --- | --- | --- |
| Non infected leaves (NI) *TLP5* Log2 fold change | 0.0  (1.0) | 1.5  (x2.2) | 0.8  (x1.9) | -1.1  (x0.9) | -5.3  (x0.0) | -4.3  (x0.2) | -5.2  (x0.1) | -4.2  (x0.4) |
| SD (NI) | - | 3.8 | 0.9 | 1.5 | 1.0 | 3.4 | 3.8 | 4.6 |
| Infected leaves (Inf)  *TLP5* Log2 fold change | 0.0  (1.0) | 1.0  (x2.0) | -1.2  (x0.6) | 2.5  (x5.6) | 6.3  (x91) | 6.9  (x190) | 7.5  (x217) | 7.9  (x288) |
| SD (Inf) | - | 1.6 | 1.6 | 0.4 | 0.9 | 1.7 | 1.1 | 1.2 |
| Non infected leaves (NI) *BghGAPDH* Log2 fold change | 0.0 | -0.6 | -0.9 | -1.4 | -1.1 | -0.8 | -1.4 | -2.0 |
|  | 1.0 | (x0.7) | x(0.5) | (x0.4) | (x0.5) | (x0.6) | (x0.4) | (x0.3) |
| SD (NI) | - | 0.5 | 0.5 | 0.4 | 0.3 | 0.7 | 0.2 | 0.4 |
| Infected leaves (Inf) *BghGAPDH* Log2 fold change | 0.0 | 0.4 | 2.8 | 3.9 | 4.2 | 7.3 | 7.1 | 8.6 |
|  | 1.0 | (x1.3) | (x6.7) | (x15) | (x19) | (x162) | (x135) | (x400) |
| SD (Inf) | - | 2.0 | 9.3 | 12.7 | 17.0 | 215.9 | 140.4 | 479.2 |

# Section 3

# Impact on *TLP5* gene expression in excised leaves treated with the mock gene silencing PTOZ and challenged with *Bgh*

Excised leaves treated with PTOZ prior to handling and infection for the transient induced gene silencing assay (TIGS) were compared with otherwise unhandled infected leaves at 2 dpi. qPCR data are expressed as a relative fold change of *TLP5* expression relative to the reference gene *HvGAPDH*, and the “non-treated” *Blumeria* infected barley sample (2dpi) as reference sample.

|  |  | *Bgh* infected barley (2dpi) | PTOZ + *Bgh* infected barley (2dpi) |
| --- | --- | --- | --- |
| Fold Change *TLP5* | Average  SD  (SDM) | 1  -  - | 169.7  139.8  (40.4) |

# Section 4

# Plant responses following *MLO1* silencing with PTO MLO.1

The effect of the PTO MLO.1 treatment (for *MLO1* gene silencing) was compared to the PTOZ treatment (negative control) at different levels: *MLO1* transcript as measured by qRT-PCR (as described above); % Secondary hyphae to estimate the infection success rate at 44 hpi; % of cells producing hydrogen peroxide at 18-hpi (DAB stained cells). Fold change was calculated by comparing the value obtained for the PTO MLO.1 treatment to PTOZ treatment.

|  | PTOZ  treatment | PTO MLO1 treatment | SD PTOZ  treatment | SD PTO MLO1 treatment | Fold Change |
| --- | --- | --- | --- | --- | --- |
| Relative abundance of *MLO1* transcript | 1 | 0.53 | 0.15 | 0.15 | 0.53 |
|  | P<0.05 | |  |  |  |
| % Secondary Hyphae | 10.3 | 6.3 | 3.95 | 3.92 | 0.61 |
|  | P<0.01 | |  |  |  |
| % Germinated conidia associated with cells producing H_2_O_2_ | 9.8 | 25.4 | 4.02 | 2.50 | 2.59 |
|  | P<0.001 | |  |  |  |

# Section 5

# Plant responses following *TLP5* silencing with PTO PR5.1

The effect of the PTO PR5.1 treatment (for *TLP5* gene silencing) was compared to the PTOZ treatment (negative control) at different levels: *TLP5* transcript as measured by qRT-PCR (as described above); % Secondary hyphae to estimate the infection success rate at 44 hpi; % of cells producing hydrogen peroxide at 18-hpi (DAB stained cells). Fold change was calculated by comparing the value obtained for the PTO PR5.1 treatment to PTOZ treatment.

|  | PTOZ  treatment | PTO PR5.1 treatment | SD PTOZ  treatment | SD PTO PR5.1  treatment | Fold Change |
| --- | --- | --- | --- | --- | --- |
| Relative abundance of *TLP5* transcript | 1 | 0.52 | 0.03 | 0.36 | 0.52 |
|  | P<0.005 | |  |  |  |
| % Secondary Hyphae | 9.7 | 4.1 | 3.52 | 2.9 | 0.42 |
|  | P<0.001 | |  |  |  |
| % Germinated conidia associated with cells producing H_2_O_2_ | 9.8 | 26.1 | 4.02 | 2.78 | 2.66 |
|  | P<0.001 | |  |  |  |

# Section 6

# % Germinated conidia associated with cells producing H_2_O_2_ compared to PTOZ

The effect of PTO MLO1, PTO PR5.1 silencing on the % of cells producing hydrogen peroxide at 18-hpi (DAB stained cells) was compared to PTOZ (Sections 4,5). An additional two controls, water (no PTO) and a no treatment control were also compared to PTOZ. Statistical significance was determined by a one way ANOVA (F(2,85) = 130.35, p = <0.001) with post-hoc Tukey HSD t-tests.

| Treatment | % Germinated conidia associated with cells producing H_2_O_2_ | SD | Fold change | TUKEY HSD *p* value |
| --- | --- | --- | --- | --- |
| PTOZ | 9.84 | 4.03 | 1.00 | - |
| MLO1 | 25.48 | 2.50 | 2.59 | <0.001 |
| PR5.1 | 26.18 | 2.79 | 2.66 | <0.001 |
| Water | 9.43 | 5.21 | 0.96 | >0.05 |
| No Treatment | 6.28 | 2.60 | 0.64 | <0.05 |

# Section 7

# % of DAB stained cells relative to PTOZ treatment

The % of DAB stained cells at 18-hpi was also assessed by photographing each leaf once (3 sets of 6 biological replicates) (Supplementary materials 5) and counting the number of cells showing brown staining. Statistical significance was determined by a two-tailed homoscedastic T-test.

| Treatment | % cells producing H_2_O_2_ normalised to PTOZ | SEM | P value |
| --- | --- | --- | --- |
| PTOZ | 100% | 0% | - |
| MLO1 | 182% | 11% | P < 0.05 |
| PR5.1 | 162% | 10% | P < 0.05 |
| Water | 101% | 15% | P >0.1 |
| No Treatment | 66% | 18% | P >0.1 |
