# Supplementary material for: Analysis of Barley Leaf Epidermis and Extrahaustorial Proteomes During Powdery Mildew Infection Reveals That the PR5 Thaumatin-Like Protein TLP5 Is Required for Susceptibility Towards Blumeria graminis f. sp. hordei
Source: Front Plant Sci. 2019 Oct 30;10:1138. doi: 10.3389/fpls.2019.01138 (PMC6831746; doi:10.3389/fpls.2019.01138)
Supplement: Supplementary Materials S5 — Supplementary Figure 1 containing Micrographs of DAB stained leaves for H2O2 monitoring in response to Bgh infection in MLO1 and TLP5 silenced leaves. [file DataSheet_1.pdf]

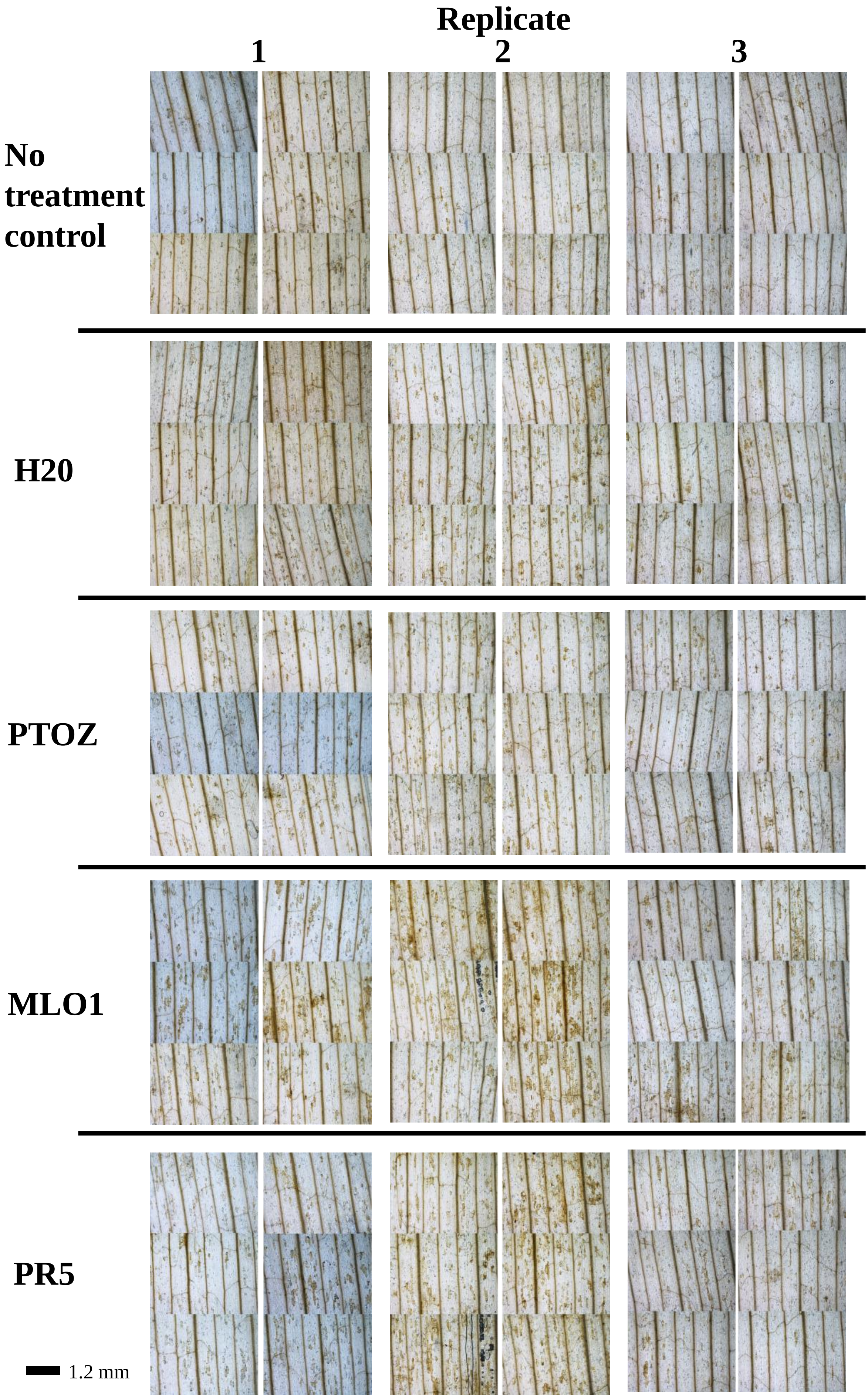

Supplementary figure 1: Photos of DAB stained leaves with a no treatment control, a water treatment (no PTO), and treated with PTOZ, PTO MLO1 and, PTO PR5. There are 6 biological replicates per panel, with 18 photos taken over three replicated per treatment. Photos were taken at 40x magnification using BF. Scale bar = 1.2mm .

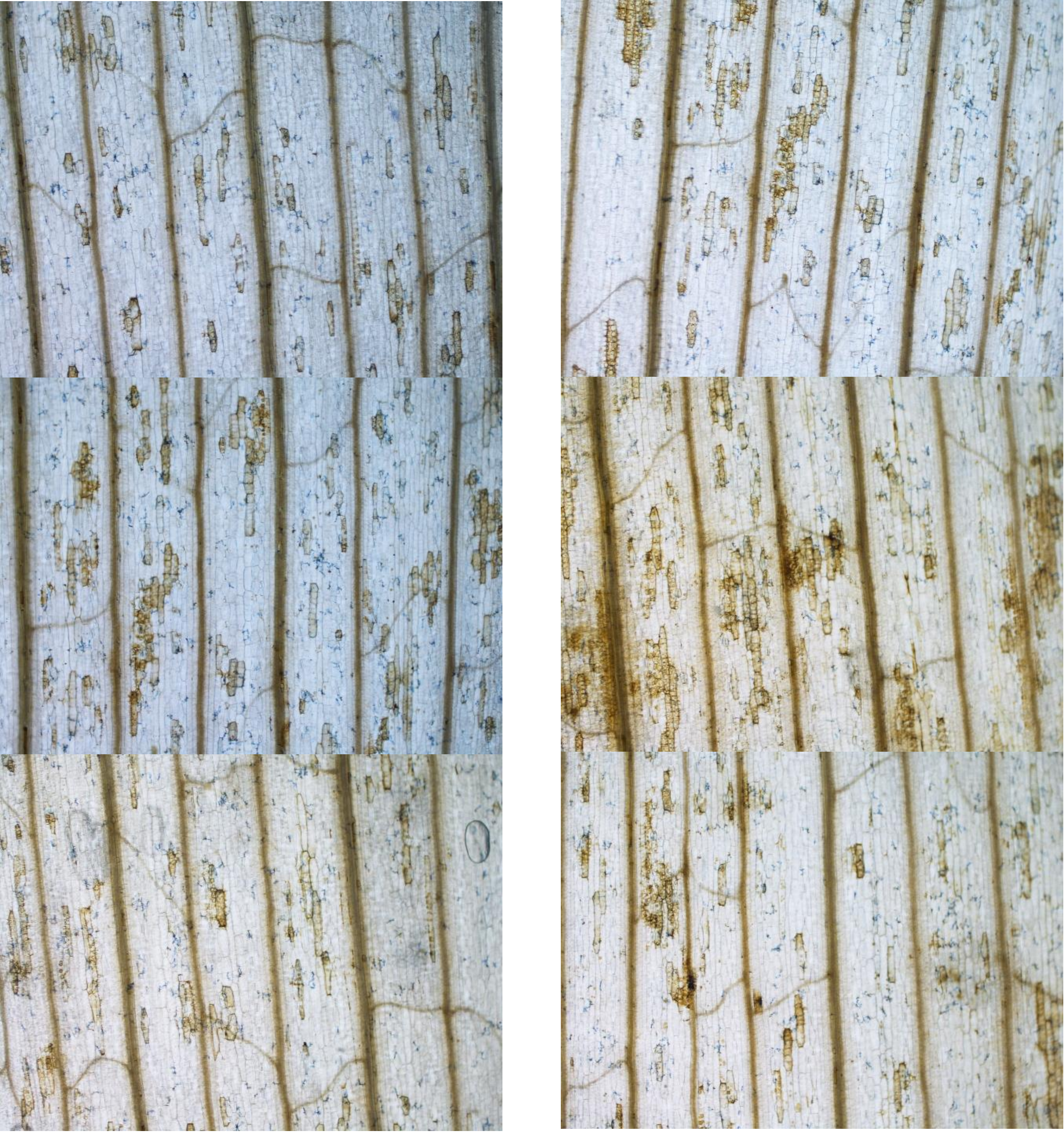

MLO1 rep 1

600 µm

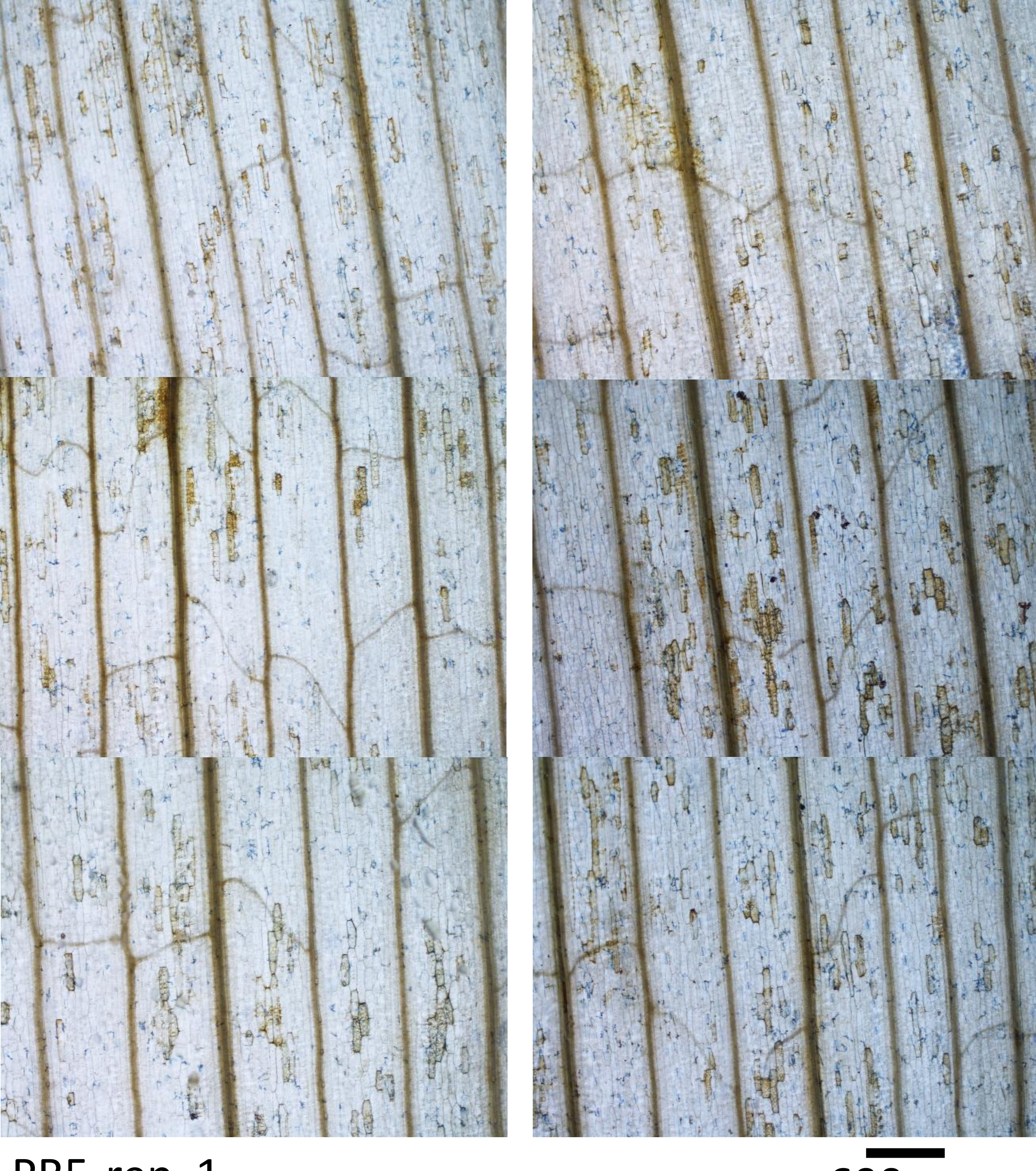

PR5 rep 1

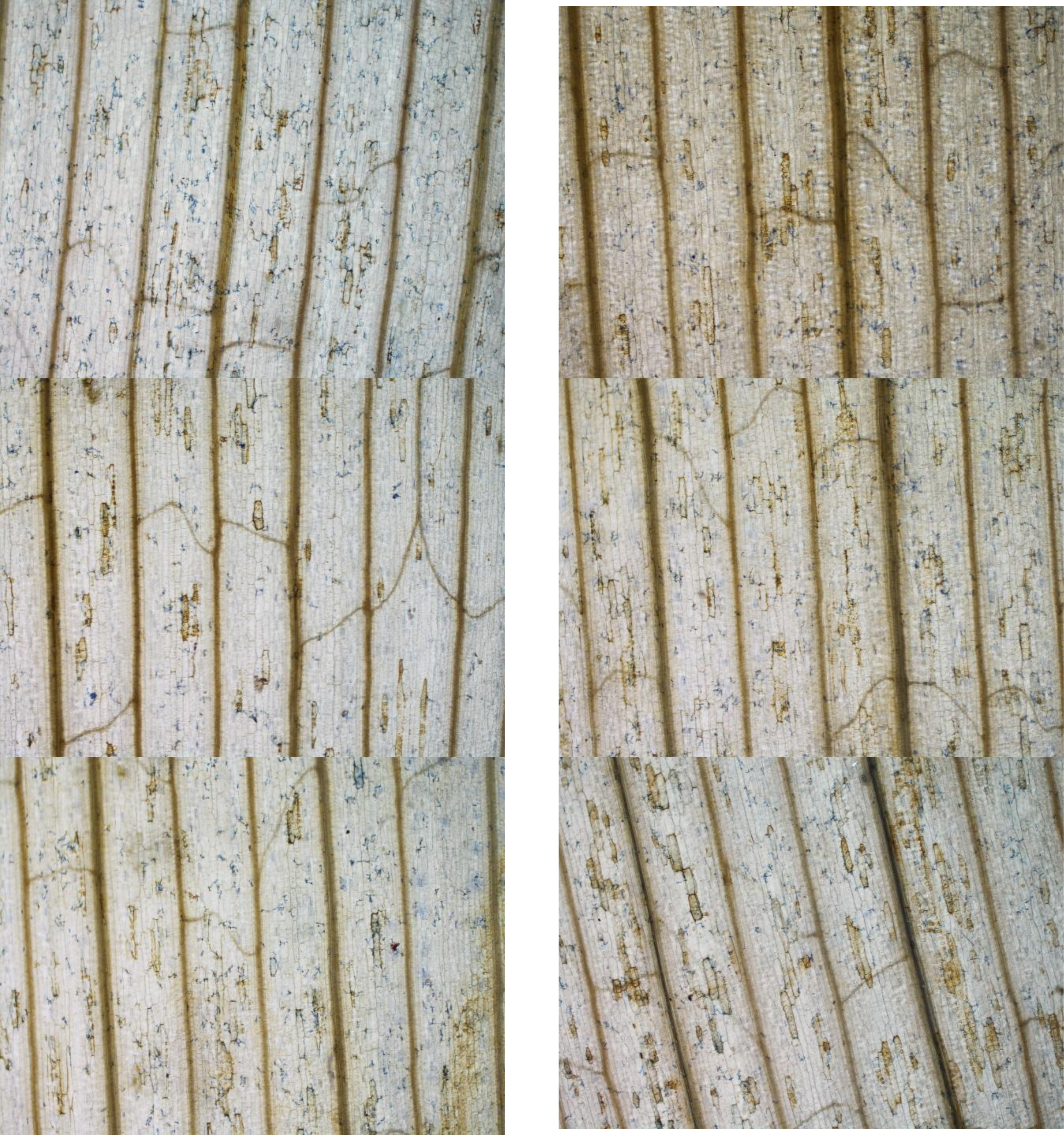

H20 rep 1

600 µm

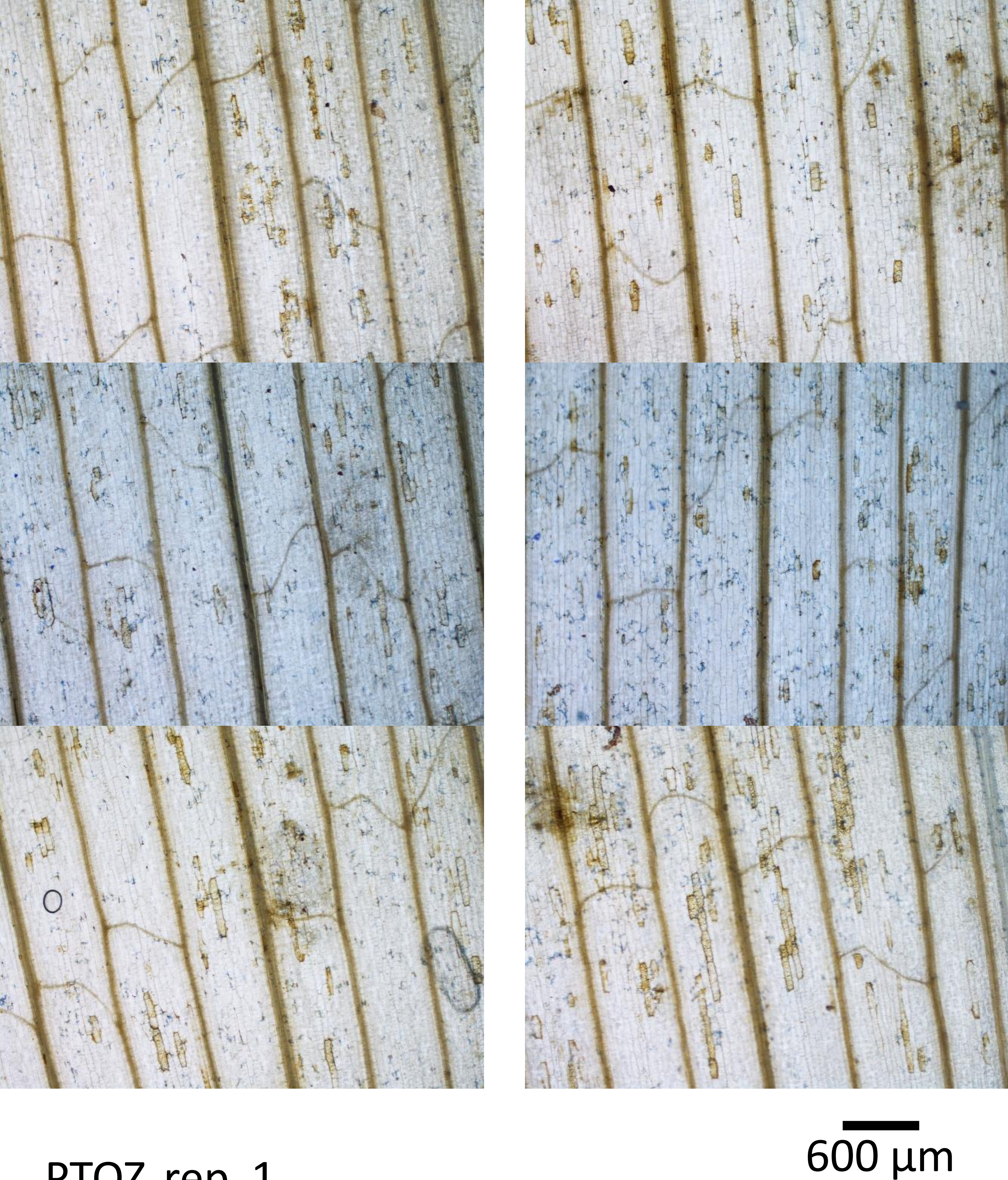

PTOZ rep 1

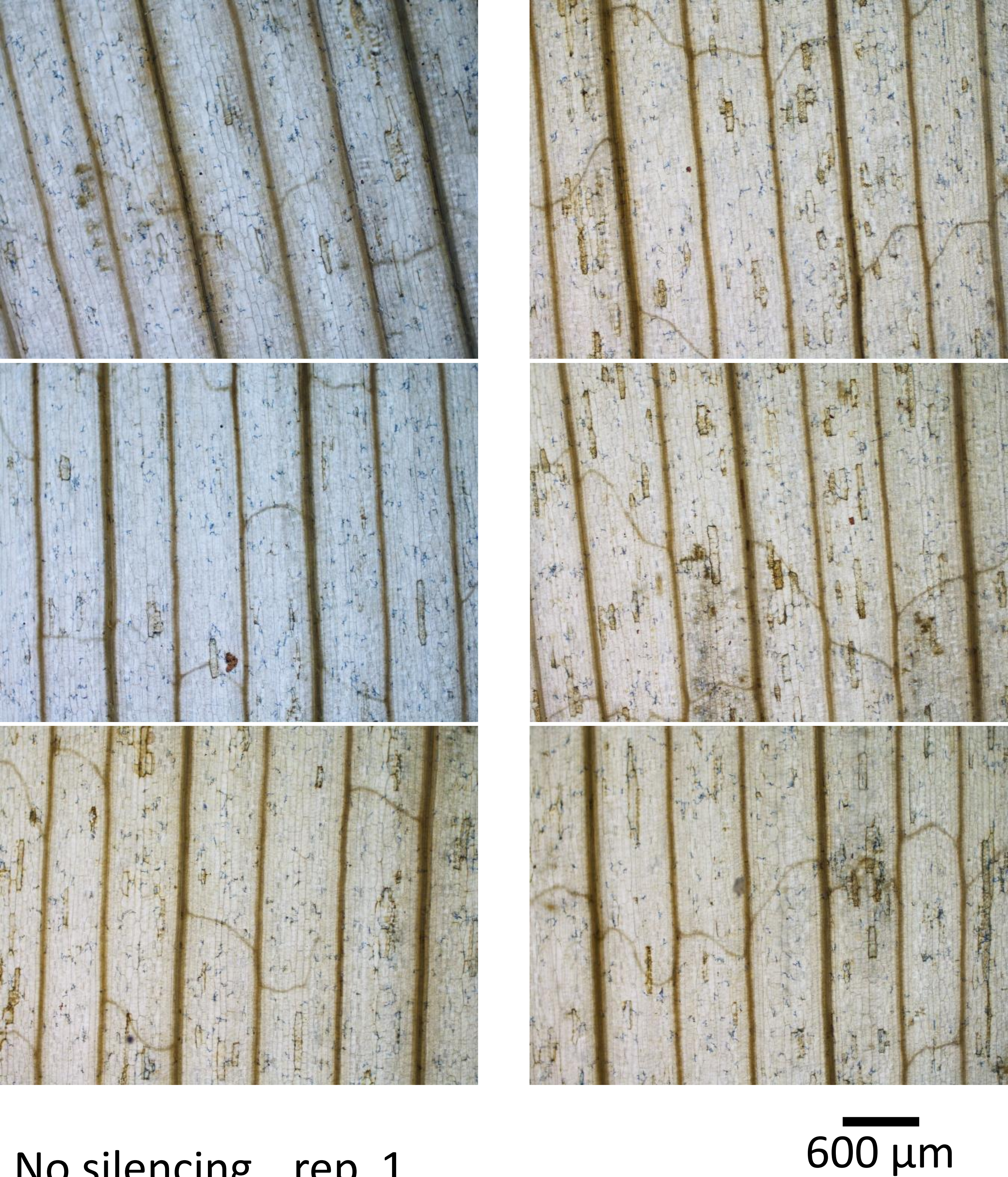

No silencing   rep 1
control

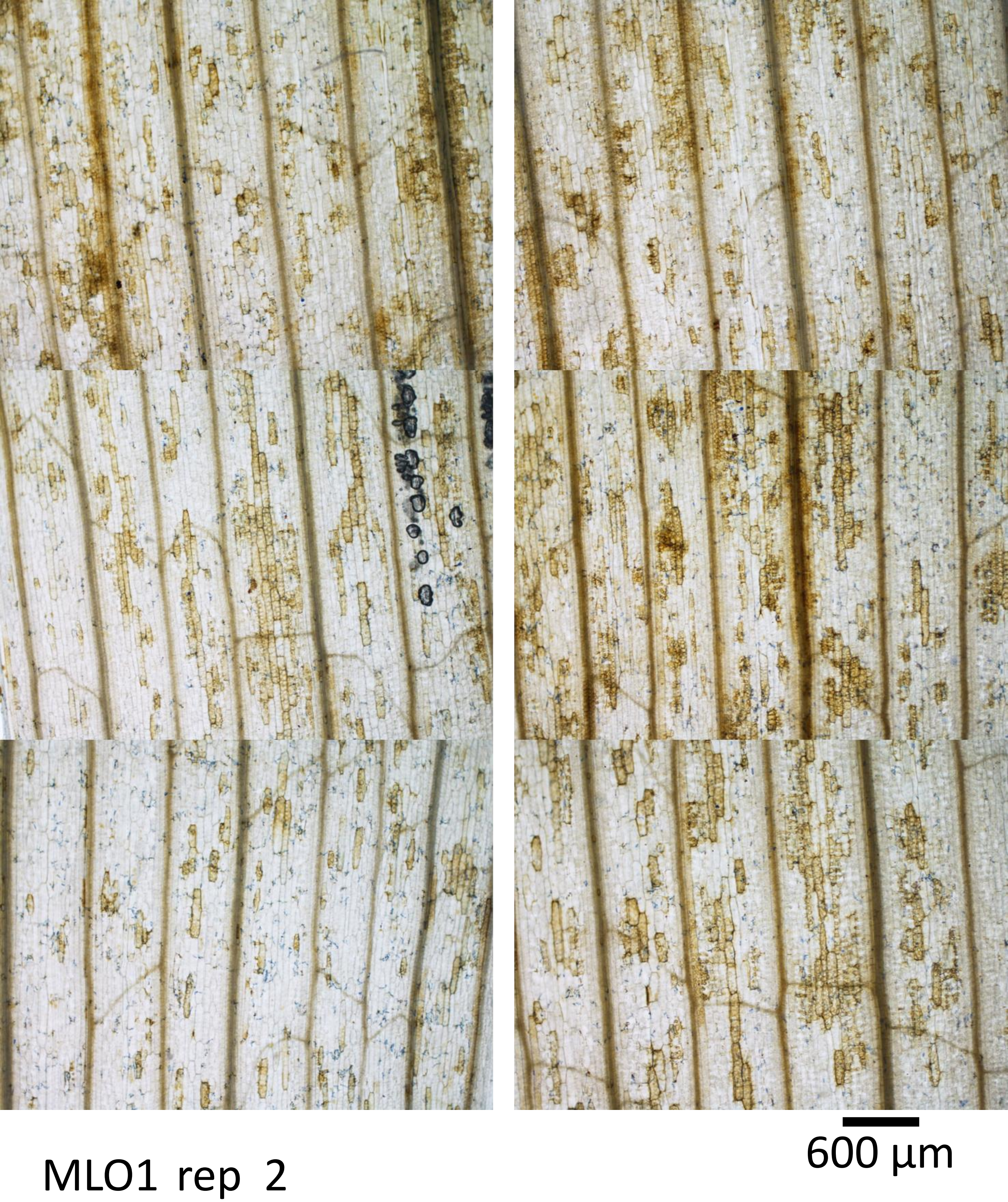

MLO1 rep 2

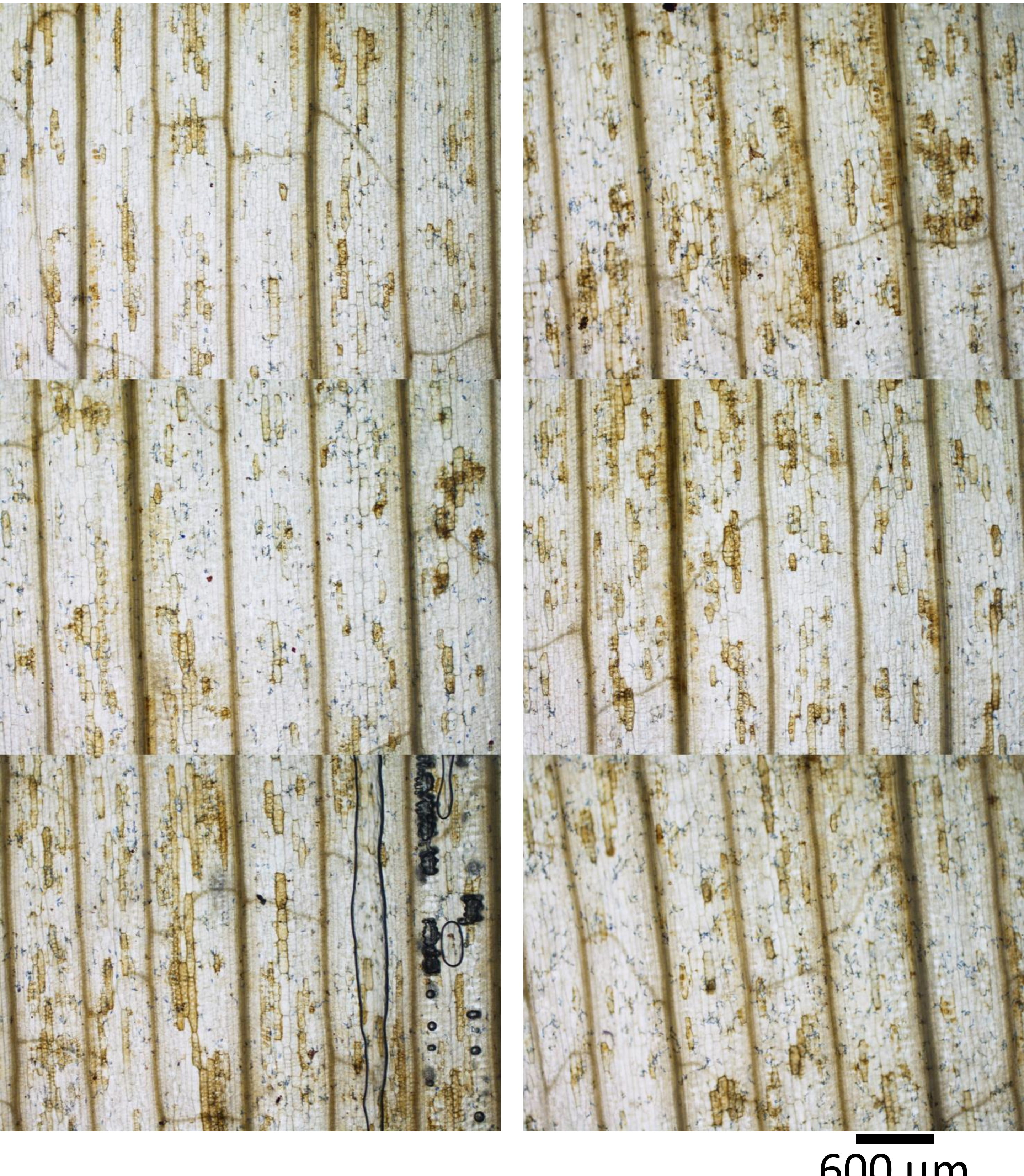

PR5 rep 2

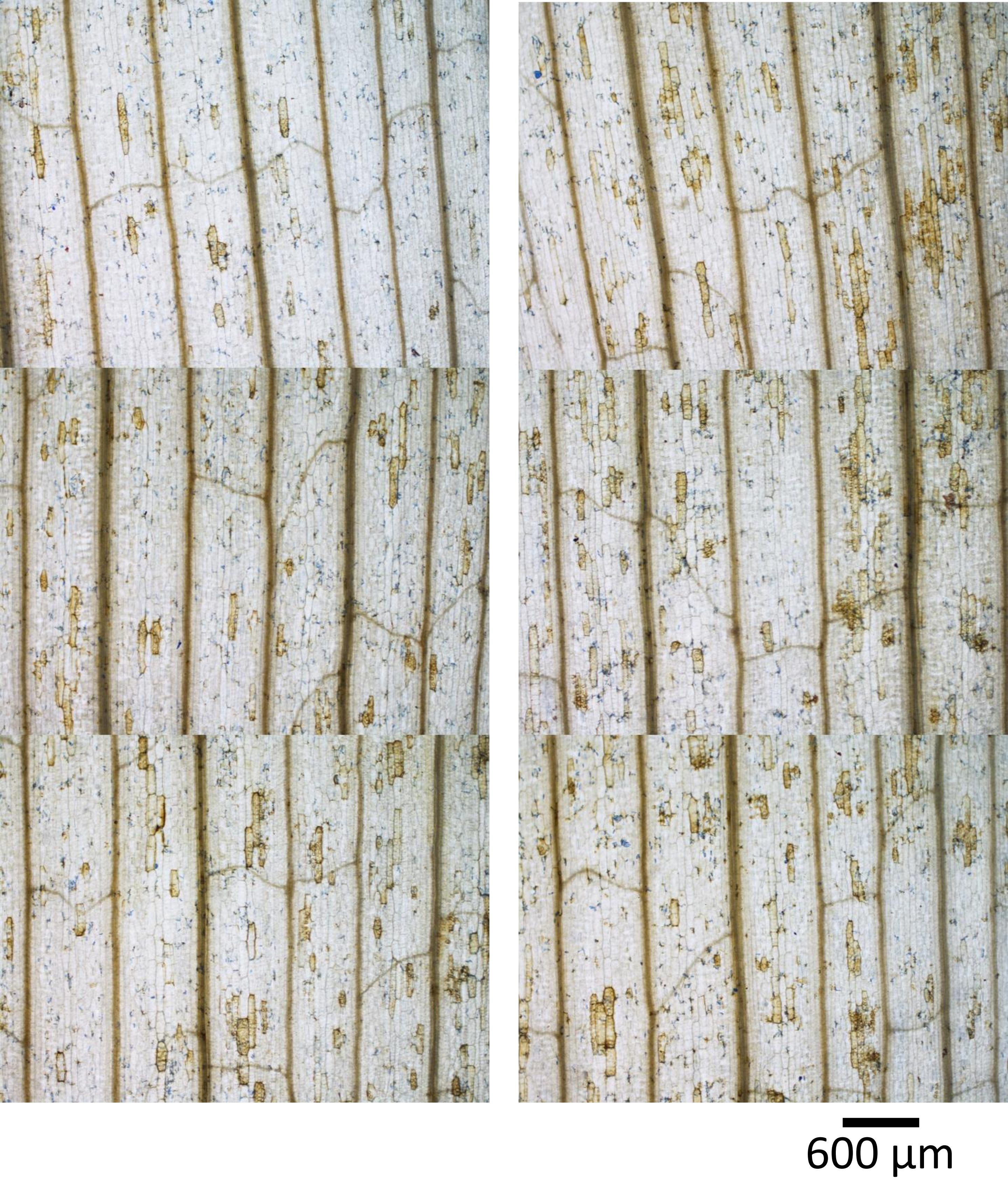

H20 rep 2

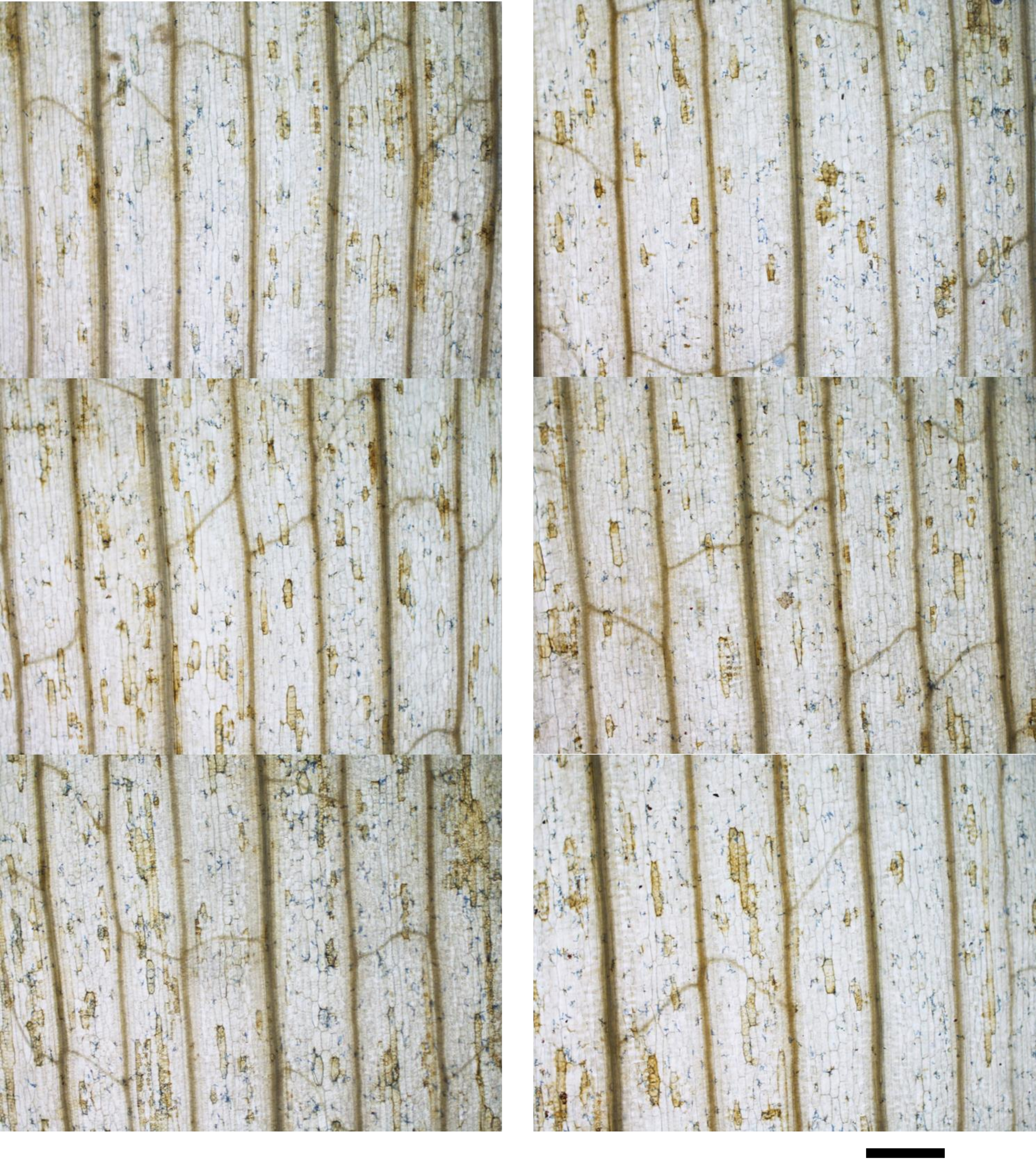

PTOz rep 2

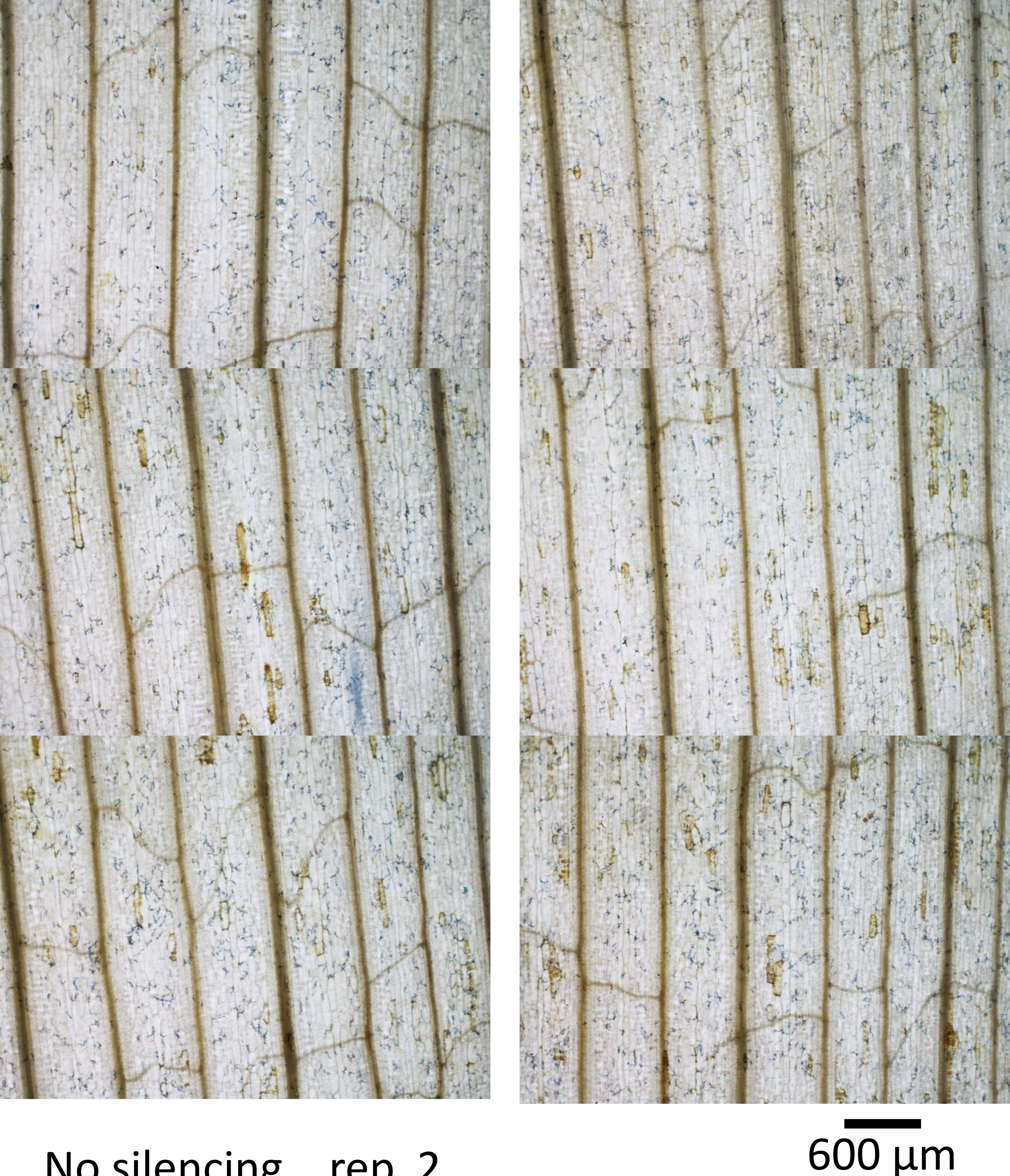

No silencing rep 2
control

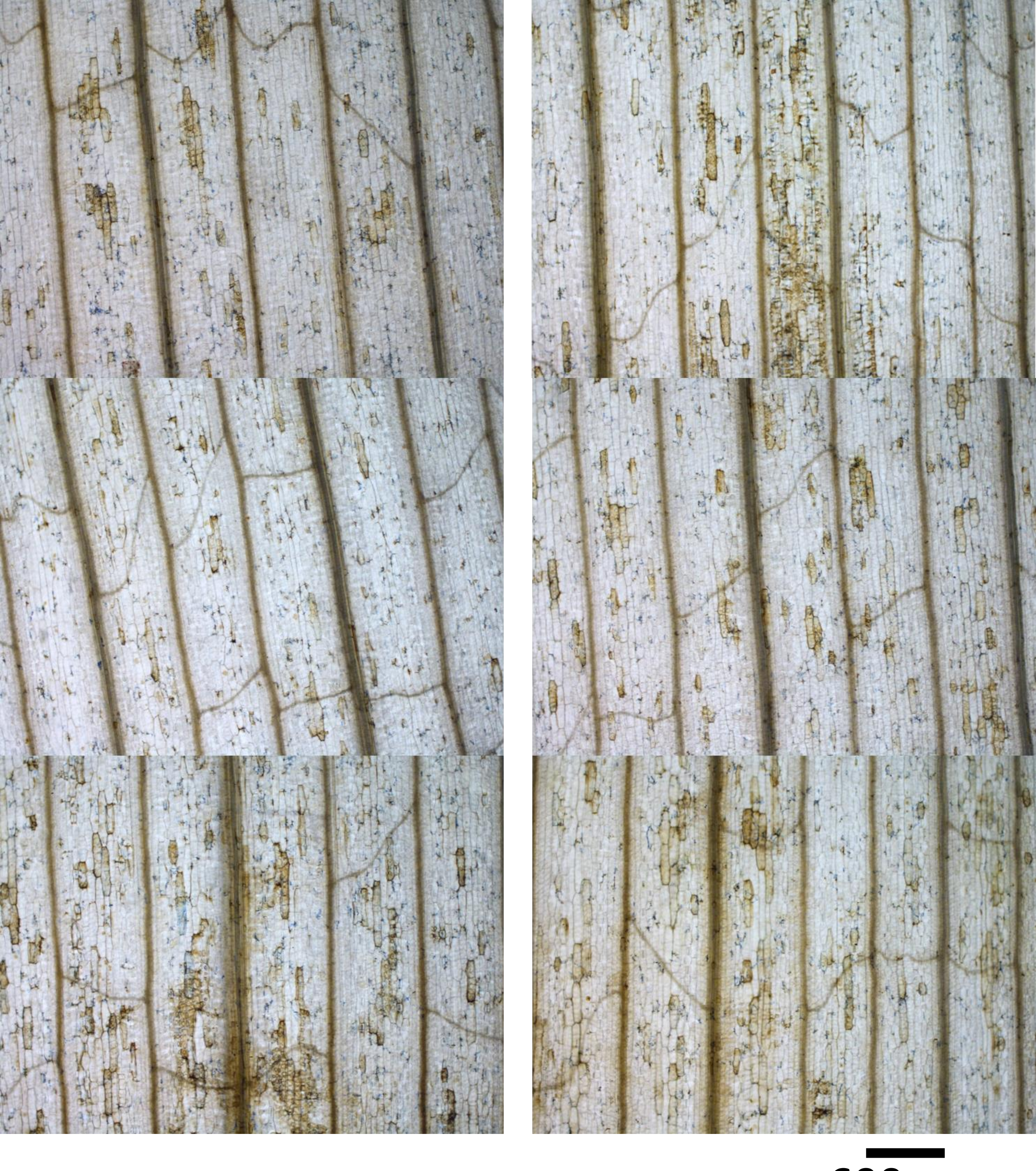

MLO1 rep 3

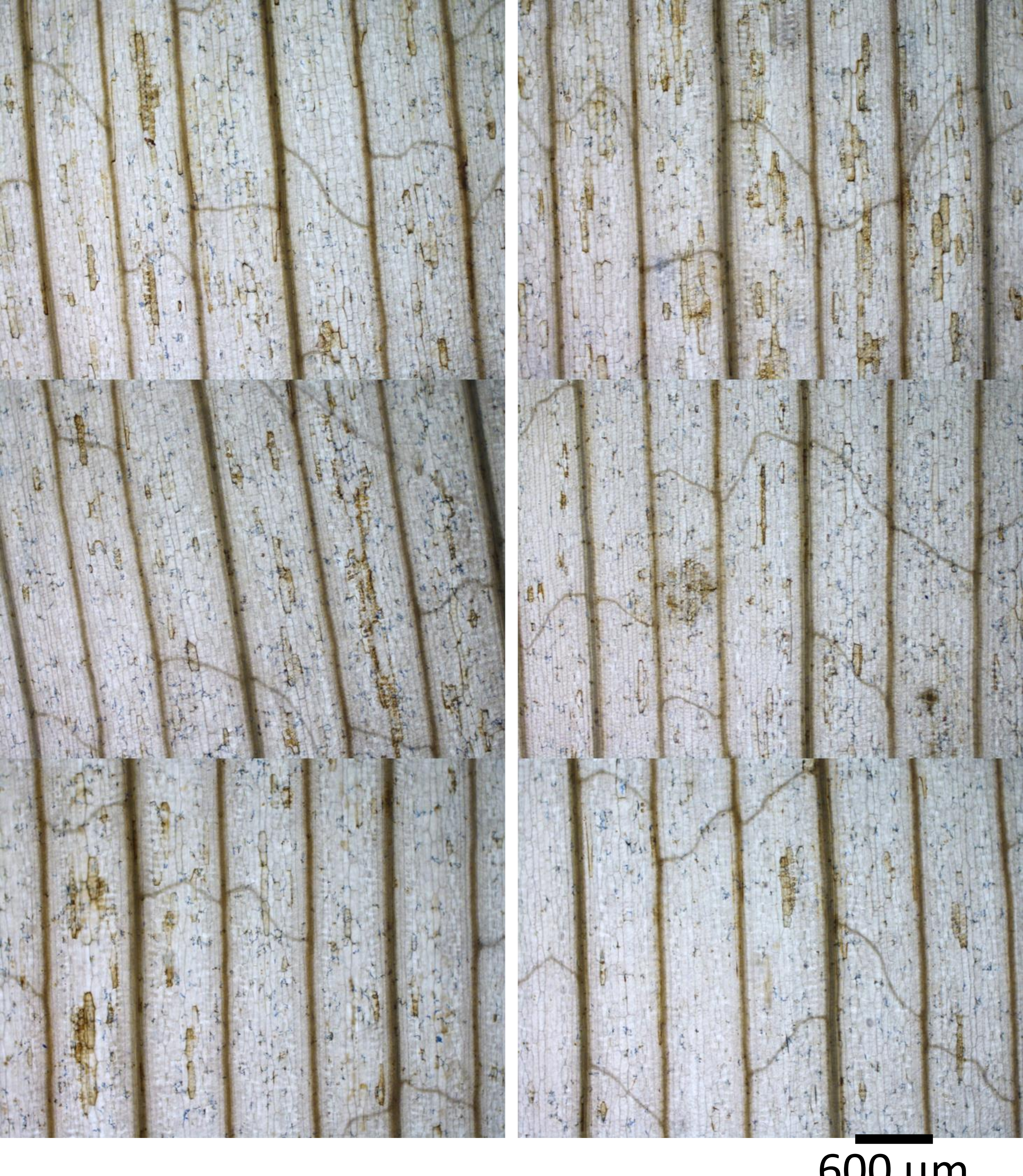

PR5 rep 3

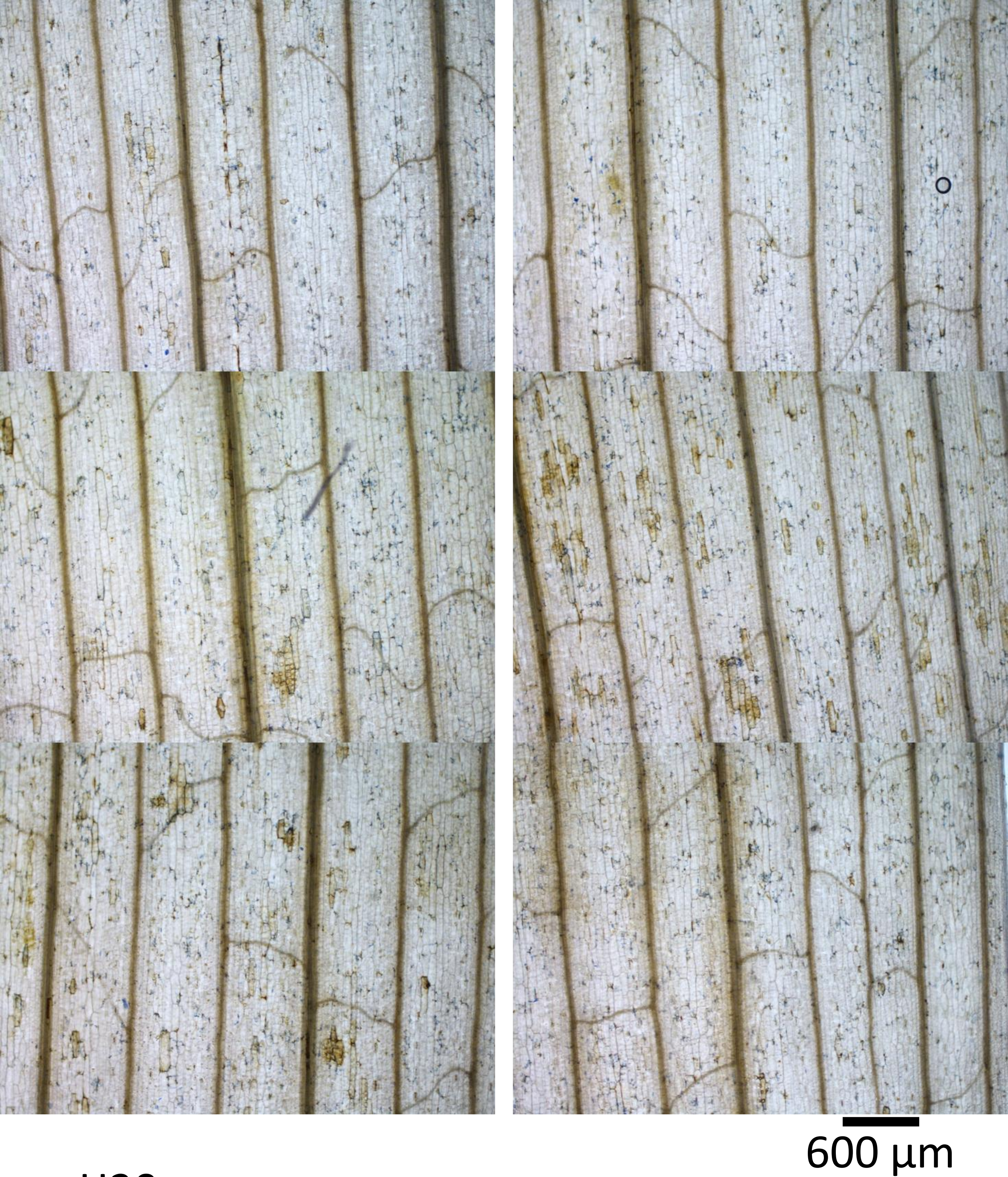

H20 rep 3

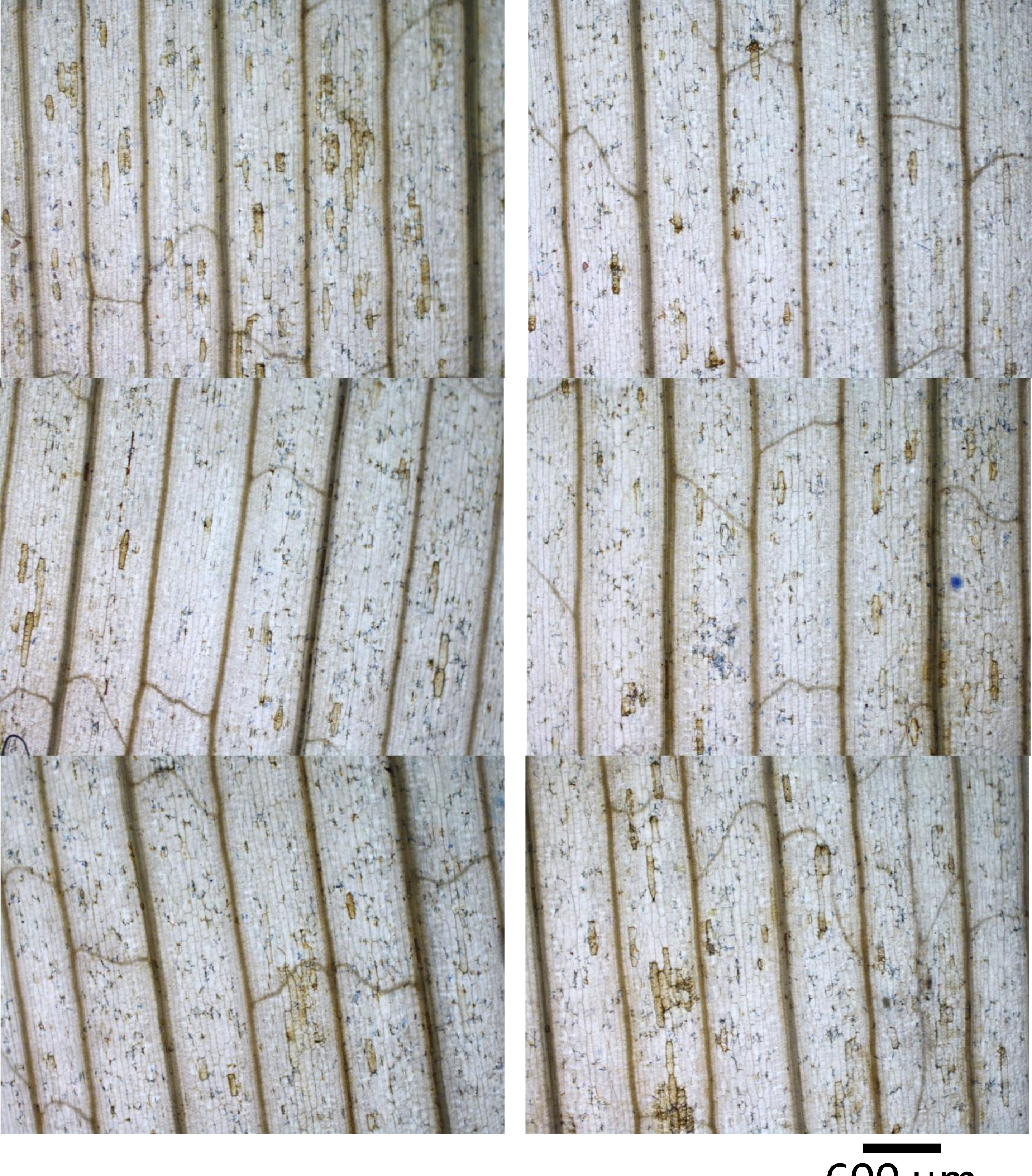

PTOz rep 3

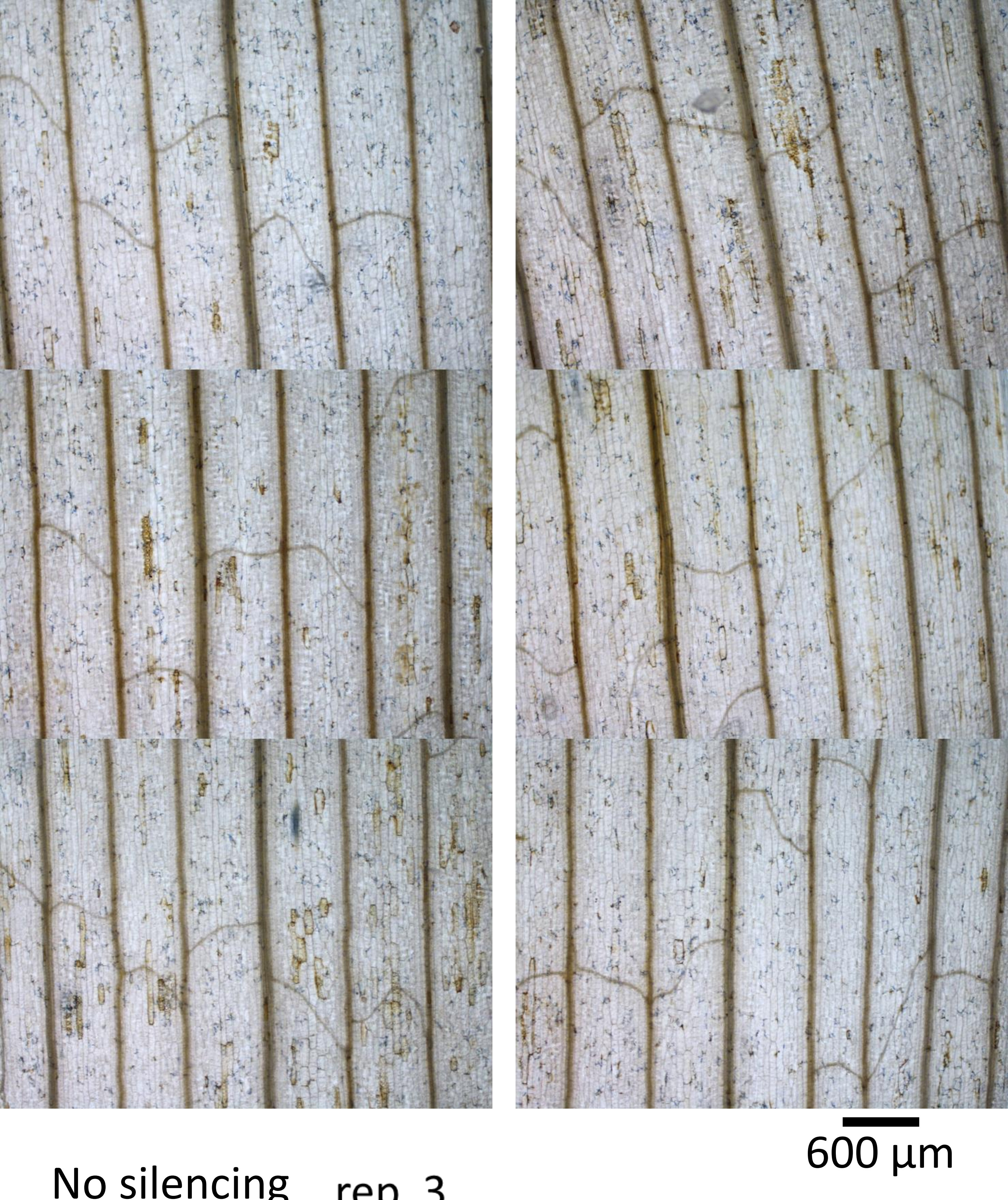

No silencing control rep 3
